# Draft genome of the gayal, *Bos frontalis*

**DOI:** 10.1093/gigascience/gix094

**Published:** 2017-10-05

**Authors:** Ming-Shan Wang, Yan Zeng, Xiao Wang, Wen-Hui Nie, Jin-Huan Wang, Wei-Ting Su, Newton O Otecko, Zi-Jun Xiong, Sheng Wang, Kai-Xing Qu, Shou-Qing Yan, Min-Min Yang, Wen Wang, Yang Dong, Dong-Dong Wu, Ya-Ping Zhang

**Affiliations:** 1 State Key Laboratory of Genetic Resources and Evolution, Yunnan Laboratory of Molecular Biology of Domestic Animals, Kunming Institute of Zoology, Chinese Academy of Sciences, Kunming 650223, China; 2 Kunming College of Life Science, University of Chinese Academy of Sciences, Kunming 650204, China; 3 Laboratory for Conservation and Utilization of Bio-resource, Yunnan University, Kunming 650091, China; 4 China National GeneBank, BGI–Shenzhen, Shenzhen 518083, China; 5 Laboratory of Animal Genetics, Breeding and Reproduction, Ministry of Agriculture of China, National Engineering Laboratory for Animal Breeding, College of Animal Science and Technology, China Agricultural University, Beijing 100193, China; 6 Yunnan Academy of Grassland and Animal Science, Kunming 650212, China; 7 College of Animal Science, Jilin University, Changchun 130062, China; 8 Yunnan Agricultural University, Kunming 650100, China; 9 Faculty of Life Science and Technology, Kunming University of Science and Technology, Kunming 650500, China

**Keywords:** *Bos frontalis*, genome assembly, annotation, phylogeny

## Abstract

Gayal (*Bos frontalis*), also known as mithan or mithun, is a large endangered semi-domesticated bovine that has a limited geographical distribution in the hill-forests of China, Northeast India, Bangladesh, Myanmar, and Bhutan. Many questions about the gayal such as its origin, population history, and genetic basis of local adaptation remain largely unresolved. *De novo* sequencing and assembly of the whole gayal genome provides an opportunity to address these issues. We report a high-depth sequencing, *de novo* assembly, and annotation of a female Chinese gayal genome. Based on the Illumina genomic sequencing platform, we have generated 350.38 Gb of raw data from 16 different insert-size libraries. A total of 276.86 Gb of clean data is retained after quality control. The assembled genome is about 2.85 Gb with scaffold and contig N50 sizes of 2.74 Mb and 14.41 kb, respectively. Repetitive elements account for 48.13% of the genome. Gene annotation has yielded 26 667 protein-coding genes, of which 97.18% have been functionally annotated. BUSCO assessment shows that our assembly captures 93% (3183 of 4104) of the core eukaryotic genes and 83.1% of vertebrate universal single-copy orthologs. We provide the first comprehensive *de novo* genome of the gayal. This genetic resource is integral for investigating the origin of the gayal and performing comparative genomic studies to improve understanding of the speciation and divergence of bovine species. The assembled genome could be used as reference in future population genetic studies of gayal.

## Data Description

### Background

**Figure 1:**
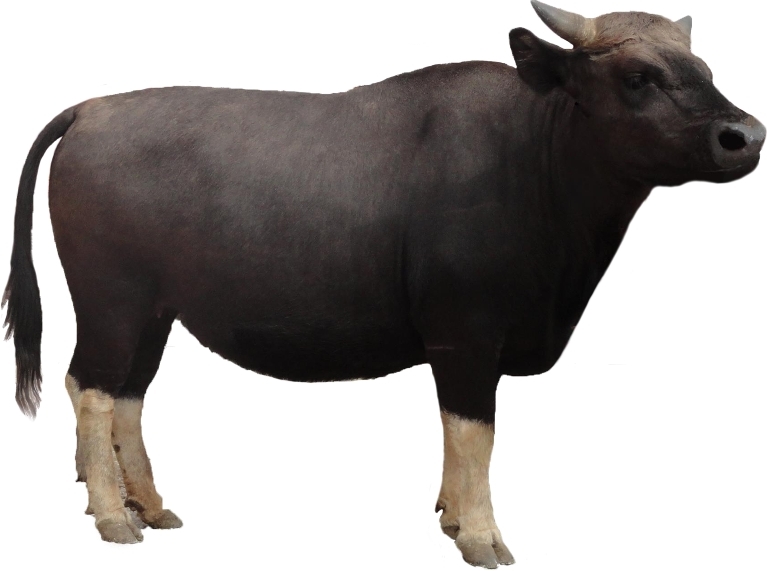
A picture showing a female gayal (*Bos frontalis*, provided by Kai-Xing Qu).

The gayal is a large-sized endangered semi-domesticated bovine species belonging to the family Bovidae, tribe Bovini, group Bovina, genus *Bos*, and species *Bos frontalis* (NCBI Taxon ID: 30 520). It is also called the mithan or mithun. Its distribution spans eastern Bhutan through the Arunachal Pradesh in India to the Naga and Chin hills in the Arakan Yomarange region that defines the borders between India, Bangladesh, Myanmar, and China [1, 2]. The gayal has unique characters and appearances compared to gaur, cattle, and other bovine species [3]. These features include a bony dorsal ridge on the shoulder and white stockings on all 4 legs (Figure 1). It has been previously held that gayal was domesticated from gaur and/or from a hybrid descendant from crossing domestic cattle (*B. indicus* or *B. taurus*) and wild gaur [2, 4, 5]. Karyotype analysis indicates that the Indian gayal has a 2n = 58 karyotype, same as the local gaur (2n = 58) [6, 7], but different from Chinese and Malaysian gaurs (*B. gaurus*, 2n = 56) as well as domesticated cattle (*B. indicus* and *B. taurus*, 2n = 60) [2, 6–10]. Phylogenetic analyses in multiple studies based on mtDNA or Y-chromosomal DNA place gayal in conflicting clustering positions with respect to cattle, zebu, and wild gaur. For example, Chinese gayal, or Dulong cattle, are known to harbor zebu or taurine mtDNA footprints, suggesting hybrid origin [5, 11], and more studies have shown a high mtDNA and Y-chromosomal DNA sequences similarity between gayal and guar [12–15]. One study has even placed the gayal as a distinct and separate species/subspecies [16]. In contrast, phylogenetic analyses based on single nucleotide polymorphisms (SNPs) from 20 randomly selected single-copy gene orthologs of *B. taurus*, *B. mutus* (wild yak), and *Bubalus bubalis* placed Chinese gayal off the *B. mutus* and *B. taurus* clade, indicating that gayal is distinct from the modern domestic cattle, *B. taurus* [5]. These authors further demonstrated from mtDNA analysis that the gayal is the most proximal to domesticated cattle (*B. taurus* and *B. indicus*), suggesting that the gayal could be a hybrid emanating from crossing of male wild gaur and female domestic cattle [5]. These differences illustrate the existence of unresolved uncertainties regarding the origin of gayal.

Research has revealed a high genomic divergence among bovine species [17, 18]. Consequently, mapping of resequencing data from 1 bovine species onto the reference genome of different species (for instance, gayal vs cattle) creates avenues for biases and/or errors in sequence alignment and SNP calling procedures. This challenge extends to species of great research interest like gayal, which so far have no *de novo* assembled reference genome. For instance, Mei et al. recently reported a whole-genome sequencing (resequencing) of Chinese gayal [5]. In their analysis, they retrieved variants based on mapping gayal sequencing reads (×13.06) to the cattle reference genome. Importantly, hydride gayals are hard to distinguish only through morphological characterization, yet Mei et al. did not examine the karyotype of the gayal they resequenced. In contrast to the gayal, *de novo* genome assembly has been accomplished for related species like cattle (*Bos taurus*) [19], yak (*Bos grunniens*) [17], wisent (*Bison bonasus*) [20], North American bison (*Bison bison*) [21], zebu (*Bos indicus*) [22], and water buffalo (*Bubalus bubalis*) [23]. This represents a critical resource toward mitigating the challenges inherent in resequencing approaches and provides great opportunities to refine the evolutionary history of bovine species. In this study, we for the first time report the draft genome assembly of the gayal with a high sequencing depth generated on the Illumina genome sequencing platform. This valuable resource is important to the research of the origin and evolution of this species, which has been classified as endangered by the International Union for Conservation of Nature (IUCN).

### Sample collection and sequencing

**Figure 2:**
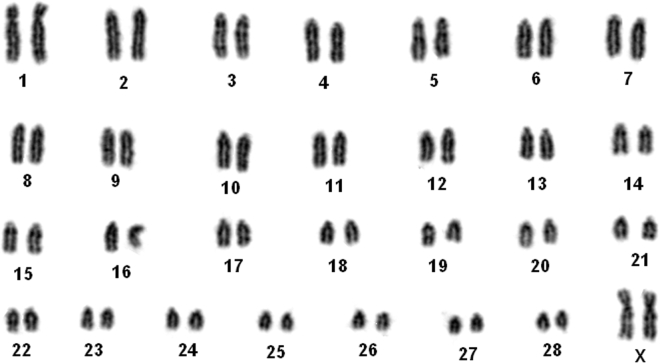
Karyotype of the gayal used for genome sequencing (provided by Wen-Hui Nie).

The gayal (NCBI taxonomy ID: 30 520) used for genome sequencing came from a Dulong in Yunnan province, China (Figure 1). It was kept at Yunnan Academy of Grassland and Animal Science for breeding and research purposes. Karyotype examination showed that it has 2n = 58 chromosomes (Figure 2). We extracted total genomic DNA from skin fibroblast cell lines of the gayal using the Qiagen Blood and Tissue Kit (Qiagen, Valencia, CA, USA) according to the manufacturer's instructions. The cells are maintained at the Cell Bank of Kunming Institute of Zoology (specimen ID: KCB201042). A total of 17 paired-end genomic sequence libraries were constructed with a gradient insert size ranging from 180 bp to 20 kb, and sequencing was carried out on the Illumina HiSeq 2000 platform according to the manufacturer's instructions. For short insert size libraries (180 bp, 250 bp, 450 bp, and 600 bp), sequencing was performed at the Central Laboratory of Kunming Institute of Zoology with read lengths of 100 bp. Sequencing of long insert size libraries (800 bp, 2, 5, 10 and 20 kb) was conducted at BGI-Shenzhen with read lengths of 49 bp, except for the 800-bp insert size library, which was sequenced with a read length of 85 bp. A total of 350.38 Gb of raw sequence data has been generated in our study (Additional file 1: Table S1). Before assembly, we performed strict quality control by removing poor-quality reads and/or bases using scripts from SOAPec (version 2.02) [24]. Reads were shortened by 2 bp at both the head and tail. We dropped any read plus its corresponding paired end if it contained more than 30 low-quality bases or more than 5% unknown base (usually denoted by N). Reads with duplications and adapters were also removed. We corrected for sequencing errors using the k-mer (13 used in this study) frequency method in SOAPec (version 2.02) [24]. After filtering and correction, we retained 276.86 Gb of high-quality sequences for genome assembly (Additional file 1: Table S2).

### *De novo* assembly of gayal genome

**Figure 3:**
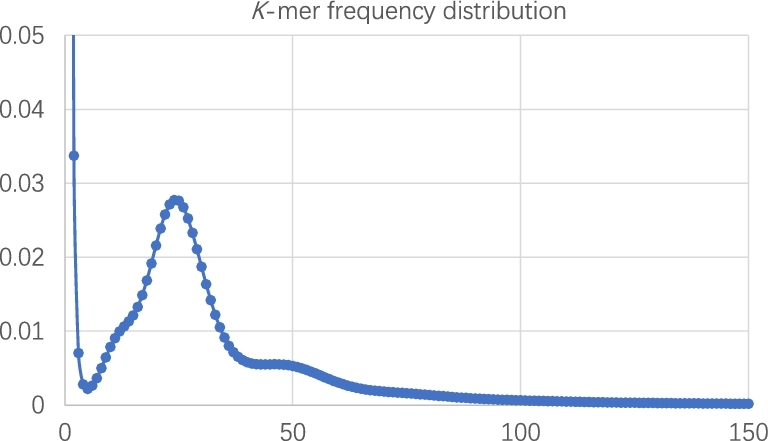
17-mer frequency distribution of sequencing reads.

In order to have a basic knowledge about the genome size and attributes of the gayal genome, we performed a 17-mer analysis using clean and high-quality sequences from 180 and 450 bp insert size libraries. We extracted the 17-mer sequences using sliding windows with a size of 17 bp and calculated the frequency of each 17-mer. A clear peak at ×25 with 2 upward convex signals apart from it is evident, suggesting high heterozygosity. The genome size for gayal is estimated to be 3.15 Gb (Figure 3; Additional file 1: Table S3).

We then performed *de novo* assembly of the gayal genome using Platanus (version 2.0; Platanus, RRID:SCR_015531) [25] in 3 steps: contig construction, scaffolding, and gap filling. To construct contigs based on short insert size libraries (180, 250, 450, 600, and 800 bp), we used Platanus (version 2.0) [25], which includes a series of procedures such as constructing de Bruijn graphs, clipping tips, merging bubbles, and removing low coverage links. In the scaffolding step, reads from both small and large insert libraries were mapped to contig sequences to construct scaffolds using distance information from read pairs. An additional local assembly of reads, with 1 end of a read pair uniquely aligned to a contig and the other end located within the gap, was performed using GapCloser (version 1.12; GapCloser, RRID:SCR_015026) [24]. These processes yielded a final draft gayal genome assembly with a total length of 2.85 Gb, contig N50 of 14.4 kb, and scaffold N50 of 2.74 Mb (Table 1). The assembled genome size is similar to that reported for cattle [26] and yak [17]. To assess the completeness of the assembled gayal genome, we performed BUSCO analysis (BUSCO, RRID:SCR_015008) [27] by searching against the arthropod universal benchmarking single-copy orthologs (BUSCOs, version 2.0). Overall, 85.2% and 7.8% of the 4104 expected vertebrate genes are identified in the assembled genome as complete and partial, respectively. Approximately 291 genes could be considered missing in our assembly. Of the expected complete vertebrate genes, 3434 and 60 are identified as single-copy and duplicated BUSCOs, respectively (Table 2). Our newly assembled gayal genome has a slightly lower completeness rate compared to genomes of yak [17], wisent [20], bison [21], zebu [22], and buffalo [23] (Table 2).

**Table 1:** Statistics of the completeness of the hybrid *de novo* assembly of *Bos frontalis* genome

Terms	Contig		Scaffold	
	Size	Number	Size	Number
N90	2461	211 577	158 610	1357
N80	5335	140 237	1 060 177	800
N70	8109	99 930	1 668 147	587
N60	11 044	71 764	2 170 469	437
N50	14 405	50 585	2 737 757	320
Max length	208 099		13 764 521	
Total length	2 669 378 334		2 848 570 279	
Total number		583 373		460 059
Average length	4575		6191	
Number ≥ 500 bp		394 757		116 481
Number ≥ 1000 bp		300 178		53 989
Number ≥ 2000 bp		229 796		19 915
Number ≥ 5000 bp		146 493		5387

**Table 2:** Statistics of the completeness of the assembled genomes for *Bos frontalis* and close related species by BUSCO (version 2)

Species	Terms	Complete (C)	Complete and single-copy (S)	Complete and duplicated (D)	Fragmented (F)	Missing (M)
Gayal	Number	3494	3434	60	319	291
	Proportion, %	85.14	83.67	1.46	7.77	7.09
Zebu	Number	3698	3644	54	158	248
	Proportion, %	90.11	88.79	1.32	3.85	6.04
Wisent	Number	3794	3763	31	180	130
	Proportion, %	92.45	91.69	0.76	4.39	3.17
Yak	Number	3841	3809	32	138	125
	Proportion, %	93.59	92.81	0.78	3.36	3.05
Buffalo	Number	3817	3780	37	142	145
	Proportion, %	93.01	92.11	0.90	3.46	3.53
Bison	Number	3779	3735	44	165	160
	Proportion, %	92.08	91.01	1.07	4.02	3.90

### Annotation of genomic repeat sequences in the gayal genome

To search for the repeated sequences in the gayal genome, including tandem repeats, interspersed repeats, and transposable elements (TE; e.g., LINE, SINE, LTR, DNA transposons), we leveraged both *de novo* and homolog-based methods as used in previous publications [28, 29]. For the homolog-based methods, we used RepeatMasker (RepeatMasker, RRID:SCR_012954) and RepeatProteinMask [30] to search against the known Repbase TE library (RepBase21.01) [31] and TE protein database, respectively. In the *de novo* method, Piler [32] and RepeatModeler (RepeatModeler, RRID:SCR_015027) [33] are used to generate a *de novo* gayal repeat library, which is subsequently used in Repeat-Masker to annotate repeats. TRF [34] is then employed to predict tandem repeats. The combined results show that a total of 1.37 Gb of non-redundant repetitive sequences are identified in the gayal genome, which account for 48.13% of the whole genome. The most predominant repeat is the long interspersed nuclear elements (LINEs), which account for 40.43% (1.15 Gb in total) of the genome (Table 3; Additional file 1: Table S4, Figure S1, Figure S2).

**Table 3:** Statistics of repeats in *Bos frontalis* genome

Type	Repeat size, bp	% of genome
Trf	17 696 175	0.62
Repeatmasker	868 885 926	30.50
Proteinmask	265 003 148	9.30
*De novo*	917 371 710	32.20
Total	1 371 023 312	48.13

### Gayal genome gene structure prediction

For gene structure prediction, we combined both *de novo* and homolog-based approaches to predict protein-coding genes in the gayal genome. In homolog-based method, gene sets from *Bos taurus* [19], *Canis familiaris* [35], *Homo sapiens* (ENSEMBL 80), *Sus scrofa* [36], *Rattus norvegicus* (ENSEMBL 80), and *Ovis aries* [37] were used as queries to search against the gayal genome (Additional file 1: Table S5). For the *de novo*–based method, AUGUSTUS (Augustus: Gene Prediction, RRID:SCR_008417) [38], Genescan (GENSCAN, RRID:SCR_012902) [39], and GlimmerHMM (GlimmerHMM, RRID:SCR_002654) [40] were used as engines to predict gene models. We then merged the gene prediction results derived from both methods using GLEAN [41] to generate a consensus gene set. In total, we have identified 26 667 protein-coding genes with a mean of 3.27 exons per gene (Table 4; Additional file 1: Figure S3). The lengths of genes, coding sequence (CDS), introns, and exons in gayal are comparable to those of the genomes used for homolog-based predictions (Additional file 1: Figure S3). In addition, we predicted non-coding RNA genes in the gayal genome. We used blast to search rRNA against the Human rRNA database, and tRNAscan-SE (tRNAscan-SE, RRID:SCR_010835) [42] to search tRNA in the genome sequences. We also used blast to search miRNA and snRNA via the Rfam database (release 11.0; Rfam, RRID:SCR_007891) [43]. We reveal a total of 2357 ribosomal RNA (rRNA), 29 821 transfer RNA (tRNA), 16 305 microRNAs (miRNA), and 1380 snRNA genes in the gayal genome (Additional file 1: Table S5).

**Table 4:** General statistics of predicted protein-coding genes

Gene set		Total	Exon number	CDS length, bp	mRNA length, bp	Exons per gene	Exon length, bp	Intron length, bp
Homolog	*Bos taurus*	19 666	141 323	1325	20 618	7.19	184	3118
	*Canis familiaris*	17 627	121 986	1323	20 802	6.92	191	3290
	*Homo sapiens*	24 783	146 172	1108	17 567	5.89	187	3360
	*Sus scrofa*	20 283	121 282	1142	16 288	5.97	191	3041
	*Rattus norvegicus*	17 988	117 965	1277	19 469	6.55	194	3273
	*Ovis aries*	20 947	147 367	1287	20 973	7.03	183	3261
*De novo*	AUGUSTUS	41 227	180 664	1127	22 786	4.38	257	6403
	GlimmerHMM	27 067	104 294	874	5433	3.85	226	1597
	Genescan	46 598	297 828	1321	36 828	6.39	206	6585
Glean (final)		26 667	87 392	1156	4996	3.27	352	1686

### Functional annotation of protein-coding genes

Gene functional annotation refers to searching functional motifs, domains, and possible biological processes by aligning translated gene coding sequences to known databases such as SwissProt and TrEMBL [44], NT database (from NCBI), Gene Ontology (GO, RRID:SCR_002811), and Kyoto Encyclopedia of Genes and Genomes (KEGG, RRID:SCR_012773) [45]. We have annotated all the protein-coding genes identified in this study to retrieve functional terms according to InterPro, KEGG, and GO terms. Overall, 81.74% (21 798), 54.56% (14 550), and 66.39% (17 704) genes show enrichment in InterPro, KEGG, and GO, respectively. In total, 25 916 protein-coding genes (97.18%) were successfully annotated for conserved functional motifs and functional terms (Additional file 1: Table S6).

### Phylogenetic analysis and divergence time estimation

**Figure 4:**
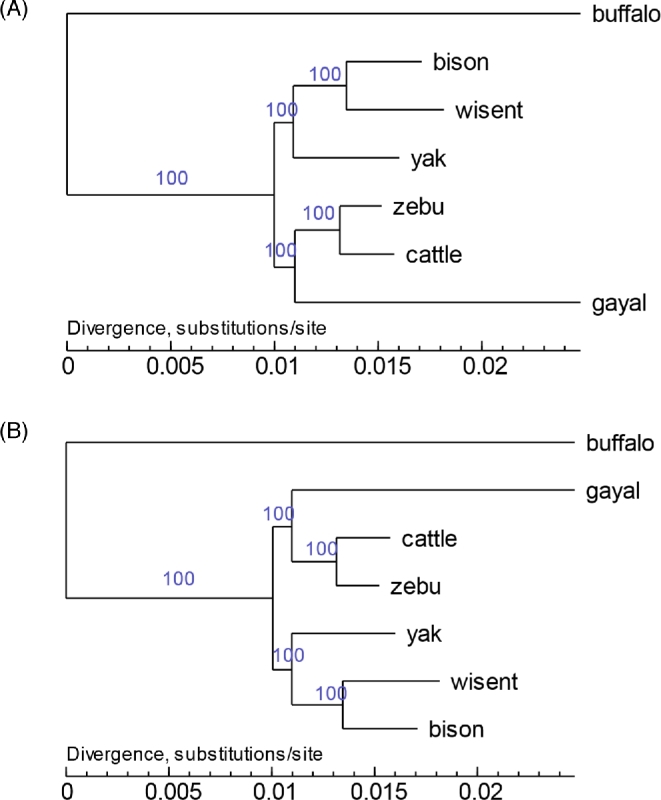
Phylogenetic trees of gayal and other bovine species. **(A)** Tree constructed based on maximum likelihood method. **(B)** Tree constructed using Bayesian inference.

To investigate the phylogenic position of gayal, we retrieved nucleotide and protein data for cattle (*Bos taurus*) [19], yak (*Bos grunniens*) [17], wisent (*Bison bonasus*) [20], bison (*Bison bison*) [21], zebu (*Bos indicus*) [22], and buffalo (*Bubalus bubalis*) [23] from the NCBI database. Gene ortholog relationships of gayal and other bovine species were identified by reciprocal blast searching with an e-value of 1e-7. Genes with alternative splicing variants are represented by the longest transcript. Multiple sequence alignment of the genes within 1 copy gene set were performed using the MUSCLE program (MUSCLE, RRID:SCR_011812) [46]. Aligned sequences were trimmed to remove potentially unreliably aligned regions and gaps using Gblocks [47]. Alignments with lengths shorter than 100 bp were also discarded. Four-fold degenerate sites were extracted and concatenated into a supergene. Modeltest [48] was used to select the best substitution model. MrBayes (MrBayes, RRID:SCR_012067) [49] and RaxML (RAxML, RRID:SCR_006086) [50] software were used to reconstruct the evolutionary relationships between species, and MEGA5 [51] was used to view the tree. From these analyses, gayal clusters with the common ancestor of cattle and zebu (Figure 4).

**Figure 5:**
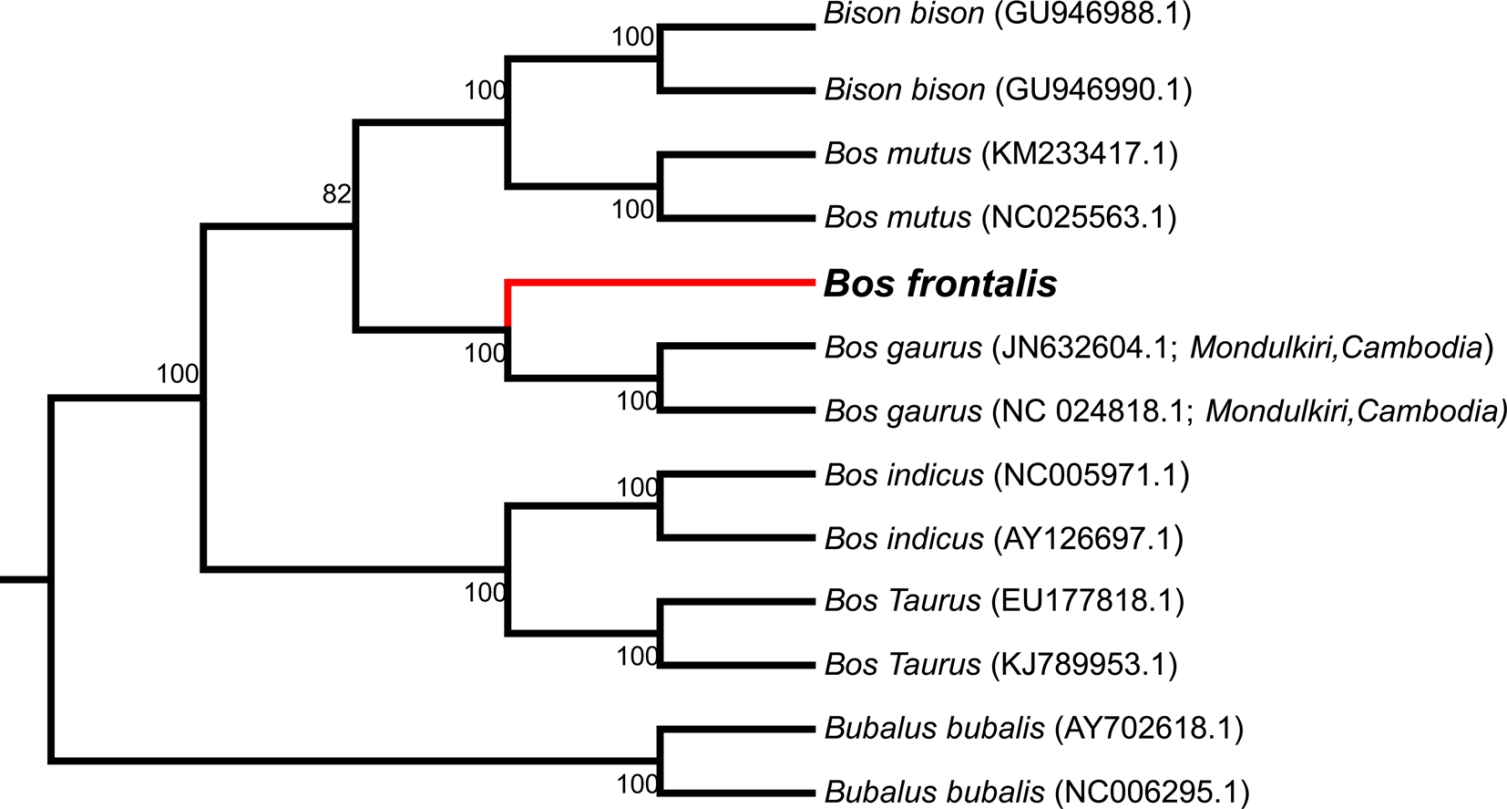
Maximum likelihood trees of gayal and other bovine species using whole complete mtDNA. IDs in parentheses are GenBank accession number.

**Figure 6:**
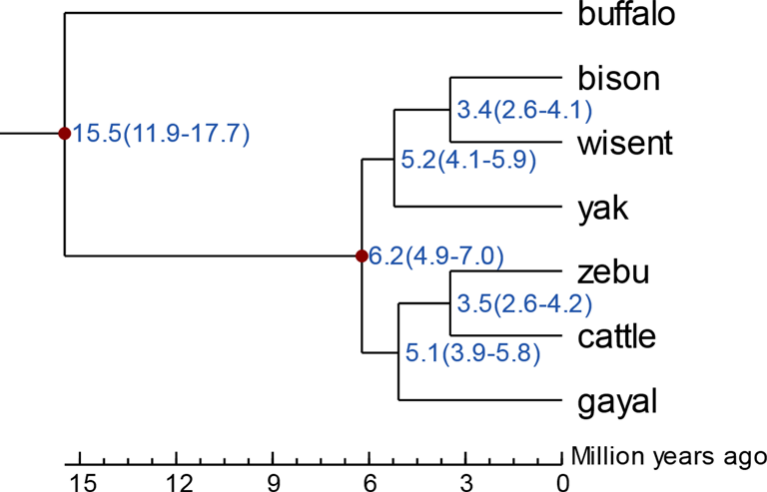
Divergence time estimated between gayal and other bovine species.

Additionally, we sequenced the complete mitochondrial DNA (mtDNA, the first complete mtDNA of the gayal submitted to GenBank: MF614103) using the Sanger sequencing method, due to the fact that next-generation sequencing methods have lower ability and accuracy in recovering repeat sequences [28, 52], particularly in regions with rich GC content like the D-loop. We then downloaded mtDNA sequences of gayal and other bovine species from GenBank for phylogenic analysis. As shown in Figure 5 and Figure S4, the gayal we sequenced clusters with gaur (Figure 5; Additional file 1: Figure S4). Our results from both whole-genome and mtDNA data differ from the conclusion made by Mei et al., who mapped gayal genome resequencing data to a bovine reference [5]. Furthermore, the MCMCTREE program, implemented using the PAML (PAML, RRID:SCR_014932) [53] package, was used to estimate divergence times. The JC69 model and correlated molecular clock rates (clock = 3) were used in the calculation. Calibration time for the common ancestor of buffalo and cattle obtained from the TimeTree database [54] was used to calibrate the divergence time. This analysis estimated the divergence time of gayal from cattle and zebu at approximately 5.1 million years ago (Figure 6).

In conclusion, we have constructed a *de novo* assembly of the gayal genome, and we describe its genetic attributes. To our knowledge, this is the first *de novo* assembled genome for this species. We also demonstrate that together with the genomes of other bovine species, the new gayal genome supports investigations concerning the origin, evolutionary history, and local adaptation of gayal. This resource is also important for the future conservation of this endangered species. In addition, the *de novo* gayal genome adds to the list of available bovine genomes and has advantages over resequenced genomes in allowing accurate whole-genome alignment and retrieving constraint and/or rapidly evolved elements. It also strengthens the capacity to better assess introgression, incomplete lineage sorting (ILS), and structural variation (SV) among bovine species, as well as inferring their effects on the species tree. The assembled genome could be used as a reference in population genomic studies [55] of the gayal. Furthermore, comprehensive comparative analyses of these genomes will improve understanding of the formation and speciation of bovine species.

## Availability of supporting data

The genome sequencing raw reads were deposited in the NCBI SRA database, project ID: PRJNA387130. The assembly and annotation of the gayal genome are available in the *GigaScience* database, *Giga*DB [56]. The complete mtDNA for the gayal generated by Sanger sequencing is also available in GenBank under the ID: MF614103. All supplementary figures and tables are provided in Additional file 1.

## Competing interests

The authors declare that they have no competing interests.

## Authors’ contributions

Y.P.Z., D.D.W., and M.S.W. designed the study. W.W. and Y.D. supervised the analyses. W.H.N., W.T.S., and J.H.W. cultivated the cells. Y.Z. and X.W. performed genome assembly and annotation. M.S.W. extracted genomic DNA and wrote the manuscript with the other authors’ input. M.S.W. and S.Q.Y. sequenced the gayal complete mitochondrial DNA and submitted to GenBank. S.W., Z.J.X., K.X.Q., N.O.O., D.Y., D.D.W., and Y.P.Z. revised the manuscript. All authors read and approved the final manuscript.

## Supplementary Material

GIGA-D-17-00116_Original-Submission.pdf

GIGA-D-17-00116_Revision-1.pdf

Response-to-Reviewer-Comments_Original-Submission.pdf

Reviewer-1-Report-(Original-Submission).pdf

Reviewer-2-Report-(Original-Submission).pdf

Reviewer-2-Report-(Revision-1).pdf

Supplement Figures and Tables
