# Supplementary material for: Draft genome of the gayal, Bos frontalis
Source: Gigascience. 2017 Oct 5;6(11):1–7. doi: 10.1093/gigascience/gix094 (PMC5710521; doi:10.1093/gigascience/gix094)
Supplement: GIGA-D-17-00116_Original-Submission.pdf [file gix094_giga-d-17-00116_original-submission.pdf]

GigaScience

Draft genome of the gayal, Bos frontalis

--Manuscript Draft--

**Manuscript Number:**	GIGA-D-17-00116
**Full Title:**	Draft genome of the gayal, Bos frontalis
**Article Type:**	Data Note
**Funding Information:**	Chinese 973 program (2013CB835200, 2013CB835204) — Dr. Dong-Dong Wu<br>Strategic Priority Research Program of the Chinese Academy of Sciences (XDB13020600) — Dr. Dong-Dong Wu
**Abstract:**	Background:<br>Gayal (Bos frontalis), also known as mithan or mithun, is a large and endangered semi-domesticated bovine that has a limited geographical distribution in the hill-forests of China, Northeast India, Bangladesh, Myanmar, and Bhutan. The chromosome number of the gayal (2n=58) differs from gaur (Bos gaurus, 2n=56) and domesticated cattle (Bos indicus and Bos taurus, 2n=60). Many questions in gayal such as origin, population history as well as genetic basis regarding local adaptation remain largely unresolved. De novo sequencing and assembly of whole gayal genome provides an opportunity to address these issues.<br>Findings:<br>We report a high-depth sequencing, de novo assembly, and annotation of a female gayal genome. Based on Illumina genomic sequencing platform, we have generated 350.38Gb raw data from 16 different insert size libraries. A total of 276.86Gb clean data is retained after quality control. The assembled genome is about 2.85Gb with scaffold and contig N50 sizes of 2.74Mb and 14.41kb, respectively. Repetitive elements account for 48.13% of the genome. Gene annotation has yielded 26,667 protein-coding genes, of which 97.18% have been functionally annotated. BUSCO assessment shows that our assembly captures 93% (3,183 of 4,104) of the core eukaryotic genes, and 83.1% of vertebrate universal single-copy orthologs.<br>Conclusions:<br>We provide a comprehensive de novo genome of the gayal. This genetic resource is integral for inferring the origin of gayal and performing comparative genomic studies to improve understanding of the speciation and divergence of Bovine species.
**Corresponding Author:**	Dong-Dong Wu<br><br>CHINA
**Corresponding Author Secondary Information:**	
**Corresponding Author's Institution:**	
**Corresponding Author's Secondary Institution:**	
**First Author:**	Dong-Dong Wu
**First Author Secondary Information:**	
**Order of Authors:**	Dong-Dong Wu
	Ming-Shan Wang
	Yan Zeng
	Xiao Wang
	Wen-Hui Nie
	Jin-Huan Wang
	Wei-Ting Su

Powered by Editorial Manager® and ProduXion Manager® from Aries Systems Corporation

	Newton O. Otecko
	Zi-Jun Xiong
	Sheng Wang
	Kai-Xing Qu
	Wen Wang
	Yang Dong
	Ya-Ping Zhang
**Order of Authors Secondary Information:**	
**Opposed Reviewers:**	
**Additional Information:**	
**Question**	**Response**
Are you submitting this manuscript to a special series or article collection?	No
**Experimental design and statistics**<br><br>Full details of the experimental design and statistical methods used should be given in the Methods section, as detailed in our Minimum Standards Reporting Checklist. Information essential to interpreting the data presented should be made available in the figure legends.<br><br>Have you included all the information requested in your manuscript?	Yes
**Resources**<br><br>A description of all resources used, including antibodies, cell lines, animals and software tools, with enough information to allow them to be uniquely identified, should be included in the Methods section. Authors are strongly encouraged to cite Research Resource Identifiers (RRIDs) for antibodies, model organisms and tools, where possible.<br><br>Have you included the information requested as detailed in our Minimum Standards Reporting Checklist?	Yes
**Availability of data and materials**<br><br>All datasets and code on which the conclusions of the paper rely must be either included in your submission or deposited in publicly available repositories	Yes

Powered by Editorial Manager® and ProduXion Manager® from Aries Systems Corporation

(where available and ethically appropriate), referencing such data using a unique identifier in the references and in the “Availability of Data and Materials” section of your manuscript.<br><br>Have you have met the above requirement as detailed in our Minimum Standards Reporting Checklist?	

Powered by Editorial Manager® and ProduXion Manager® from Aries Systems Corporation

Manuscript
Click here to download Manuscript Bos.F.ms.170607_revised.docx

Data Note

Draft genome of the gayal, *Bos frontalis*

Ming-Shan Wang [1,2,#], Yan Zeng [1, 2,#], Xiao Wang [1,2, #], Wen-Hui Nie [1], Jin-Huan Wang [1], Wei-Ting Su [1], Newton O. Otecko [1,2], Zi-Jun Xiong [1,4], Sheng Wang [5], Kai-Xing Qu [6], Wen Wang [1, 2], Yang Dong [7,8, *], Dong-Dong Wu [1, 2*], and Ya-Ping Zhang [1, 2, 3, *]

1. State Key Laboratory of Genetic Resources and Evolution, Yunnan Laboratory of Molecular Biology of Domestic Animals, Kunming Institute of Zoology, Chinese Academy of Sciences, Kunming 650223, China
2. Kunming College of Life Science, University of Chinese Academy of Sciences, Kunming 650204, China
3. Laboratory for Conservation and Utilization of Bio-resource, Yunnan University, Kunming 650091, China
4. China National GeneBank, BGI–Shenzhen, Shenzhen 518083, China
5. Laboratory of Animal Genetics, Breeding and Reproduction, Ministry of Agriculture of China, National Engineering Laboratory for Animal Breeding, College of Animal Science and Technology, China Agricultural University, Beijing 100193, China
6. Yunnan Academy of Grassland and Animal Science, Kunming 650212, China
7. Yunnan Agricultural University, Kunming 650100, China
8. Faculty of Life Science and Technology, Kunming University of Science and Technology, Kunming 650500, China

# Contribute equally to this work

* Corresponding author:

Dr. Yang Dong, email: loyalyang@163.com

Dr. Dong-Dong Wu, email: wudongdong@mail.kiz.ac.cn

Dr. Ya-Ping Zhang, email: zhangyp@mail.kiz.ac.cn

Abstract

Background:

Gayal (*Bos frontalis*), also known as mithan or mithun, is a large and endangered semi-domesticated bovine that has a limited geographical distribution in the hill-forests of China, Northeast India, Bangladesh, Myanmar, and Bhutan. The chromosome number of the gayal (2n=58) differs from gaur (*Bos gaurus*, 2n=56) and domesticated cattle (*Bos indicus* and *Bos taurus*, 2n=60). Many questions in gayal such as origin, population history as well as genetic basis regarding local adaptation remain largely unresolved. *De novo* sequencing and assembly of whole gayal genome provides an opportunity to address these issues.

Findings:

We report a high-depth sequencing, *de novo* assembly, and annotation of a female gayal genome. Based on Illumina genomic sequencing platform, we have generated 350.38Gb raw data from 16 different insert size libraries. A total of 276.86Gb clean data is retained after quality control. The assembled genome is about 2.85Gb with scaffold and contig N50 sizes of 2.74Mb and 14.41kb, respectively. Repetitive elements account for 48.13% of the genome. Gene annotation has yielded 26,667 protein-coding genes, of which 97.18% have been functionally annotated. BUSCO assessment shows that our assembly captures 93% (3,183 of 4,104) of the core eukaryotic genes, and 83.1% of vertebrate universal single-copy orthologs.

Conclusions:

We provide a comprehensive *de novo* genome of the gayal. This genetic resource is integral for inferring the origin of gayal and performing comparative genomic studies to improve understanding of the speciation and divergence of Bovine species.

Keywords: *Bos frontalis*; Genome assembly; Annotation; Phylogeny

Data description

Background

The gayal is a large-sized endangered semi-domesticated bovine specie belonging to the family Bovidae, tribe Bovini, group Bovina, genus *Bos* and species *Bos frontalis*. It is also called mithan or mithun, which is distributed spanning east Bhutan through the Arunachal Pradesh in India to the Naga and Chin hills in the Arakan Yomarange region that defines the borders between India, Bangladesh, Myanmar, and China [1, 2]. Gayal has unique characters and appearances compared to gaur, cattle, and other bovine species [3]. These features include a bony dorsal ridge on the shoulder and white stocking on all four legs (**Figure 1**). It has been previously held that gayal was domesticated from gaur and/or from a hybrid descendant from crossing of domestic cattle (*B. indicus* or *B. taurus*) and wild gaur [2, 4, 5]. Karyotype analysis indicates that chromosome number, form, and configuration of gayal (2n=58) are different from gaur (*B. gaurus*, 2n=56) and domesticated cattle (*B. indicus* and *B. taurus*, 2n=60) [2, 5-8]. Phylogenetic analyses in multiple studies based on mtDNA place gayal in conflicting clustering positions with respect to cattle, zebu and wild gaur [5, 9-12]. One of these studies even places gayal as a distinct and separate species/sub-specie [13]. On the other hand, whole genome resequencing indicates that gayal clusters more closely with the common ancestor of cattle and wild yak [5]. This suggests that gayal is likely a hybrid descending from crossing wild male gaur and female domestic cattle. However, these differences illustrate the existence of unresolved uncertainties regarding the origin of gayal. Further complication arises from findings showing that

hybridization of gayal with domestic cattle or gaur may produce fertile female offsprings, unfortunately the males are always infertile [2, 14].

Research has revealed a high genomic divergence among bovine species [15, 16]. Consequently, mapping of resequencing data from one bovine species onto a reference genome of different specie creates avenues for biases and/or errors in sequence alignment and SNP calling procedures. To date, *de novo* genome assemblies for cattle (*Bos taurus*) [17], yak (*Bos grunniens*) [15], wisent (*Bison bonasus*) [18], North American bison (*Bison bison*)[19], zebu (*Bos indicus*) [20], and water buffalo (*Bubalus bubalis*) [21] have been published. This is a critical development towards reducing the challenges inherent in resequencing approaches, and creates great chances to refine the evolutionary history of bovine species. In this study, we report the draft genome assembly of gayal based on the Illumina genome sequencing platform. This valuable resource is an important input to the research of the origin and evolution of this endangered specie.

Sample collection and sequencing

We extracted total genomic DNA from skin fibroblast cell line of a female gayal (NCBI taxonomy ID: 30520, specimen ID: KCB201042, 2n=58) using Qiagen Blood and Tissue Kit (Qiagen, Valencia, CA) according to the manufacturer's instructions. The cells are maintained in the Cell Bank at Kunming Institute of Zoology (**Figure 1**).

A total of 17 paired-end genomic sequence libraries were constructed with a gradient insert size ranging from 180bp to 20kb, and then sequenced on Illumina HiSeq 2000 platform according to the manufacturer's instructions. For short insert size libraries (180bp, 250bp, 450bp, and 600bp), sequencing was performed at the Central Laboratory of Kunming Institute of Zoology with read lengths of 100bp. Sequencing of long insert size libraries (800bp, 2, 5, 10 and 20 kb) was conducted at BGI-Shenzhen with read lengths of 49bp, except for the 800bp insert size library, which were sequenced with a read length of 85bp. A total of 350.38Gb raw sequence data has been generated in our study (Additional file 1: Table S1). Before assembly, we performed strict quality control by removing poor quality reads and/or bases using scripts from SOAPec (version 2.02) [22]. Reads were shortened by 2bp at both head and tail. We dropped any read plus its paired end if it has more than 30 low-quality bases or more than 5% unknown base (usually denoted by N). Reads with duplications and adapters were also removed. We corrected for sequencing errors using the k-mer (13 used in this study) frequency method in SOAPec (version 2.02) [22]. After filtering and correction, we retained 276.86Gb high-quality sequences for genome assembly (**Additional file 1: Table S2**).

***De novo* assembly of gayal genome**

In order to have a basic knowledge about the genome size and attributes of the gayal genome, we performed a 17-mer analysis using clean sequences from 180 and 600bp insert size libraries. We extracted the 17-mer sequences using sliding windows with a

size of 17bp and steps of 1 from the paired-end reads, and calculated the frequency of each 17-mer. Three peaks are observed (at 23X, 45X, and 88X), indicating high heterozygosity. The genome size for gayal is estimated to be 3.7Gb (**Additional file 1: Table S3; Figure 2**).

We then performed *de novo* assembly of gayal genome by Platanus (version 2.0) [23] in three steps: contig construction, scaffolding, and gap filling. To construct contigs based on short insert size libraries (180, 250, 450, 600 and 800bp), we used Platanus (version 2.0) [23], which includes a series of procedures such as constructing de Bruijn graph, clipping tips, merging bubbles, and removing low coverage links. In the scaffolding step, reads from both small and large insert libraries were mapped to contig sequences to construct scaffolds using distance information from read pairs. An additional local assembly of reads, with one end of a read pair uniquely aligned to a contig and the other end located within the gap, was performed using GapCloser (version 1.12) [22]. These processes yielded a final draft gayal genome assembly with a total length of 2.85Gb, contig N50 of 14.4 kb, and scaffold N50 of 2.74Mb (**Table 1**). The assembled genome size is similar to that reported for cattle [24] and yak [15]. To assess the completeness of the assembled gayal genome, we performed BUSCO analysis [25] by searching against the arthropod benchmarking universal single-copy orthologs (BUSCOs, version 2.0). Analyses show that 85.2% and 7.8 % of the 4,104 expected vertebrata genes are identified as complete and partial, respectively. A total of 291 genes are considered missing in our assembly. Of the expected complete vertebrata genes, 3434 and 60 are identified as single copy and duplicated BUSCOS

respectively (**Table 2**). Our newly assembled gayal genome has a slightly lower completeness compared to genomes of yak [15], wisent [18], bison [19], zebu [20], and buffalo [21] (**Table 2**).

Annotation of genomic repeat sequences in gayal genome

To search for the repeated sequences in gayal genome, including tandem repeats (TE), interspersed repeats, and transposable elements (e.g., LINE, SINE, LTR, DNA transposons), we leveraged both *de novo* and homolog-based methods as used in previous publications [26, 27]. For the homolog-based methods, we used RepeatMasker and RepeatProteinMask (http://repeatmasker.org/) to search against the known Repbase TE library (RepBase21.01) [28] and TE protein database, respectively. In the *de novo* method, Piler [29] and RepeatModeler (http://www.repeatmasker.org/) are used to generate a *de novo* gayal repeat library, which is subsequently used by Repeat-Masker to annotate repeats. TRF [30] is then employed to predict tandem repeats. The combined results show that a total of 1.37Gb non-redundant repetitive sequences are identified in the gayal genome, which account for 48.13% of the whole genome. The most predominant elements are the long interspersed nuclear elements (LINEs), which account for 40.43% (1.15Gb in total) of the genome (**Table 3; Additional file 1: Table S4, Figure S1, Figure S2**).

Gayal genome gene structure prediction

For gene structure prediction, we combined both *de novo* and homolog-based

approaches to predict protein-coding genes in the gayal genome. In homolog-based method, gene sets from *Bos taurus* [17], *Canis familiaris* [31], *Homo sapiens* (ENSEMBL 80), *Sus scrofa* [32], *Rattus norvegicus* (ENSEMBL 80), and *Ovis aries* [33] were used as queries to search against gayal genome (**Additional file 1: Table S5**). As for the *de novo* based method, AUGUSTUS [34], Genescan [35], and GlimmerHMM [36] were used as engines to predict gene models. We then merged the gene prediction results derived from both homolog and *de novo* based methods using GLEAN [37] to generate a consensus gene set. In total, we have identified 26,667 protein coding genes with mean of 3.27 exons for each gene (**Table 4; Additional file 1: Figure S3**). The lengths of genes, CDS, introns, and exons in gayal are comparable to the genomes used for homolog-based predictions (**Additional file 1: Figure S3**). In addition, we also predicted the non-coding RNA genes in gayal genome. We used blast to search rRNA against Human rRNA database, and tRNAscan-SE [38] to search tRNA in the genome sequences. We also used blast to search miRNA and snRNA via Rfam (release 11.0) database [39]. In total, our predictions reveal 2,357 ribosomal RNA (rRNA), 29,821 transfer RNA (tRNA), 16,305 microRNAs (miRNA), and 1,380 snRNA genes in the gayal genome (**Additional file 1: Table S5**).

Functional annotation of protein-coding genes

Gene function annotation referrers to searching functional motifs, domains, and possible biological process by aligning translated gene coding sequences to known databases such as SwissProt and TrEMBL [40], NT database (from NCBI), Gene

Ontology (GO), and Kyoto Encyclopaedia of Genes and Genomes (KEGG) [41]. We have annotated all the protein coding genes identified in this study to retrieve functional terms according to InterPro, KEGG, and GO terms. Overall, 81.74% (21,798), 54.56% (14,550), and 66.39% (17,704) genes show enrichment in InterPro, KEGG, and GO respectively. In total, 25,916 protein-coding genes (97.18%) were successfully annotated for conserved functional motifs and functional terms (**Additional file 1: Table S6**).

Phylogenetic analysis and divergence time estimation

To investigate the phylogenic position of gayal, we retrieved nucleotide and protein data for cattle (*Bos taurus*) [17], yak (*Bos grunniens*) [15], wisent (*Bison bonasus*) [18], bison (*Bison bison*) [19], zebu (*Bos indicus*) [20], and buffalo (*Bubalus bubalis*) [21] from the NCBI database. Gene ortholog relationships of gayal and other bovine species were identified by reciprocal blast searching with e-value of 1e-7. Genes with alternative splicing variants were represented by the longest transcript. Multiple sequence alignment of the genes within one copy gene sets were performed using MUSCLE program [42]. Aligned sequences were trimmed to remove potentially unreliably aligned regions and gaps using Gblocks [43]. Alignments with lengths shorter than 100bp were also discarded. Four-fold degenerate sites were extracted and concatenated into a supergene. Modeltest [44] was used to select the best substitution model. MrBayes [45] and RaxML [46] software were used to reconstruct the evolutionary relationships between species, and MEGA5 [47] used to

view the tree. From these analyses, gayal clusters with the common ancestor of cattle and zebu (**Figure 3**). Further, MCMCTREE program, implemented in PAML[48] package, was used to estimate divergence times. The JC69 model and correlated rates molecular clock (clock=3) were used in the calculation. Calibration time for the common ancestor of buffalo and cattle obtained from the TimeTree database (http://www.timetree.org/) was used to calibrate the divergence time estimation. This analysis estimates that gayal diverged from cattle and zebu approximately 5.1 million year ago (**Figure 4**).

In conclusion, we avail a *de novo* assembly of the gayal genome and describe its genetic attributes. Our analyses also demonstrate that together with the genomes of other bovine species, the new gayal genome supports investigations concerning the origin, evolutionary histories, and local adaptation of gayal. This resource is also important for the future conservation of this species. In addition, the *de novo* gayal genome adds to the list of the available bovine genomes, boosting capacity for assessing introgression and incomplete lineage sorting (ILS) among the bovine species, and inferring their effects on the species tree. Future comprehensive comparative analyses of these genomes will improve understanding of the formation and speciation of bovine species.

Availability of supporting data

The genome sequencing raw reads were deposited in the NCBI SRA database, project

ID: PRJNA387130. The assembly and annotation of the gayal genome are also available in the *GigaScience* GigaDB database. All supplementary figures and tables are provided in Additional file 1.

Competing interests

The authors declare that they have no competing interests.

Authors' contributions

YPZ, DDW and MSW designed the study. WW and YD supervised the analyses. WHN, WTS and JHW cultivated the cells. YZ and XW performed genome assembly and annotation. MSW extracted genomic DNA and wrote manuscript with other author's input. SW, ZJX, KXQ, NOO, DY, DDW and YPZ revised the manuscript. All authors read and approved the final manuscript.

Acknowledgements

This work was supported by the Strategic Priority Research Program of the Chinese Academy of Sciences, Grant No. XDB13020600, and the Chinese 973 program (2013CB835200, 2013CB835204). DDW was supported by the Youth Innovation Promotion Association, Chinese Academy of Sciences. N.O.O. is thankful for the support of the CAS-TWAS President's Fellowship Program for Doctoral Candidates.

References

1. Payne WJA, Hodges J: Tropical cattle: origins, breeds and breeding policies. 1997:Blackwell Science.
2. Uzzaman MR, Bhuiyan MS, Edea Z, Kim KS: Semi-domesticated and Irreplaceable Genetic Resource Gayal (Bos frontalis) Needs Effective Genetic Conservation in Bangladesh: A Review. *Asian-Australas J Anim Sci* 2014, 27:1368-1372.
3. Miao YW, Ha F, Gao HS, Yuan F, Li DL, Yuan YY: Polymorphisms of inhibin α gene exon 1 in buffalo (Bubalus bubalis), gayal (Bos frontalis) and yak (Bos grunniens). *Zool Res* 2012, 33:402-408.
4. Payne WJA: *Cattle production in the tropics. Vol. 1. General introduction and breeds and breeding.* London: Longman Group Ltd.; 1970.
5. Mei C, Wang H, Zhu W, Wang H, Cheng G, Qu K, Guang X, Li A, Zhao C, Yang W, et al: Whole-genome sequencing of the endangered bovine species Gayal (Bos frontalis) provides new insights into its genetic features. *Sci Rep* 2016, 6:19787.
6. Shan XN, Chen YF, Luo LH, Cao XM, Song JZ, Zeng YZ: Comparative Studies on the Chromosomes of Five Species of Catties of the Genus Bos in China. *Zool Res* 1980.
7. Adbullah MH, Idris I, Hilmi M: Karyotype of Malayan Gaur (Bos gaurus hubbacki), Sahiwal-Friesian cattle and Gaur x cattle hybrid backcrosses. *Pak J Biol Sci* 2009, 12:896-901.
8. Qu KX, He ZX, Nie WH, Zhang JC, Jin XD, Yang GR, Yuan XP, Huang BZ, Zhang YP, Zan LS: Karyotype analysis of mithun (Bos frontalis) and mithun bull x Brahman cow hybrids. *Genet Mol Res* 2012, 11:131-140.

9. Dorji T, Mannen H, Namikawa T, Inamura T, Kawamoto Y: Diversity and phylogeny of mitochondrial DNA isolated from mithun Bos frontalis located in Bhutan. *Anim Genet* 2010, 41:554-556.

10. Tanaka K, Takizawa T, Murakoshi H, Dorji T, Nyunt MM, Maeda Y, Yamamoto Y, Namikawa T: Molecular phylogeny and diversity of Myanmar and Bhutan mithun based on mtDNA sequences. *Anim Sci J* 2011, 82:52-56.

11. Gou X, Wang Y, Yang S, Deng W, Mao H: Genetic diversity and origin of Gayal and cattle in Yunnan revealed by mtDNA control region and SRY gene sequence variation. *J Anim Breed Genet* 2010, 127:154-160.

12. Li SP, Chang H, Ma GL, Chen HY, Ji DJ, Geng RQ: Molecular phylogeny of the gayal inferred from the analysis of cytochrome b gene entire sequences. *Yi Chuan* 2008, 30:65-70.

13. Baig M, Mitra B, Qu KX, Peng MS, Ahmed I, Miao YW, Zan LS, Zhang YP: Mitochondrial DNA diversity and origin of Bos frontalis. *Current Science* 2013, 104:115-120.

14. Huque KS, Rahman MM, Jalil MA: Study on the Growth Pattern of Gayals (Bos Frontalis) and their Crossbred Calves. *Asian-Australas J Anim Sci* 2001, 14:1245-1249.

15. Qiu Q, Zhang G, Ma T, Qian W, Wang J, Ye Z, Cao C, Hu Q, Kim J, Larkin DM, et al: The yak genome and adaptation to life at high altitude. *Nat Genet* 2012, 44:946-949.

16. Porto-Neto LR, Sonstegard TS, Liu GE, Bickhart DM, Da Silva MV, Machado MA, Utsunomiya YT, Garcia JF, Gondro C, Van Tassell CP: Genomic divergence of zebu and taurine cattle identified through high-density SNP genotyping. *BMC Genomics* 2013,

14:876.

17. Zimin AV, Delcher AL, Florea L, Kelley DR, Schatz MC, Puiu D, Hanrahan F, Pertea G, Van Tassell CP, Sonstegard TS, et al: A whole-genome assembly of the domestic cow, Bos taurus. *Genome Biol* 2009, 10:R42.

18. Wang K, Wang L, Lenstra JA, Jian J, Yang Y, Hu Q, Lai D, Qiu Q, Ma T, Du Z, et al: The genome sequence of the wisent (Bison bonasus). *Gigascience* 2017.

19. Dobson LK. Sequencing the genome of the North American Bison. doctoral dissertation . 2015 (Available electronically from http://hdl.handle.net/1969.1/155759).

20. Canavez FC, Luche DD, Stothard P, Leite KR, Sousa-Canavez JM, Plastow G, Meidanis J, Souza MA, Feijao P, Moore SS, Camara-Lopes LH: Genome sequence and assembly of Bos indicus. *J Hered* 2012, 103:342-348.

21. Temtamy SA, Aglan MS: Brachydactyly. *Orphanet J Rare Dis* 2008, 3:15.

22. Luo R, Liu B, Xie Y, Li Z, Huang W, Yuan J, He G, Chen Y, Pan Q, Liu Y, et al: SOAPdenovo2: an empirically improved memory-efficient short-read de novo assembler. *Gigascience* 2012, 1:18.

23. Kajitani R, Toshimoto K, Noguchi H, Toyoda A, Ogura Y, Okuno M, Yabana M, Harada M, Nagayasu E, Maruyama H, et al: Efficient de novo assembly of highly heterozygous genomes from whole-genome shotgun short reads. *Genome Res* 2014, 24:1384-1395.

24. Bovine Genome S, Analysis C, Elsik CG, Tellam RL, Worley KC, Gibbs RA, Muzny DM, Weinstock GM, Adelson DL, Eichler EE, et al: The genome sequence of taurine cattle: a window to ruminant biology and evolution. *Science* 2009, 324:522-528.

25. Simao FA, Waterhouse RM, Ioannidis P, Kriventseva EV, Zdobnov EM: BUSCO:

assessing genome assembly and annotation completeness with single-copy orthologs. *Bioinformatics* 2015, 31:3210-3212.

26. Wang MS, Li Y, Peng MS, Zhong L, Wang ZJ, Li QY, Tu XL, Dong Y, Zhu CL, Wang L, et al: Genomic Analyses Reveal Potential Independent Adaptation to High Altitude in Tibetan Chickens. *Mol Biol Evol* 2015, 32:1880-1889.

27. Xiong Z, Li F, Li Q, Zhou L, Gamble T, Zheng J, Kui L, Li C, Li S, Yang H, Zhang G: Draft genome of the leopard gecko, Eublepharis macularius. *Gigascience* 2016, 5:47.

28. Kapitonov VV, Jurka J: A universal classification of eukaryotic transposable elements implemented in Repbase. *Nat Rev Genet* 2008, 9:411-412; author reply 414.

29. Edgar RC, Myers EW: PILER: identification and classification of genomic repeats. *Bioinformatics* 2005, 21 Suppl 1:i152-158.

30. Benson G: Tandem repeats finder: a program to analyze DNA sequences. *Nucleic Acids Res* 1999, 27:573-580.

31. Lindblad-Toh K, Wade CM, Mikkelsen TS, Karlsson EK, Jaffe DB, Kamal M, Clamp M, Chang JL, Kulbokas EJ, 3rd, Zody MC, et al: Genome sequence, comparative analysis and haplotype structure of the domestic dog. *Nature* 2005, 438:803-819.

32. Groenen MA, Archibald AL, Uenishi H, Tuggle CK, Takeuchi Y, Rothschild MF, Rogel-Gaillard C, Park C, Milan D, Megens HJ, et al: Analyses of pig genomes provide insight into porcine demography and evolution. *Nature* 2012, 491:393-398.

33. Jiang Y, Xie M, Chen W, Talbot R, Maddox JF, Faraut T, Wu C, Muzny DM, Li Y, Zhang W, et al: The sheep genome illuminates biology of the rumen and lipid metabolism. *Science* 2014, 344:1168-1173.

34. Stanke M, Keller O, Gunduz I, Hayes A, Waack S, Morgenstern B: AUGUSTUS: ab initio prediction of alternative transcripts. *Nucleic Acids Res* 2006, 34:W435-439.

35. Cai Y, Gonzalez JV, Liu Z, Huang T: Computational systems biology methods in molecular biology, chemistry biology, molecular biomedicine, and biopharmacy. *Biomed Res Int* 2014, 2014:746814.

36. Majoros WH, Pertea M, Salzberg SL: TigrScan and GlimmerHMM: two open source ab initio eukaryotic gene-finders. *Bioinformatics* 2004, 20:2878-2879.

37. Elsik CG, Mackey AJ, Reese JT, Milshina NV, Roos DS, Weinstock GM: Creating a honey bee consensus gene set. *Genome Biol* 2007, 8:R13.

38. Lowe TM, Eddy SR: tRNAscan-SE: a program for improved detection of transfer RNA genes in genomic sequence. *Nucleic Acids Res* 1997, 25:955-964.

39. Gardner PP, Daub J, Tate J, Moore BL, Osuch IH, Griffiths-Jones S, Finn RD, Nawrocki EP, Kolbe DL, Eddy SR, Bateman A: Rfam: Wikipedia, clans and the "decimal" release. *Nucleic Acids Res* 2011, 39:D141-145.

40. UniProt C: UniProt: a hub for protein information. *Nucleic Acids Res* 2015, 43:D204-212.

41. Kanehisa M, Goto S, Sato Y, Kawashima M, Furumichi M, Tanabe M: Data, information, knowledge and principle: back to metabolism in KEGG. *Nucleic Acids Res* 2014, 42:D199-205.

42. Edgar RC: MUSCLE: a multiple sequence alignment method with reduced time and space complexity. *BMC Bioinformatics* 2004, 5:113.

43. Talavera G, Castresana J: Improvement of phylogenies after removing divergent and ambiguously aligned blocks from protein sequence alignments. *Syst Biol* 2007,

56:564-577.

44. Posada D: Using MODELTEST and PAUP* to select a model of nucleotide substitution. *Curr Protoc Bioinformatics* 2003, Chapter 6:Unit 6 5.

45. Huelsenbeck JP, Ronquist F: MRBAYES: Bayesian inference of phylogenetic trees. *Bioinformatics* 2001, 17:754-755.

46. Stamatakis A: RAxML-VI-HPC: maximum likelihood-based phylogenetic analyses with thousands of taxa and mixed models. *Bioinformatics* 2006, 22:2688-2690.

47. Tamura K, Peterson D, Peterson N, Stecher G, Nei M, Kumar S: MEGA5: molecular evolutionary genetics analysis using maximum likelihood, evolutionary distance, and maximum parsimony methods. *Mol Biol Evol* 2011, 28:2731-2739.

48. Yang Z: PAML 4: phylogenetic analysis by maximum likelihood. *Mol Biol Evol* 2007, 24:1586-1591.

Figure Legends:

Figure 1. A picture showing a female gayal (*Bos frontalis, provided by Kai-Xing Qu*).

Figure 2. 17-mer frequency distribution of sequencing reads.

Figure 3. Phylogenetic trees of gayal and other bovine species. (A) Tree constructed based on maximum likelihood method, (B) Tree constructed using Bayesian inference.

Figure 4. Divergence time estimated between gayal and other bovine species.

Tables:

Table 1. Statistics of the completeness of the hybrid *de novo* assembly of *Bos frontalis* genome

Terms	Contig		Scaffold	
	**Size**	**number**	**Size**	**number**
**N90**	2,461	211577	158,610	1357
**N80**	5,335	140237	1,060,177	800
**N70**	8,109	99930	1,668,147	587
**N60**	11,044	71764	2,170,469	437
**N50**	14,405	50585	2,737,757	320
**Max length**	208,099		13,764,521	
**Total length**	2,669,378,334		2,848,570,279	
**Total number**		583373		460,059
**Average length**	4575		6,191	
**Number>=500bp**		394757		116481
**Number>=1000bp**		300178		53989
**Number>=2000bp**		229796		19915
**Number>=5000bp**		146493		5387

Table 2. Statistics of the completeness of the assembled genomes for *Bos frontalis* and close related species by BUSCO (version 2)

**Species**	**Terms**	**Complete(C)**	**Complete and single-copy (S)**	**Complete and duplicated (D)**	**Fragmented (F)**	**Missing (M)**
**gayal**	**Number**	3494	3434	60	319	291
	**Proportion**	85.14%	83.67%	1.46%	7.77%	7.09%
**zebu**	**Number**	3698	3644	54	158	248
	**Proportion**	90.11%	88.79%	1.32%	3.85%	6.04%
**wisent**	**Number**	3794	3763	31	180	130
	**Proportion**	92.45%	91.69%	0.76%	4.39%	3.17%
**yak**	**Number**	3841	3809	32	138	125
	**Proportion**	93.59%	92.81%	0.78%	3.36%	3.05%
**buffalo**	**Number**	3817	3780	37	142	145
	**Proportion**	93.01%	92.11%	0.90%	3.46%	3.53%
**bison**	**Number**	3779	3735	44	165	160
	**Proportion**	92.08%	91.01%	1.07%	4.02%	3.90%

Table 3. Statistics of repeats in *Bos frontalis* genome.

Type	Repeat Size （bp）	% of genome
**Trf**	17,696,175	0.62
**Repeatmasker**	868,885,926	30.50
**Proteinmask**	265,003,148	9.30
De novo	917,371,710	32.20
**Total**	1,371,023,312	48.13

Table 4. General statistics of predicted protein-coding genes.

Gene set		Total	Exon number	CDS length (bp)	mRNA length (bp)	Exons per gene	Exon length (bp)	Intron length (bp)
**Homolog**	***Bos taurus***	19,666	141,323	1,325	20,618	7.19	184	3,118
	Canis familiaris	17,627	121,986	1,323	20,802	6.92	191	3,290
	Homo sapiens	24,783	146,172	1,108	17,567	5.89	187	3,360
	Sus scrofa	20,283	121,282	1,142	16,288	5.97	191	3,041
	Rattus norvegicus	17,988	117,965	1,277	19,469	6.55	194	3,273
	Ovis aries	20,947	147,367	1,287	20,973	7.03	183	3,261
De novo	**AUGUSTUS**	41,227	180,664	1,127	22,786	4.38	257	6,403
	**GlimmerHMM**	27,067	104,294	874	5,433	3.85	226	1,597
	**Genescan**	46,598	297,828	1,321	36,828	6.39	206	6,585
**Glean**		26,667	87,392	1,156	4,996	3.27	352	1,686

Figure1

Click here to download Figure Figure1.pdf

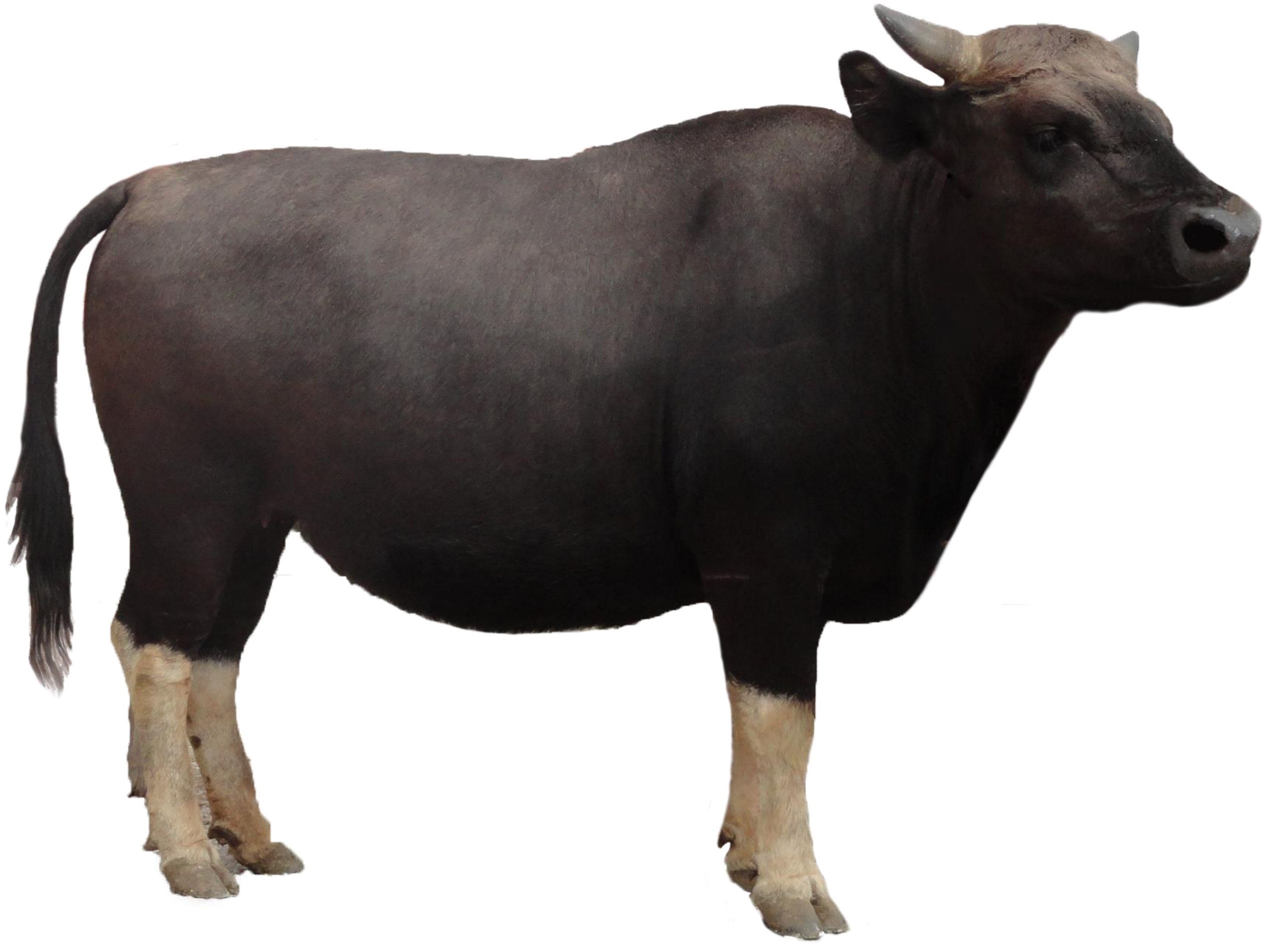

Figure 2

Click here to download Figure Figure2.pdf

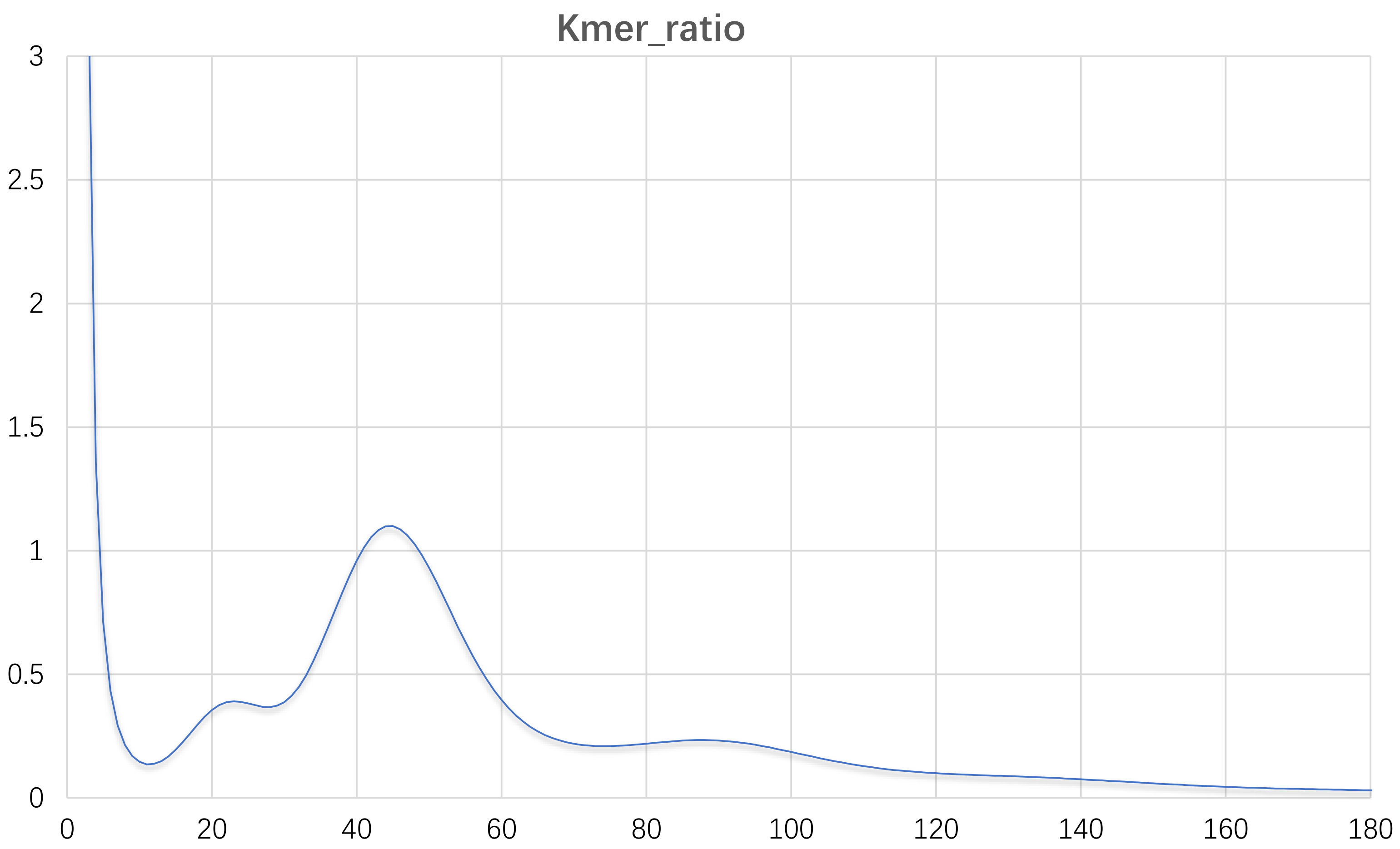

Figure3

Click here to download Figure Figure3.pdf

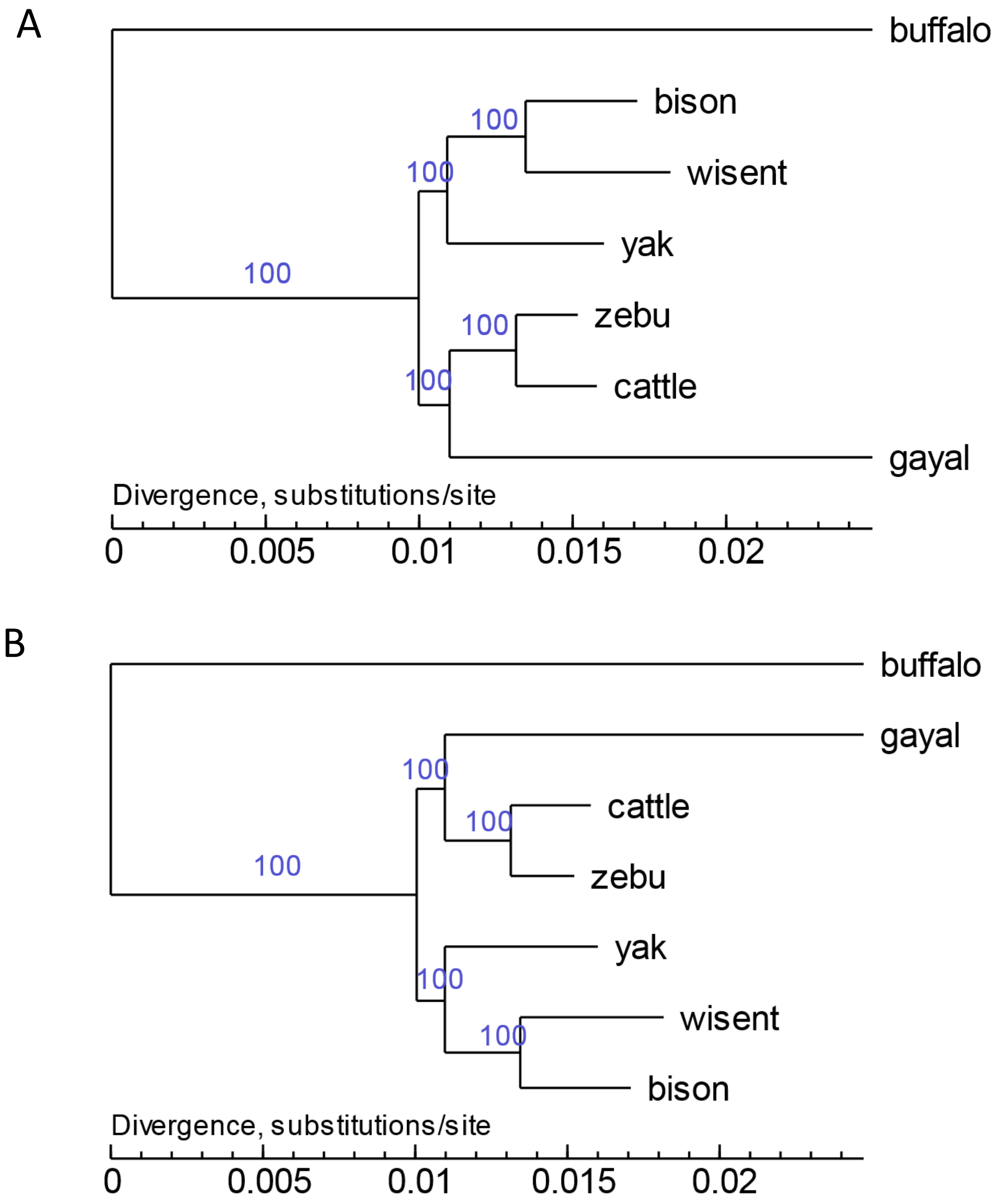

Figure4

Click here to download Figure Figure4.pdf

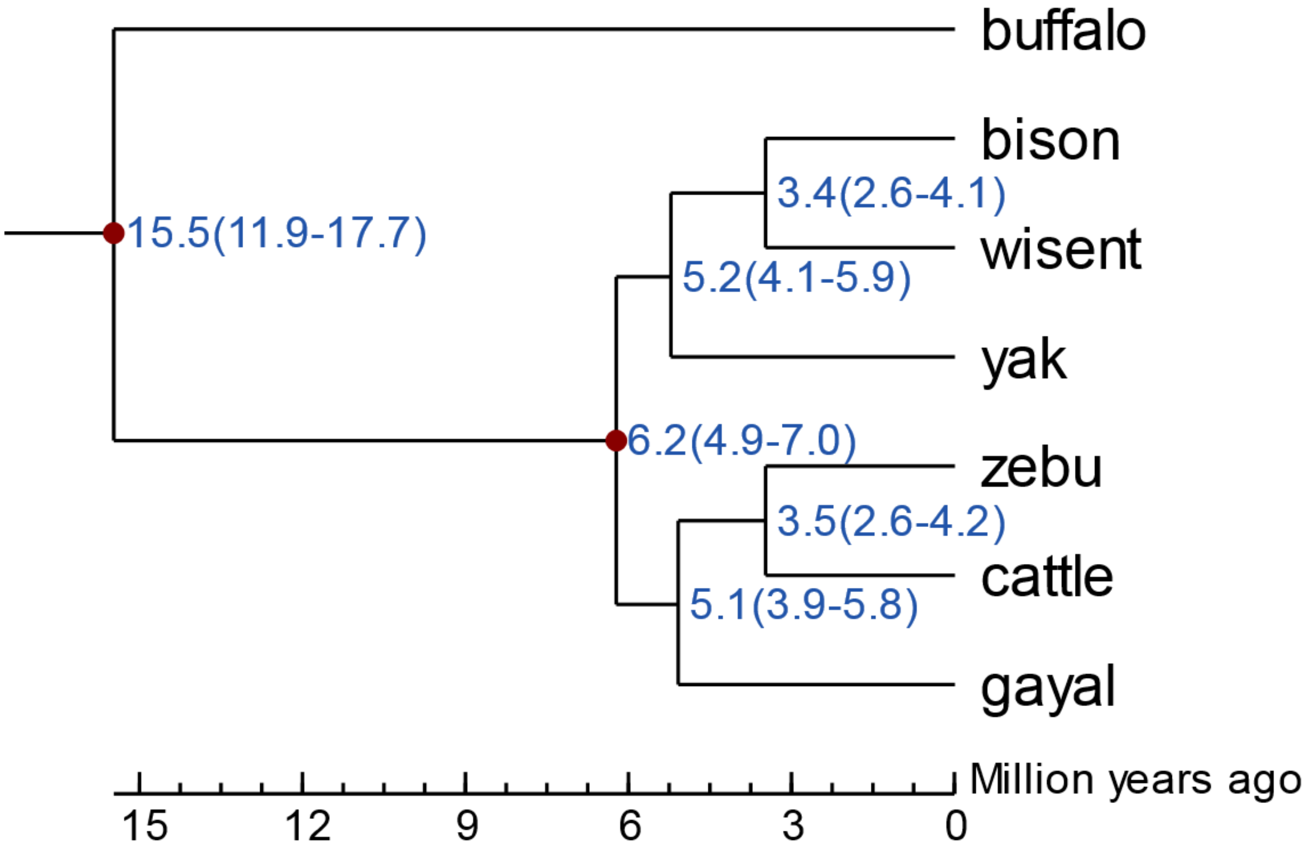

Click here to access/download
Supplementary Material
SupplementaryInfor.doc
