# Supplementary material for: Draft genome of the gayal, Bos frontalis
Source: Gigascience. 2017 Oct 5;6(11):1–7. doi: 10.1093/gigascience/gix094 (PMC5710521; doi:10.1093/gigascience/gix094)
Supplement: GIGA-D-17-00116_Revision-1.pdf [file gix094_giga-d-17-00116_revision-1.pdf]

# GigaScience

## Draft genome of the gayal, Bos frontalis

--Manuscript Draft--

| | |
|---|---|
| **Manuscript Number:** | GIGA-D-17-00116R1 |
| **Full Title:** | Draft genome of the gayal, Bos frontalis |
| **Article Type:** | Data Note |
| **Funding Information:** | Chinese 973 program (2013CB835200, 2013CB835204) — Dr. Dong-Dong Wu<br>Strategic Priority Research Program of the Chinese Academy of Sciences (XDB13020600) — Dr. Dong-Dong Wu |
| **Abstract:** | Background:<br>Gayal (Bos frontalis), also known as mithan or mithun, is a large endangered semi-domesticated bovine that has a limited geographical distribution in the hill-forests of China, Northeast India, Bangladesh, Myanmar, and Bhutan. Many questions about the gayal such as its origin, population history as well as genetic basis of local adaptation remain largely unresolved. De novo sequencing and assembly of the whole gayal genome provides an opportunity to address these issues.<br><br>Findings:<br>We report a high-depth sequencing, de novo assembly, and annotation of a female Chinese gayal genome. Based on the Illumina genomic sequencing platform, we have generated 350.38Gb raw data from 16 different insert-size libraries. A total of 276.86Gb clean data is retained after quality control. The assembled genome is about 2.85Gb with scaffold and contig N50 sizes of 2.74Mb and 14.41kb, respectively. Repetitive elements account for 48.13% of the genome. Gene annotation has yielded 26,667 protein-coding genes, of which 97.18% have been functionally annotated. BUSCO assessment shows that our assembly captures 93% (3,183 of 4,104) of the core eukaryotic genes, and 83.1% of vertebrate universal single-copy orthologs.<br><br>Conclusions:<br>We provide the first comprehensive de novo genome of the gayal. This genetic resource is integral for investigating the origin of the gayal and performing comparative genomic studies to improve understanding of the speciation and divergence of Bovine species. The assembled genome could be used as reference in future population genetic studies of gayal. |
| **Corresponding Author:** | Dong-Dong Wu<br><br>CHINA |
| **Corresponding Author Secondary Information:** | |
| **Corresponding Author's Institution:** | |
| **Corresponding Author's Secondary Institution:** | |
| **First Author:** | Dong-Dong Wu |
| **First Author Secondary Information:** | |
| **Order of Authors:** | Dong-Dong Wu |
| | Ming-Shan Wang |
| | Yan Zeng |
| | Xiao Wang |
| | Wen-Hui Nie |
| | Jin-Huan Wang |

*Powered by Editorial Manager® and ProduXion Manager® from Aries Systems Corporation*

| | |
|---|---|
| | Wei-Ting Su |
| | Newton O. Otecko |
| | Zi-Jun Xiong |
| | Sheng Wang |
| | Kai-Xing Qu |
| | Shou-Qing Yan |
| | Min-Min Yang |
| | Wen Wang |
| | Yang Dong |
| | Ya-Ping Zhang |
| **Order of Authors Secondary Information:** | |
| **Response to Reviewers:** | Response to editor:<br>Your manuscript "Draft genome of the gayal, Bos frontalis" (GIGA-D-17-00116) has been assessed by our reviewers. Although it is of interest, we are unable to consider it for publication in its current form. The reviewers have raised a number of points which we believe would improve the manuscript and may allow a revised version to be published in GigaScience. Their reports, together with any other comments, are below. In particular, the reviewers point out that the previous literature in this field must be referenced more completely and accurately. Given the previous, published work on genome sequencing of this species, you should also explain better what the novel contribution of your study is.<br>Reply:<br>Thanks the editor for handling our manuscript and comments. We found the comments and suggestions very helpful. We have revise the manuscripts carefully, improving literature review and referencing, as wells as clarifying sample origin and novelty of our research. We believe the revisions have greatly improved our manuscript for publication in your reputable journal.<br><br>Please pay particular attention to point 7) of reviewer 2 regarding the inferred genome size - this may need careful re-assessment.<br>Reply:<br>Thanks for the comment. In the previous estimation, we used raw sequencing reads (without filtration) to infer the K-mer frequency and genome size. We have corrected this mistake and re-assessed the genome size using only the clean reads that passed quality filtration in the genome assembly. The newly estimated genome size is 3.15Gb, still slightly larger than what we assembled (2.85Gb). We have also illustrated and discussed in our responses the discrepancies that commonly occur between K-mer estimated and assembled genome sizes. Please full details in the response to point 4) of reviewer 1 and point 7) of reviewer 2.<br><br>Please also provide more details regarding the origin of the sample, and address all other points of the reviewers.<br>Reply:<br>We have provided more details on sample origin. The gayal used in this study originated from Dulong, a city in Yunnan province, China. It is currently reared in Yunnan Academy of Grassland and Animal Science for breeding and research purposes. Karyotype examination showed it has 2n=58 chromosomes (see figure2). We have addressed all the points by the reviewers in the one by one response below.<br><br>Response to reviewers:<br>Reviewer #1:<br>1. Average exon in text is 3.27 where as in corresponding table it is 7.19<br>Reply:<br>We are very sorry for the mistake. We predicted genes using both homolog and de novo based methods. Both genes set were subsequently merged using glean to produce the final gene set, in which average exons per gene is 3.27. In the homolog method, using Bos taurus as closed species to search again gayal genome, we |

Powered by Editorial Manager® and ProduXion Manager® from Aries Systems Corporation

predicted 19,666 protein coding genes with average exons of 7.19 per gene. We have made appropriate revisions for consistency and clarity.

2. Reference for buffalo assembly is missing from references
Reply:
We are sorry for this oversight; we have added the reference accordingly.

3. References need to be rechecked as per text
Reply:
Thanks to the reviewer for the comment. We have carefully revised the references one by one.

4. There is a need to re-look into figure of 3.7gb as the genome size of Mithun
Reply:
Thanks for the comment. In the previous estimation, we used raw sequencing reads (without filtration) to infer the K-mer frequency and genome size. We have corrected this mistake and re-assessed the genome size using only the clean reads that passed quality filtration in the genome assembly. The newly estimated genome size is 3.15Gb, still slightly larger than what we assembled (2.85Gb). However, minimal discrepancies between K-mer estimated and assembled genome sizes is a common occurrence in NGS studies (Yim et al. Nat Genet. 2014;46(1):88-92; Wang et al. Gigascience. 2017;doi: 10.1093/gigascience/gix016; Fan et al. Nat Commun. 2013;4:1426; Gao et al. Gigascience. 2017; doi:10.1093/gigascience/gix041). We think low sequencing bases likely lead to over estimation of genome size. In addition, as demonstrated by the previous gayal sequencing (Mei et al.2016) and our current work, there is high heterozygosity in the gayal genome, which also likely influence its genome size estimation.

Reviewer #2:
This is a well-written account of a whole-genome sequence of the gayal, a most interesting bovine species. However, it should become clear what is the novelty of the results relative to an earlier report on a WGS of the same species. Furthermore, more details about the sample origin should be given, while referencing to the literature about the gayal is superficial and even incorrect. We recommend a major revision.
Reply:
We thank the reviewer very much for the constructive analysis and comments on our manuscript. We have followed the suggestions of the reviewer to revise our manuscript, particularly discussing the novelty of the results relative previous research on gayal and other bovine relatives, explaining sample origin, as well as revising the literature review and references. Please see below a detailed point by point response to the detailed comments.

Detailed comments
1. As cited, Mei et al. (2016) already published a gayal WGS, so a separate publication on another sequence should be justified, for instance because of a better coverage, contig and scaffold statistics and gene coverage.
Reply:
Thanks the reviewer for the comment. As stated by the reviewer, last year, Mei et al. published a study in which they re-sequenced gayal WGS. They generated 36.3Gb genome sequence data with an average sequencing depth of 13.06X after mapping the sequencing reads to cattle reference genome. Their analysis was therefore based on SNPs obtained by mapping gayal genome to cow reference genome. They further constructed phylogenic trees using a subset of only 20 randomly selected single ortholog copy genes in Bos taurus, Bos mutus (wild yak) and Bubalus bubalis genomes, placing gayal off B. mutus and B. taurus. While we appreciate the importance of their work and other preceding partial genome research on gayal, we also take note that they used a resequencing approach for species that does not have a reference genome, forcing them to map the gayal sequencing reads to a cattle genome. Their study, as well as our own analysis, shows that gayal has a high heterozygosity and is far divergent from cattle. Hence, using cattle reference when mapping gayal sequencing reads is definitely likely to produce biases during alignment and SNP calling procedures. In addition, they did not determine/report the karyotype of the gayal they used. This is an important matter for ongoing research on gayal as

Powered by Editorial Manager® and ProduXion Manager® from Aries Systems Corporation

gayal hybrids are common in China. In our study, we have tried to take care of these limitations. We used a female gayal with 58 chromosomes to perform high coverage whole genome sequencing (350.38Gb raw data) with libraries constructed based on different insert sizes, and then performed de novo assembly. Besides the detailed analysis and description of the genome properties, we also state the karyotype of the gayal used and its phylogenetic relationship with other bovines (validated by complete mtDNA gayal sequences generated by Sanger sequencing method). Overall, our study represents the pioneer de novo assembly of the gayal whole genome, and Sanger sequencing of its complete mtDNA. Our study therefore presents a suitable reference genome for future studies on gayal, plus other important resources and insights that will facilitate research on gayal and other bovine species.

We have concisely included these descriptions in the revised manuscript.

2. The geographic origin sample of the sample should be specified. Chinese gayals, or Dulong cattle, are known to harbor zebu or taurine mtDNA (Gou et al. 2010, J.Anim.Breeding Genet. 127, 154-160; Mei et al. 2016) and may very well differ from individuals with an Indian origin.
Reply:
Thanks the reviewer for the comment. We have provided more details on sample origin. The gayal used in this study originated from Dulong, a city in Yunnan province, China. It is currently reared in Yunnan Academy of Grassland and Animal Science for breeding and research purposes. As suggested, we have explained the sample origin more clearly and cited these references appropriately in our revised manuscript.

3. For this reason the mtDNA sequence should be retrieved and compared to the several available gayal mtDNA sequences published previously.
Reply:
As suggested, we searched NCBI-Nucleotide database for published mtDNA sequences gayal. Unfortunately, there is no complete mtDNA assembly available for gayal, except partial mtDNA sequences like D-loop, cytb, and 16s. Considering the lower ability of NGS to accurately recover duplicated sequences that characterize regions like the D-loop in mtDNA, we sequenced complete mtDNA from the gayal in our study using Sanger method. We then downloaded sequences of mtDNA for gayal and other Bovine species, and constructed phylogenic trees. Bellow are trees constructed using maximum likelihood method based on complete mtDNA (see figure 5) and cytb (see figure S4) sequences. We observed that the gayal in our study clustered with gaurs and gayal from Dulong, Myanmar, Bhutan, and Manipuri. We have submitted the new complete mtDNA sequence to the Genbank and added this analysis in our revised manuscript.

4. Thai and Malaysian gaur have indeed a 2n=56 karyotype, but Indian gaur, which occurs in the geographic area overlapping with the range of the gayals, has 2n=58 (Winter et al., 1984, Res Vet Sci 36: 276-283; Gallagher et al., 1992, J Hered 83:287-298; Mastromonaco et al., 2004, Chromosome Res. 2:725-31).
Reply:
We thanks the reviewer for this comment. We agree with the reviewer that determining and reporting the karyotype of gayal is important due to these cryptic variations. Besides reporting the karyotype of the gayal in our study, we have revised our manuscript to reflect the insights offered by the reviewer plus the appropriate citations.

5. The cited references (5,14) do not show that gaur x gayal male offspring are sterile. Although I could not find literature about the outcome of this hybrid cross, it is generally assumed that gayal is the domestic form of the gayal, also because they have similar mtDNA and Y-chromosomal DNA sequences (Hassanin et al. 2012, C.R.Biologies 335:32-50; Nijman et al. 2008, Cladistics 24:723-726).
Reply:
We are sorry for the oversight. We have revised this description to maintain only the details that have a solid literature backing. Thanks for the comment.

6. The URL reference [19] of the academic thesis describing the American bison WGS is still inaccessible. I guess that this WGS has been downloaded from Genbank, which should be made clear.
Reply:

Powered by Editorial Manager® and ProduXion Manager® from Aries Systems Corporation

| | |
|---|---|
| | Thanks to the reviewer for the comment. We download the sequence from Genbank and have revised the citation appropriately.<br><br>7. The inferred genome size for the gayal of 3.7 Gbp, larger than the genome of any related mammalian species, is not believable and not consistent with the gene coverage.<br>Reply:<br>We thank the reviewer for this important observation. In the previous estimation, we used raw sequencing reads (without filtration) to infer the K-mer frequency and genome size. We have corrected this mistake and re-assessed the genome size using only the clean reads that passed quality filtration in the genome assembly. The newly estimated genome size is 3.15Gb, still slightly larger than what we assembled (2.85Gb). However, minimal discrepancies between K-mer estimated and assembled genome sizes is a common occurrence in NGS studies (Yim et al. Nat Genet. 2014;46(1):88-92; Wang et al. Gigascience. 2017;doi: 10.1093/gigascience/gix016; Fan et al. Nat Commun. 2013;4:1426; Gao et al. Gigascience. 2017; doi:10.1093/gigascience/gix041). We think that low quality sequencing bases likely lead to over estimation of genome size. In addition, as demonstrated by the previous gayal sequencing (Mei et al.2016) and our current work, there is high heterozygosity in the gayal genome, which also likely influence its genome size estimation.<br><br>8. It may be interesting to compare the recovered DNA repeats with those from the bovine WGS.<br>Reply:<br>Thanks to the reviewer for the comment. It is an interesting topic to compare the repeats in different bovine species. However, whole genome solely based on NGS has low efficiency to assemble repeat sequences (Wang et al.2016. Nature Genetics 48(9): 972-3). In addition, many of these bovine genomes are generated without uniform sequencing platform and assembly strategies. Further, the repeats predictions do not follow a harmonized pipeline, hence remain just draft genomes. It is difficult to distinguish the lose or increase of repeats in one species to be attributable to evolution or from technique/sequencing effects. The main reach of the current study is providing a comprehensive genetic resources and a draft reference genome for gayal to facilitate future research. We believe that in future, when high quality genomes for the bovine species become available, it will be fascinating to retrieve and compare DNA repeats evolution among the bovine species.<br><br>9. page 9 last line: vertebrata > vertebrate.<br>Reply:<br>Thanks, we have revised this accordingly. |
| **Additional Information:** | |
| **Question** | **Response** |
| Are you submitting this manuscript to a special series or article collection? | No |
| **Experimental design and statistics**<br><br>Full details of the experimental design and statistical methods used should be given in the Methods section, as detailed in our Minimum Standards Reporting Checklist. Information essential to interpreting the data presented should be made available in the figure legends.<br><br>Have you included all the information requested in your manuscript? | Yes |
| **Resources** | Yes |

| | |
|---|---|
| A description of all resources used, including antibodies, cell lines, animals and software tools, with enough information to allow them to be uniquely identified, should be included in the Methods section. Authors are strongly encouraged to cite Research Resource Identifiers (RRIDs) for antibodies, model organisms and tools, where possible.<br><br>Have you included the information requested as detailed in our Minimum Standards Reporting Checklist? | |
| **Availability of data and materials**<br><br>All datasets and code on which the conclusions of the paper rely must be either included in your submission or deposited in publicly available repositories (where available and ethically appropriate), referencing such data using a unique identifier in the references and in the "Availability of Data and Materials" section of your manuscript.<br><br>Have you have met the above requirement as detailed in our Minimum Standards Reporting Checklist? | Yes |

Manuscript
Click here to download Manuscript Bos.F.ms.170804_revised.docx

# Data Note

## Draft genome of the gayal, *Bos frontalis*

Ming-Shan Wang [1,2,#], Yan Zeng [1, 2,#], Xiao Wang [1,2, #], Wen-Hui Nie [1], Jin-Huan Wang [1], Wei-Ting Su [1], Newton O. Otecko [1,2], Zi-Jun Xiong [1,4], Sheng Wang [5], Kai-Xing Qu [6], Shou-Qing Yan [7], Min-Min Yang [1,2], Wen Wang [1, 2], Yang Dong [8,9, *], Dong-Dong Wu [1, 2*], and Ya-Ping Zhang [1, 2, 3, *]

1. State Key Laboratory of Genetic Resources and Evolution, Yunnan Laboratory of Molecular Biology of Domestic Animals, Kunming Institute of Zoology, Chinese Academy of Sciences, Kunming 650223, China
2. Kunming College of Life Science, University of Chinese Academy of Sciences, Kunming 650204, China
3. Laboratory for Conservation and Utilization of Bio-resource, Yunnan University, Kunming 650091, China
4. China National GeneBank, BGI–Shenzhen, Shenzhen 518083, China
5. Laboratory of Animal Genetics, Breeding and Reproduction, Ministry of Agricult ure of China, National Engineering Laboratory for Animal Breeding, College of Animal Science and Technology, China Agricultural University, Beijing 100193, China
6. Yunnan Academy of Grassland and Animal Science, Kunming 650212, China
7. College of Animal Science, Jilin University, Changchun 130062, China
8. Yunnan Agricultural University, Kunming 650100, China
9. Faculty of Life Science and Technology, Kunming University of Science and Technology, Kunming 650500, China

# Contribute equally to this work

* Corresponding author:

Dr. Yang Dong, email: loyalyang@163.com

Dr. Dong-Dong Wu, email: wudongdong@mail.kiz.ac.cn

Dr. Ya-Ping Zhang, email: zhangyp@mail.kiz.ac.cn

ORCID details:

Newton O. Otecko: 0000-0002-9149-4776

**Abstract**

**Background:**

Gayal (*Bos frontalis*), also known as mithan or mithun, is a large endangered semi-domesticated bovine that has a limited geographical distribution in the hill-forests of China, Northeast India, Bangladesh, Myanmar, and Bhutan. Many questions about the gayal such as its origin, population history as well as genetic basis of local adaptation remain largely unresolved. *De novo* sequencing and assembly of the whole gayal genome provides an opportunity to address these issues.

**Findings:**

We report a high-depth sequencing, *de novo* assembly, and annotation of a female Chinese gayal genome. Based on the Illumina genomic sequencing platform, we have generated 350.38Gb raw data from 16 different insert-size libraries. A total of 276.86Gb clean data is retained after quality control. The assembled genome is about 2.85Gb with scaffold and contig N50 sizes of 2.74Mb and 14.41kb, respectively. Repetitive elements account for 48.13% of the genome. Gene annotation has yielded 26,667 protein-coding genes, of which 97.18% have been functionally annotated. BUSCO assessment shows that our assembly captures 93% (3,183 of 4,104) of the core eukaryotic genes, and 83.1% of vertebrate universal single-copy orthologs.

**Conclusions:**

We provide the first comprehensive *de novo* genome of the gayal. This genetic resource is integral for investigating the origin of the gayal and performing comparative genomic studies to improve understanding of the speciation and divergence of Bovine species. The assembled genome could be used as reference in future population genetic studies of gayal.

**Keywords:** *Bos frontalis*; Genome assembly; Annotation; Phylogeny

## Data description

## Background

The gayal is a large-sized endangered semi-domesticated bovine species belonging to the family Bovidae, tribe Bovini, group Bovina, genus *Bos* and species *Bos frontalis* (NCBI Taxon ID: 30520). It is also called the mithan or mithun. Its distribution spans eastern Bhutan through the Arunachal Pradesh in India to the Naga and Chin hills in the Arakan Yomarange region that defines the borders between India, Bangladesh, Myanmar, and China [1, 2]. The Gayal has unique characters and appearances compared to gaur, cattle, and other bovine species [3]. These features include a bony dorsal ridge on the shoulder and white stockings on all four legs (**Figure 1**). It has been previously held that gayal was domesticated from gaur and/or from a hybrid descendant from crossing domestic cattle (*B. indicus* or *B. taurus*) and wild gaur [2, 4, 5]. Karyotype analysis indicates that Indian gayal has a 2n=58 karyotype, same as the local gaur (2n=58) [6, 7], but different from Chinese and Malaysian gaurs (*B. gaurus*, 2n=56) as well as domesticated cattle (*B. indicus* and *B. taurus*, 2n=60) [2, 6-10]. Phylogenetic analyses in multiple studies based on mtDNA or Y-chromosomal DNA place gayal in conflicting clustering positions with respect to cattle, zebu and wild gaur. For example, Chinese gayal, or Dulong cattle, are known to harbor zebu or taurine mtDNA footprints, suggesting hybrid origin [5, 11]; and more studies have shown a high mtDNA and Y-chromosomal DNA sequences similarity between gayal and guar [12-15]. One study has even placed the gayal as a distinct and separate species/sub-species [16]. In contrast, phylogenetic analyses based on SNPs from 20

randomly selected single copy gene orthologs of *B. taurus*, *B. mutus* (wild yak) and *Bubalus bubalis* placed Chinese gayal off the *B. mutus* and *B. taurus* clade, indicating that gayal is distinct from the modern domestic cattle, *B. taurus* [5]. These authors further demonstrated from mtDNA analysis that the gayal is the most proximal to domesticated cattle (*B. taurus* and *B. indicus*), suggesting that the gayal could be a hybrid emanating from crossing of male wild gaur and female domestic cattle [5]. These differences illustrate the existence of unresolved uncertainties regarding the origin of gayal.

Research has revealed a high genomic divergence among bovine species [17, 18]. Consequently, mapping of resequencing data from one bovine species onto the reference genome of different species (for instance, gayal versus cattle) creates avenues for biases and/or errors in sequence alignment and SNP calling procedures. This challenge extends to species of great research interest like gayal, which so far have no *de novo* assembled reference genome. For instance, Mei et al. recently reported a whole genome sequencing (resequencing) of Chinese gayal [5]. In their analysis, they retrieved variants based on mapping gayal sequencing reads (13.06X) to the cattle reference genome. Importantly, hydride gayals are hard to distinguish only through morphological characterization, yet Mei *et al.* did not examine the karyotype of the gayal they resequenced. In contrast to the gayal, *de novo* genome assembly has been accomplished for related species like cattle (*Bos taurus*) [19], yak

(*Bos grunniens*) [17], wisent (*Bison bonasus*) [20], North American bison (*Bison bison*) [21], zebu (*Bos indicus*) [22], and water buffalo (*Bubalus bubalis*) [23]. This represents a critical resource towards mitigating the challenges inherent in resequencing approaches, and provides great opportunities to refine the evolutionary history of bovine species. In this study, we for the first time report the draft genome assembly of the gayal with a high sequencing depth generated on the Illumina genome sequencing platform. This valuable resource is an important input to the research of the origin and evolution of this species that has been classified as an endangered by the IUCN.

**Sample collection and sequencing**

The gayal (NCBI taxonomy ID: 30520) used for genome sequencing came from a Dulong in Yunnan province, China (**Figure 1**). It was kept at Yunnan Academy of Grassland and Animal Science for breeding and research purposes. Karyotype examination showed that it has 2n=58 chromosomes (**Figure 2**). We extracted total genomic DNA from skin fibroblast cell lines of the gayal using Qiagen Blood and Tissue Kit (Qiagen, Valencia, CA) according to the manufacturer's instructions. The cells are maintained at the Cell Bank of Kunming Institute of Zoology (specimen ID: KCB201042). A total of 17 paired-end genomic sequence libraries were constructed with a gradient insert size ranging from 180bp to 20kb, and sequencing was carried out on the Illumina HiSeq 2000 platform according to the manufacturer’s instructions.

For short insert size libraries (180bp, 250bp, 450 bp, and 600bp), sequencing was performed at the Central Laboratory of Kunming Institute of Zoology with read lengths of 100bp. Sequencing of long insert size libraries (800 bp, 2, 5, 10 and 20 kb) was conducted at BGI-Shenzhen with read lengths of 49bp, except for the 800bp insert size library which were sequenced with a read length of 85bp. A total of 350.38Gb raw sequence data has been generated in our study (**Additional file 1: Table S1**). Before assembly, we performed strict quality control by removing poor quality reads and/or bases using scripts from SOAPec (version 2.02) [24]. Reads were shortened by 2bp at both head and tail. We dropped any read plus their corresponding paired-end if it contained more than 30 low-quality bases or more than 5% unknown base (usually denoted by N). Reads with duplications and adapters were also removed. We corrected for sequencing errors using the k-mer (13 used in this study) frequency method in SOAPec (version 2.02) [24]. After filtering and correction, we retained 276.86Gb high-quality sequences for genome assembly (**Additional file 1: Table S2**).

***De novo* assembly of gayal genome**

In order to have a basic knowledge about the genome size and attributes of the gayal genome, we performed a 17-mer analysis using clean and high quality sequences from 180 and 450bp insert size libraries. We extracted the 17-mer sequences using sliding windows with a size of 17bp, and calculated the frequency of each 17-mer. A clear peak at 25X with two upward convex signals besides it is evident, suggesting high heterozygosity. The genome size for gayal is estimated to be 3.15Gb (**Additional file 1: Table S3; Figure 3**).

We then performed *de novo* assembly of gayal genome using Platanus (version 2.0) (Platanus, RRID:SCR_015531) [25] in three steps: contig construction, scaffolding, and gap filling. To construct contigs based on short insert size libraries (180, 250, 450, 600 and 800bp), we used Platanus (version 2.0) [25], which includes a series of procedures such as constructing de Bruijn graphs, clipping tips, merging bubbles, and removing low coverage links. In the scaffolding step, reads from both small and large insert libraries were mapped to contig sequences to construct scaffolds using distance information from read pairs. An additional local assembly of reads, with one end of a read pair uniquely aligned to a contig and the other end located within the gap, was performed using GapCloser (version 1.12) (GapCloser , RRID:SCR_015026) [24]. These processes yielded a final draft gayal genome assembly with a total length of 2.85Gb, contig N50 of 14.4 kb, and scaffold N50 of 2.74Mb (**Table 1**). The assembled genome size is similar to that reported for cattle [26] and yak [17]. To assess the completeness of the assembled gayal genome, we performed BUSCO analysis (BUSCO , RRID:SCR_015008) [27] by searching against the arthropod universal benchmarking single-copy orthologs (BUSCOs, version 2.0). Overall, 85.2% and 7.8 % of the 4,104 expected vertebrate genes are identified in the assembled genome as complete and partial, respectively. Approximately 291 genes could be considered missing in our assembly. Of the expected complete vertebrate genes, 3434 and 60 are identified as single copy and duplicated BUSCOs, respectively (**Table 2**). Our newly assembled gayal genome has a slightly lower completeness rate compared to genomes of yak [17], wisent [20],

bison [21], zebu [22], and buffalo [23] (**Table 2**).

**Annotation of genomic repeat sequences in gayal genome**

To search for the repeated sequences in gayal genome, including tandem repeats, interspersed repeats, and transposable elements (TE) (e.g. LINE, SINE, LTR, DNA transposons), we leveraged both *de novo* and homolog-based methods as used in previous publications [28, 29]. For the homolog-based methods, we used RepeatMasker (RepeatMasker , RRID:SCR_012954) and RepeatProteinMask (http://repeatmasker.org/) to search against the known Repbase TE library (RepBase21.01) [30] and TE protein database, respectively. In the *de novo* method, Piler [31] and RepeatModeler (RepeatModeler , RRID:SCR_015027) (http://www.repeatmasker.org/) are used to generate a *de novo* gayal repeat library, which is subsequently used in Repeat-Masker to annotate repeats. TRF [32] is then employed to predict tandem repeats. The combined results show that a total of 1.37Gb non-redundant repetitive sequences are identified in the gayal genome, which account for 48.13% of the whole genome. The most predominant repeat is the long interspersed nuclear elements (LINEs), which account for 40.43% (1.15Gb in total) of the genome (**Table 3; Additional file 1: Table S4, Figure S1, Figure S2**).

**Gayal genome gene structure prediction**

For gene structure prediction, we combined both *de novo* and homolog-based approaches to predict protein-coding genes in the gayal genome. In homolog-based

method, gene sets from *Bos taurus* [19], *Canis familiaris* [33], *Homo sapiens* (ENSEMBL 80), *Sus scrofa* [34], *Rattus norvegicus* (ENSEMBL 80), and *Ovis aries* [35] were used as queries to search against gayal genome (**Additional file 1: Table S5**). For the *de novo* based method, AUGUSTUS (Augustus: Gene Prediction , RRID:SCR_008417) [36], Genescan (GENSCAN , RRID:SCR_012902) [37], and GlimmerHMM (GlimmerHMM , RRID:SCR_002654) [38] were used as engines to predict gene models. We then merged the gene prediction results derived from both methods using GLEAN [39] to generate a consensus gene set. In total, we have identified 26,667 protein coding genes with a mean of 3.27 exons per gene (**Table 4; Additional file 1: Figure S3**). The lengths of genes, CDS, introns, and exons in gayal are comparable to those of the genomes used for homolog-based predictions (**Additional file 1: Figure S3**). In addition, we predicted non-coding RNA genes in the gayal genome. We used blast to search rRNA against Human rRNA database, and tRNAscan-SE (tRNAscan-SE , RRID:SCR_010835) [40] to search tRNA in the genome sequences. We also used blast to search miRNA and snRNA via Rfam database (Rfam , RRID:SCR_007891)(release 11.0) [41]. We reveal a total of 2,357 ribosomal RNA (rRNA), 29,821 transfer RNA (tRNA), 16,305 microRNAs (miRNA), and 1,380 snRNA genes in the gayal genome (**Additional file 1: Table S5**).

**Functional annotation of protein-coding genes**

Gene functional annotation referrers to searching functional motifs, domains, and possible biological process by aligning translated gene coding sequences to known

databases such as SwissProt and TrEMBL [42], NT database (from NCBI), Gene Ontology (GO)( GO , RRID:SCR_002811), and Kyoto Encyclopaedia of Genes and Genomes (KEGG , RRID:SCR_012773)(KEGG) [43]. We have annotated all the protein coding genes identified in this study to retrieve functional terms according to InterPro, KEGG, and GO terms. Overall, 81.74% (21,798), 54.56% (14,550), and 66.39% (17,704) genes show enrichment in InterPro, KEGG, and GO respectively. In total, 25,916 protein-coding genes (97.18%) were successfully annotated for conserved functional motifs and functional terms (**Additional file 1: Table S6**).

**Phylogenetic analysis and divergence time estimation**

To investigate the phylogenic position of gayal, we retrieved nucleotide and protein data for cattle (*Bos taurus*) [19], yak (*Bos grunniens*) [17], wisent (*Bison bonasus*) [20], bison (*Bison bison*)[21], zebu (*Bos indicus*) [22], and buffalo (*Bubalus bubalis*) [23] from the NCBI database. Gene ortholog relationships of gayal and other bovine species were identified by reciprocal blast searching with an e-value of 1e-7. Genes with alternative splicing variants are represented by the longest transcript. Multiple sequence alignment of the genes within one copy gene sets were performed using MUSCLE program (MUSCLE , RRID:SCR_011812) [44]. Aligned sequences were trimmed to remove potentially unreliably aligned regions and gaps using Gblocks [45]. Alignments with lengths shorter than 100bp were also discarded. Four-fold degenerate sites were extracted and concatenated into a supergene. Modeltest [46] was used to select the best substitution model. MrBayes (MrBayes ,

RRID:SCR_012067) [47] and RaxML (RAxML , RRID:SCR_006086) [48] software were used to reconstruct the evolutionary relationships between species, and MEGA5 [49] used to view the tree. From these analyses, gayal clusters with the common ancestor of cattle and zebu (**Figure 4**).

Additionally, we sequenced the complete mitochondrial DNA (mtDNA, the first complete mtDNA of the gayal submitted to GenBank: MF614103) using Sanger sequencing method, due to the fact that next generation sequencing methods have lower ability and accuracy in recovering repeat sequences [28, 50], particularly in regions with rich GC content like the D-loop. We then downloaded mtDNA sequences of gayal and other bovine species from GenBank for phylogenic analysis. As shown in **Figure 5** and **Figure S4**, the gayal we sequenced clusters with gaur (**Figure 5, Additional file 1: Figure S4**). Our results from both whole genome and mtDNA data differ from the conclusion made by Mei *et al.* who mapped gayal genome resequencing data to a bovine reference [5]. Furthermore, the MCMCTREE program, implemented using the PAML (PAML , RRID:SCR_014932)[51] package, was used to estimate divergence times. The JC69 model and correlated molecular clock rates (clock=3) were used in the calculation. Calibration time for the common ancestor of buffalo and cattle obtained from the TimeTree database (http://www.timetree.org/) was used to calibrate the divergence time. This analysis estimated the divergence time of gayal from cattle and zebu at approximately 5.1 million years ago (**Figure 6**).

In conclusion, we have constructed a *de novo* assembly of the gayal genome and

describe its genetic attributes. To our knowledge, this is the first *de novo* assembled genome for this species. We also demonstrate that together with the genomes of other bovine species, the new gayal genome supports investigations concerning the origin, evolutionary history, and local adaptation of gayal. This resource is also important for the future conservation of this endangered species. In addition, the *de novo* gayal genome adds to the list of available bovine genomes, and has advantages over resequenced genomes in allowing accurate whole genome alignment, retrieving constraint and/or rapidly evolved elements. It also strengthens the capacity to better assess introgression, incomplete lineage sorting (ILS), and structural variation (SV) among the bovine species, as well as inferring their effects on the species tree. The assembled genome could be used as a reference in population genomic studies [52] of the gayal. Furthermore, comprehensive comparative analyses of these genomes will improve understanding of the formation and speciation of bovine species.

## Availability of supporting data

The genome sequencing raw reads were deposited in the NCBI SRA database, project ID: PRJNA387130. The assembly and annotation of the gayal genome are available in the *GigaScience* GigaDB database[53]. The complete mtDNA for the gayal generated by Sanger sequencing is also available in GenBank under the ID: MF614103. All supplementary figures and tables are provided in Additional file 1.

## Competing interests

The authors declare that they have no competing interests.

## Authors' contributions

YPZ, DDW and MSW designed the study. WW and YD supervised the analyses. WHN, WTS and JHW cultivated the cells. YZ and XW performed genome assembly and annotation. MSW extracted genomic DNA and wrote manuscript with other author's input. MSW and SQY sequenced the gayal complete mitochondrial DNA and submitted to GenBank. SW, ZJX, KXQ, NOO, DY, DDW and YPZ revised the manuscript. All authors read and approved the final manuscript.

## Acknowledgements

This work was supported by the Strategic Priority Research Program of the Chinese A cademy of Sciences, Grant No. XDB13020600, and the Chinese 973 program (2013C B835200, 2013CB835204). DDW was supported by the Youth Innovation Promotion Association, Chinese Academy of Sciences. N.O.O. is thankful for the support of the CAS-TWAS President's Fellowship Program for Doctoral Candidates.

## References

1. Payne WJA, Hodges J: **Tropical cattle: origins, breeds and breeding policies.** 1997**:**Blackwell Science.
2. Uzzaman MR, Bhuiyan MS, Edea Z, Kim KS: **Semi-domesticated and Irreplaceable genetic resource gayal (*Bos frontalis*) needs effective genetic conservation in Bangladesh: a review.** *Asian-Australas J Anim Sci* 2014, **27:**1368-1372.
3. Miao YW, Ha F, Gao HS, Yuan F, Li DL, Yuan YY: **Polymorphisms of inhibin α gene**

**exon 1 in buffalo (*Bubalus bubalis*), gayal (*Bos frontalis*) and yak (*Bos grunniens*).** *Zool Res* 2012, **33:**402-408.

4. Payne WJA: **Cattle production in the tropics**. *Vol. 1. General introduction and breeds and breeding.* London: Longman Group Ltd.; 1970.

5. Mei C, Wang H, Zhu W, Wang H, Cheng G, Qu K, Guang X, Li A, Zhao C, Yang W, et al: **Whole-genome sequencing of the endangered bovine species gayal (*Bos frontalis*) provides new insights into its genetic features.** *Sci Rep* 2016, **6:**19787.

6. Winter H, Mayr B, Schleger W, Dworak E, Krutzler J, Burger B: **Karyotyping, red blood cell and haemoglobin typing of the mithun (*Bos frontalis*), its wild ancestor and its hybrids.** *Res Vet Sci* 1984, **36:**276-283.

7. Gallagher DS, Jr., Womack JE: **Chromosome conservation in the Bovidae.** *J Hered* 1992, **83:**287-298.

8. Shan XN, Chen YF, Luo LH, Cao XM, Song JZ, Zeng YZ: **Comparative studies on the chromosomes of five species of catties of the genus *Bos* in China.** *Zool Res* 1980, **1**:75-81.

9. Adbullah MH, Idris I, Hilmi M: **Karyotype of malayan gaur (*Bos gaurus hubbacki*), Sahiwal-Friesian cattle and gaur x cattle hybrid backcrosses.** *Pak J Biol Sci* 2009, **12:**896-901.

10. Qu KX, He ZX, Nie WH, Zhang JC, Jin XD, Yang GR, Yuan XP, Huang BZ, Zhang YP, Zan LS: **Karyotype analysis of mithun (*Bos frontalis*) and mithun bull x Brahman cow hybrids.** *Genet Mol Res* 2012, **11:**131-140.

11. Gou X, Wang Y, Yang S, Deng W, Mao H: **Genetic diversity and origin of gayal and cattle in Yunnan revealed by mtDNA control region and SRY gene sequence variation.** *J Anim Breed Genet* 2010, **127:**154-160.

12. Li SP, Chang H, Ma GL, Chen HY, Ji DJ, Geng RQ: **Molecular phylogeny of the gayal inferred from the analysis of cytochrome b gene entire sequences.** *Yi Chuan* 2008, **30:**65-70.

13. Nijman IJ, van Boxtel DCJ, van Cann LM, Marnoch Y, Cuppen E, Lenstra JA: **Phylogeny of Y chromosomes from bovine species**. Cladistics 2008, 24:723-726.

14. Dorji T, Mannen H, Namikawa T, Inamura T, Kawamoto Y: **Diversity and phylogeny of**

**mitochondrial DNA isolated from mithun *Bos frontalis* located in Bhutan.** *Anim Genet* 2010, **41:**554-556.

15. Tanaka K, Takizawa T, Murakoshi H, Dorji T, Nyunt MM, Maeda Y, Yamamoto Y, Namikawa T: **Molecular phylogeny and diversity of Myanmar and Bhutan mithun based on mtDNA sequences.** *Anim Sci J* 2011, **82:**52-56.

16. Baig M, Mitra B, Qu KX, Peng MS, Ahmed I, Miao YW, Zan LS, Zhang YP: **Mitochondrial DNA diversity and origin of *Bos frontalis*.** *Current Science* 2013, **104:**115-120.

17. Qiu Q, Zhang G, Ma T, Qian W, Wang J, Ye Z, Cao C, Hu Q, Kim J, Larkin DM, et al: **The yak genome and adaptation to life at high altitude.** *Nat Genet* 2012, **44:**946-949.

18. Porto-Neto LR, Sonstegard TS, Liu GE, Bickhart DM, Da Silva MV, Machado MA, Utsunomiya YT, Garcia JF, Gondro C, Van Tassell CP: **Genomic divergence of zebu and taurine cattle identified through high-density SNP genotyping.** *BMC Genomics* 2013, **14:**876.

19. Zimin AV, Delcher AL, Florea L, Kelley DR, Schatz MC, Puiu D, Hanrahan F, Pertea G, Van Tassell CP, Sonstegard TS, et al: **A whole-genome assembly of the domestic cow, *Bos taurus*.** *Genome Biol* 2009, **10:**R42.

20. Wang K, Wang L, Lenstra JA, Jian J, Yang Y, Hu Q, Lai D, Qiu Q, Ma T, Du Z, et al: **The genome sequence of the wisent (*Bison bonasus*).** *Gigascience*. 2017 Mar 10. doi: 10.1093/gigascience/gix016.

21. **American bison (*Bison bison bison*) genome assembly** https://www.ncbi.nlm.nih.gov/assembly/GCF_000754665.1/.

22. Canavez FC, Luche DD, Stothard P, Leite KR, Sousa-Canavez JM, Plastow G, Meidanis J, Souza MA, Feijao P, Moore SS, Camara-Lopes LH: **Genome sequence and assembly of *Bos indicus*.** *J Hered* 2012, **103:**342-348.

23. **Water buffalo (*Bubalus bubalis*) genome assembly** https://www.ncbi.nlm.nih.gov/assembly/GCA_000471725.1#/st.

24. Luo R, Liu B, Xie Y, Li Z, Huang W, Yuan J, He G, Chen Y, Pan Q, Liu Y, et al: **SOAPdenovo2: an empirically improved memory-efficient short-read de novo assembler.** *Gigascience* 2012, **1:**18.

25. Kajitani R, Toshimoto K, Noguchi H, Toyoda A, Ogura Y, Okuno M, Yabana M, Harada M, Nagayasu E, Maruyama H, et al: **Efficient de novo assembly of highly heterozygous genomes from whole-genome shotgun short reads.** *Genome Res* 2014, **24:**1384-1395.

26. Bovine Genome S, Analysis C, Elsik CG, Tellam RL, Worley KC, Gibbs RA, Muzny DM, Weinstock GM, Adelson DL, Eichler EE, et al: **The genome sequence of taurine cattle: a window to ruminant biology and evolution.** *Science* 2009, **324:**522-528.

27. Simao FA, Waterhouse RM, Ioannidis P, Kriventseva EV, Zdobnov EM: **BUSCO: assessing genome assembly and annotation completeness with single-copy orthologs.** *Bioinformatics* 2015, **31:**3210-3212.

28. Wang MS, Li Y, Peng MS, Zhong L, Wang ZJ, Li QY, Tu XL, Dong Y, Zhu CL, Wang L, et al: **Genomic analyses reveal potential independent adaptation to high altitude in Tibetan chickens.** *Mol Biol Evol* 2015, **32:**1880-1889.

29. Xiong Z, Li F, Li Q, Zhou L, Gamble T, Zheng J, Kui L, Li C, Li S, Yang H, Zhang G: **Draft genome of the leopard gecko, *Eublepharis macularius*.** *Gigascience* 2016, **5:**47.

30. Kapitonov VV, Jurka J: **A universal classification of eukaryotic transposable elements implemented in Repbase.** *Nat Rev Genet* 2008, **9:**411-412.

31. Edgar RC, Myers EW: **PILER: identification and classification of genomic repeats.** *Bioinformatics* 2005, **21 Suppl 1:**152-158.

32. Benson G: **Tandem repeats finder: a program to analyze DNA sequences.** *Nucleic Acids Res* 1999, **27:**573-580.

33. Lindblad-Toh K, Wade CM, Mikkelsen TS, Karlsson EK, Jaffe DB, Kamal M, Clamp M, Chang JL, Kulbokas EJ, 3rd, Zody MC, et al: **Genome sequence, comparative analysis and haplotype structure of the domestic dog.** *Nature* 2005, **438:**803-819.

34. Groenen MA, Archibald AL, Uenishi H, Tuggle CK, Takeuchi Y, Rothschild MF, Rogel-Gaillard C, Park C, Milan D, Megens HJ, et al: **Analyses of pig genomes provide insight into porcine demography and evolution.** *Nature* 2012, **491:**393-398.

35. Jiang Y, Xie M, Chen W, Talbot R, Maddox JF, Faraut T, Wu C, Muzny DM, Li Y, Zhang W, et al: **The sheep genome illuminates biology of the rumen and lipid metabolism.** *Science* 2014, **344:**1168-1173.

36. Stanke M, Keller O, Gunduz I, Hayes A, Waack S, Morgenstern B: **AUGUSTUS: ab**

**initio prediction of alternative transcripts.** *Nucleic Acids Res* 2006, **34:**W435-439.

37. Cai Y, Gonzalez JV, Liu Z, Huang T: **Computational systems biology methods in molecular biology, chemistry biology, molecular biomedicine, and biopharmacy.** *Biomed Res Int* 2014, **2014:**746814.

38. Majoros WH, Pertea M, Salzberg SL: **TigrScan and GlimmerHMM: two open source ab initio eukaryotic gene-finders.** *Bioinformatics* 2004, **20:**2878-2879.

39. Elsik CG, Mackey AJ, Reese JT, Milshina NV, Roos DS, Weinstock GM: **Creating a honey bee consensus gene set.** *Genome Biol* 2007, **8:**R13.

40. Lowe TM, Eddy SR: **tRNAscan-SE: a program for improved detection of transfer RNA genes in genomic sequence.** *Nucleic Acids Res* 1997, **25:**955-964.

41. Gardner PP, Daub J, Tate J, Moore BL, Osuch IH, Griffiths-Jones S, Finn RD, Nawrocki EP, Kolbe DL, Eddy SR, Bateman A: **Rfam: Wikipedia, clans and the "decimal" release.** *Nucleic Acids Res* 2011, **39:**D141-145.

42. UniProt C: **UniProt: a hub for protein information.** *Nucleic Acids Res* 2015, **43:**D204-212.

43. Kanehisa M, Goto S, Sato Y, Kawashima M, Furumichi M, Tanabe M: **Data, information, knowledge and principle: back to metabolism in KEGG.** *Nucleic Acids Res* 2014, **42:**D199-205.

44. Edgar RC: **MUSCLE: a multiple sequence alignment method with reduced time and space complexity.** *BMC Bioinformatics* 2004, **5:**113.

45. Talavera G, Castresana J: **Improvement of phylogenies after removing divergent and ambiguously aligned blocks from protein sequence alignments.** *Syst Biol* 2007, **56:**564-577.

46. Posada D: **Using MODELTEST and PAUP* to select a model of nucleotide substitution.** *Curr Protoc Bioinformatics* 2003, **Chapter 6:**Unit 6 5.

47. Huelsenbeck JP, Ronquist F: **MRBAYES: Bayesian inference of phylogenetic trees.** *Bioinformatics* 2001, **17:**754-755.

48. Stamatakis A: **RAxML-VI-HPC: maximum likelihood-based phylogenetic analyses with thousands of taxa and mixed models.** *Bioinformatics* 2006, **22:**2688-2690.

49. Tamura K, Peterson D, Peterson N, Stecher G, Nei M, Kumar S: **MEGA5: molecular**

**evolutionary genetics analysis using maximum likelihood, evolutionary distance, and maximum parsimony methods.** *Mol Biol Evol* 2011, **28:**2731-2739.

50. Wang MS, Yang HC, Otecko NO, Wu DD, Zhang YP: **Olfactory genes in Tibetan wild boar.** *Nat Genet* 2016, **48:**972-973.

51. Yang Z: **PAML 4: phylogenetic analysis by maximum likelihood.** *Mol Biol Evol* 2007, **24:**1586-1591.

52. Chen H: **Population genetic studies in the genomic sequencing era**. *Zool Res* 2015, **36:** 223-232.

53. Wu, D, D; Wang, M, S; Zeng, Y; Wang, X; Nie, W, H; Wang, J, H; Su, W, T; Otecko, N, O; Xiong, Z, J; Wang, S; Qu, K, X; Wang, W; Dong, Y; Zhang, Y, P (2017): Supporting data for "Draft genome of the Gayal, Bos frontalis" GigaScience Database. http://dx.doi.org/10.5524/100354

## Figure Legends:

Figure 1. A picture showing a female gayal (*Bos frontalis, provided by Kai-Xing Qu*).

Figure 2. Karyotype of the gayal used for genome sequencing (*provided by Wen-Hui Nie*).

Figure 3. 17-mer frequency distribution of sequencing reads.

Figure 4. Phylogenetic trees of gayal and other bovine species. (A) Tree constructed based on maximum likelihood method, (B) Tree constructed using Bayesian inference.

Figure 5. Maximum likelihood trees of gayal and other bovine species using whole complete mtDNA. IDs in parentheses are GenBank accession number.

Figure 6. Divergence time estimated between gayal and other bovine species.

## Tables:

Table 1. Statistics of the completeness of the hybrid *de novo* assembly of *Bos frontalis* genome

| Terms | Contig | | Scaffold | |
|---|---|---|---|---|
| | Size | number | Size | number |
| **N90** | 2,461 | 211577 | 158,610 | 1357 |
| **N80** | 5,335 | 140237 | 1,060,177 | 800 |
| **N70** | 8,109 | 99930 | 1,668,147 | 587 |
| **N60** | 11,044 | 71764 | 2,170,469 | 437 |
| **N50** | 14,405 | 50585 | 2,737,757 | 320 |
| **Max length** | 208,099 | | 13,764,521 | |
| **Total length** | 2,669,378,334 | | 2,848,570,279 | |
| **Total number** | | 583373 | | 460,059 |
| **Average length** | 4575 | | 6,191 | |
| **Number>=500bp** | | 394757 | | 116481 |
| **Number>=1000bp** | | 300178 | | 53989 |
| **Number>=2000bp** | | 229796 | | 19915 |
| **Number>=5000bp** | | 146493 | | 5387 |

Table 2. Statistics of the completeness of the assembled genomes for *Bos frontalis* and close related species by BUSCO (version 2)

| Species | Terms | Complete(C) | Complete and single-copy (S) | Complete and duplicated (D) | Fragmented (F) | Missing (M) |
|---|---|---|---|---|---|---|
| **gayal** | **Number** | 3494 | 3434 | 60 | 319 | 291 |
| | **Proportion** | 85.14% | 83.67% | 1.46% | 7.77% | 7.09% |
| **zebu** | **Number** | 3698 | 3644 | 54 | 158 | 248 |
| | **Proportion** | 90.11% | 88.79% | 1.32% | 3.85% | 6.04% |
| **wisent** | **Number** | 3794 | 3763 | 31 | 180 | 130 |
| | **Proportion** | 92.45% | 91.69% | 0.76% | 4.39% | 3.17% |
| **yak** | **Number** | 3841 | 3809 | 32 | 138 | 125 |
| | **Proportion** | 93.59% | 92.81% | 0.78% | 3.36% | 3.05% |
| **buffalo** | **Number** | 3817 | 3780 | 37 | 142 | 145 |
| | **Proportion** | 93.01% | 92.11% | 0.90% | 3.46% | 3.53% |
| **bison** | **Number** | 3779 | 3735 | 44 | 165 | 160 |
| | **Proportion** | 92.08% | 91.01% | 1.07% | 4.02% | 3.90% |

Table 3. Statistics of repeats in *Bos frontalis* genome.

| **Type** | **Repeat Size （bp）** | **% of genome** |
|---|---|---|
| **Trf** | 17,696,175 | 0.62 |
| **Repeatmasker** | 868,885,926 | 30.50 |
| **Proteinmask** | 265,003,148 | 9.30 |
| ***De novo*** | 917,371,710 | 32.20 |
| **Total** | 1,371,023,312 | 48.13 |

Table 4. General statistics of predicted protein-coding genes.

| **Gene set** | | **Total** | **Exon number** | **CDS length (bp)** | **mRNA length (bp)** | **Exons per gene** | **Exon length (bp)** | **Intron length (bp)** |
|---|---|---|---|---|---|---|---|---|
| **Homolog** | ***Bos taurus*** | 19,666 | 141,323 | 1,325 | 20,618 | 7.19 | 184 | 3,118 |
| | ***Canis familiaris*** | 17,627 | 121,986 | 1,323 | 20,802 | 6.92 | 191 | 3,290 |
| | ***Homo sapiens*** | 24,783 | 146,172 | 1,108 | 17,567 | 5.89 | 187 | 3,360 |
| | ***Sus scrofa*** | 20,283 | 121,282 | 1,142 | 16,288 | 5.97 | 191 | 3,041 |
| | ***Rattus norvegicus*** | 17,988 | 117,965 | 1,277 | 19,469 | 6.55 | 194 | 3,273 |
| | ***Ovis aries*** | 20,947 | 147,367 | 1,287 | 20,973 | 7.03 | 183 | 3,261 |
| ***De novo*** | **AUGUSTUS** | 41,227 | 180,664 | 1,127 | 22,786 | 4.38 | 257 | 6,403 |
| | **GlimmerHMM** | 27,067 | 104,294 | 874 | 5,433 | 3.85 | 226 | 1,597 |
| | **Genescan** | 46,598 | 297,828 | 1,321 | 36,828 | 6.39 | 206 | 6,585 |
| **Glean (final)** | | 26,667 | 87,392 | 1,156 | 4,996 | 3.27 | 352 | 1,686 |

Figure1

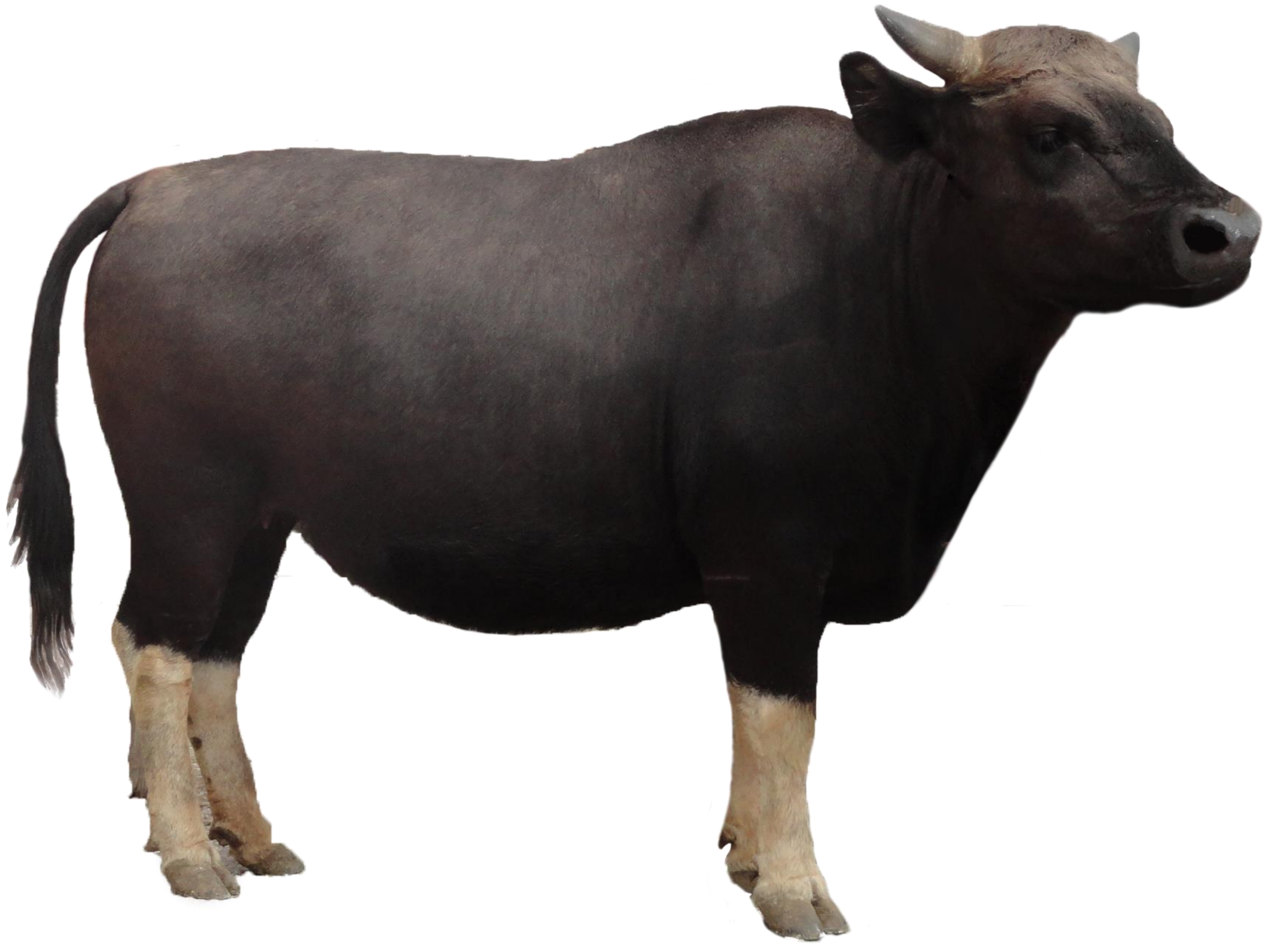

Click here to download Figure Figure1.pdf

Click here to download Figure Figure2.jpg

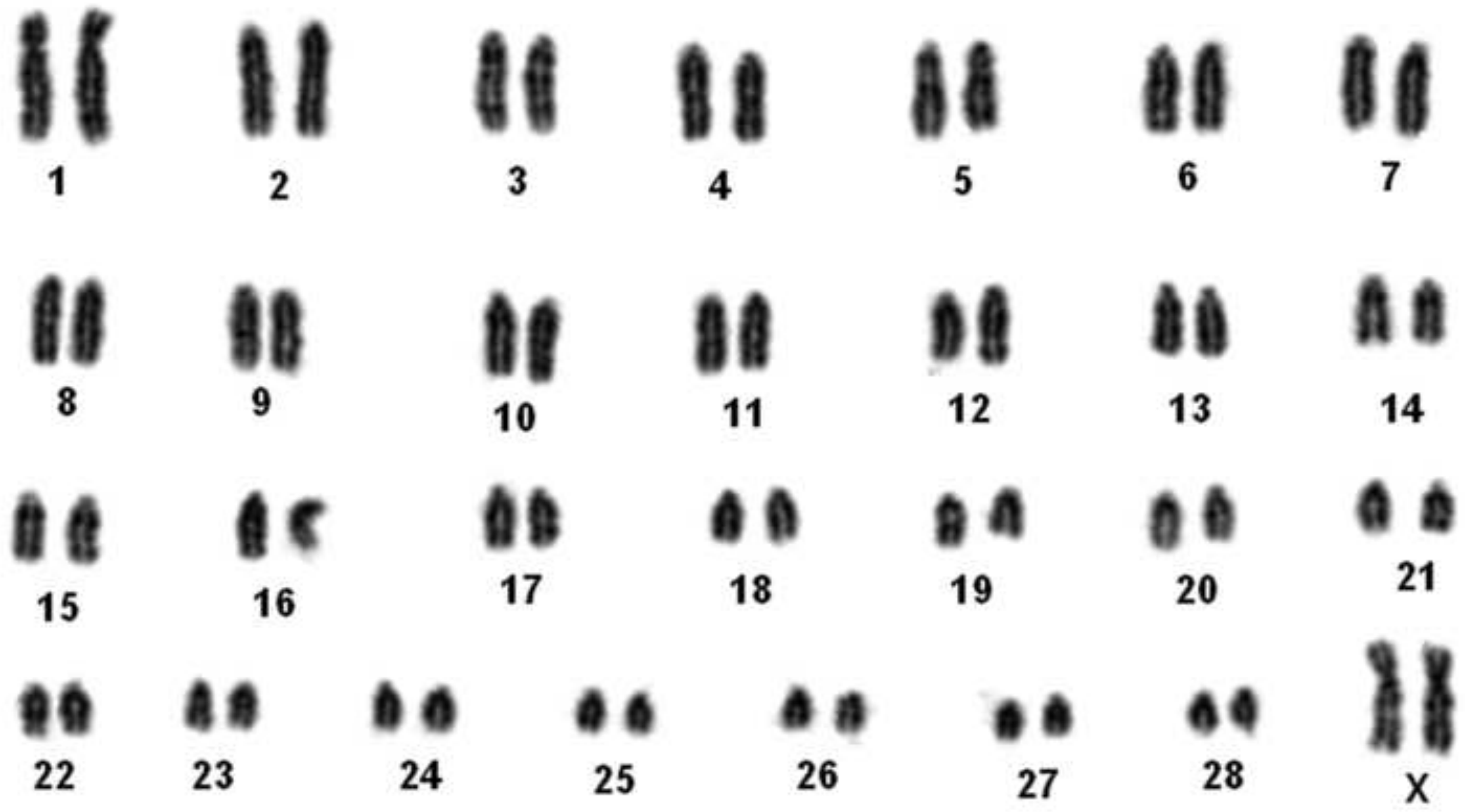

Figure3

Click here to download Figure Figure3-K.pdf

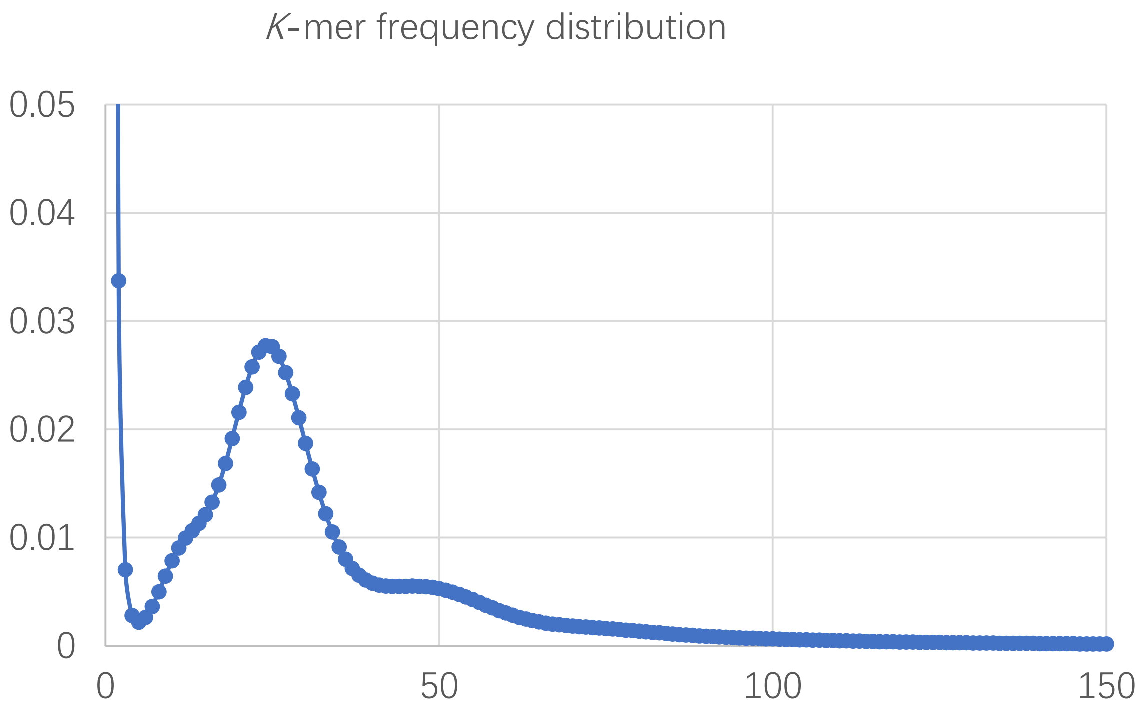

Figure4

Click here to download Figure Figure4.pdf

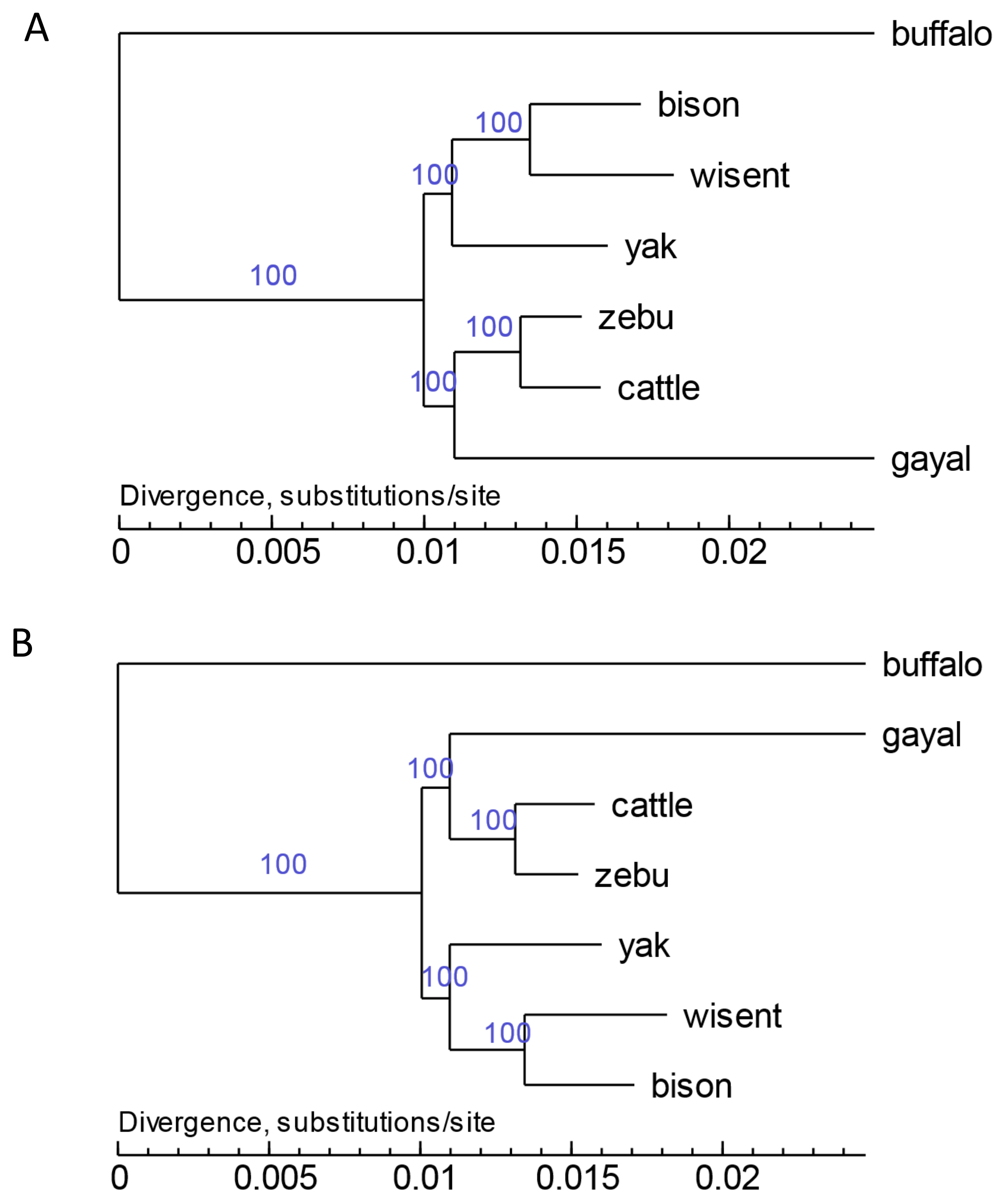

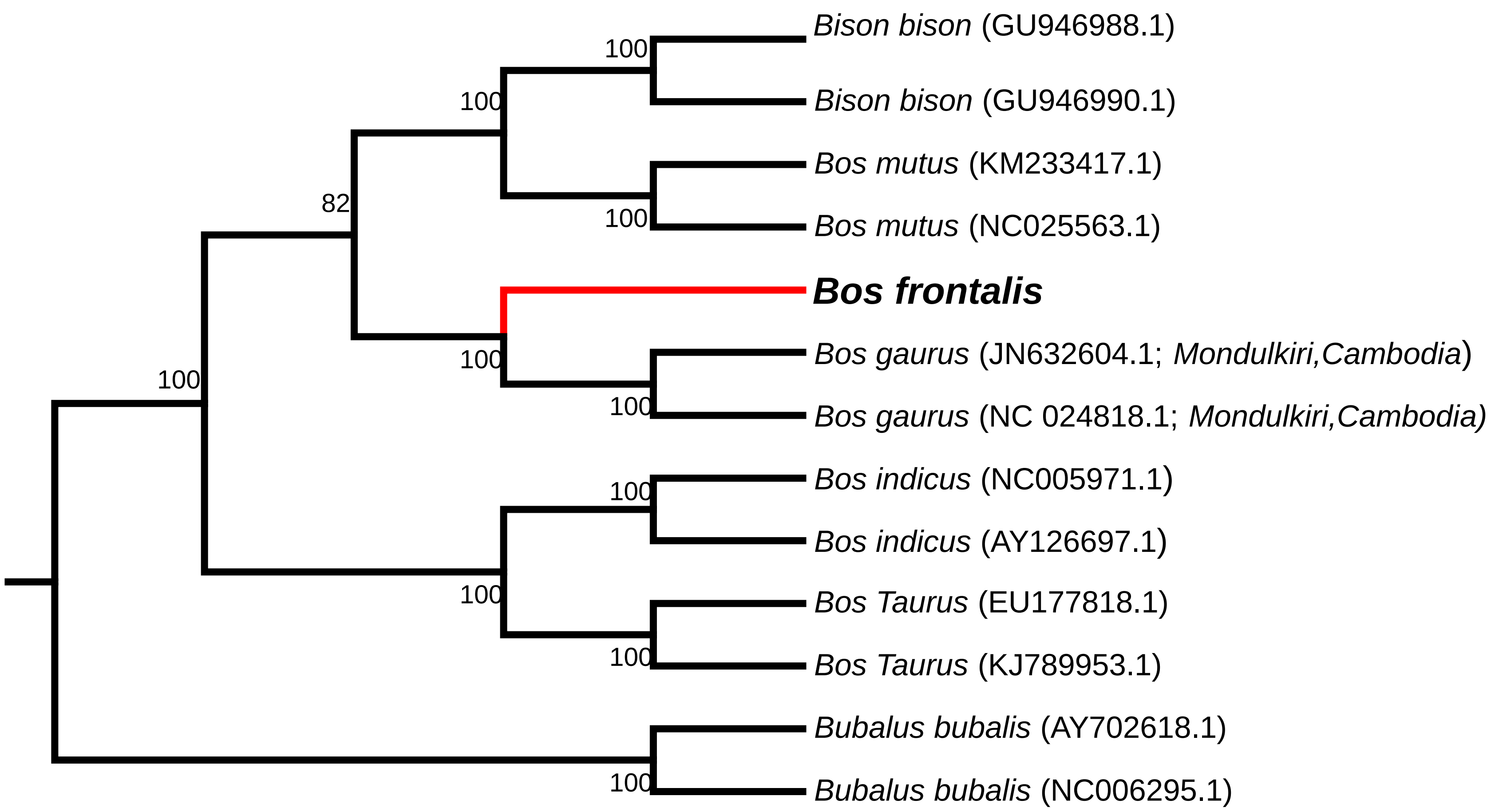
Bison bison (GU946988.1)
Bison bison (GU946990.1)
Bos mutus (KM233417.1)
Bos mutus (NC025563.1)
Bos frontalis
Bos gaurus (JN632604.1; Mondulkiri,Cambodia)
Bos gaurus (NC 024818.1; Mondulkiri,Cambodia)
Bos indicus (NC005971.1)
Bos indicus (AY126697.1)
Bos Taurus (EU177818.1)
Bos Taurus (KJ789953.1)
Bubalus bubalis (AY702618.1)
Bubalus bubalis (NC006295.1)
100
100
100
82
100
100
100
100
100
100
100
100

 Click here to download Figure Figure6.pdf

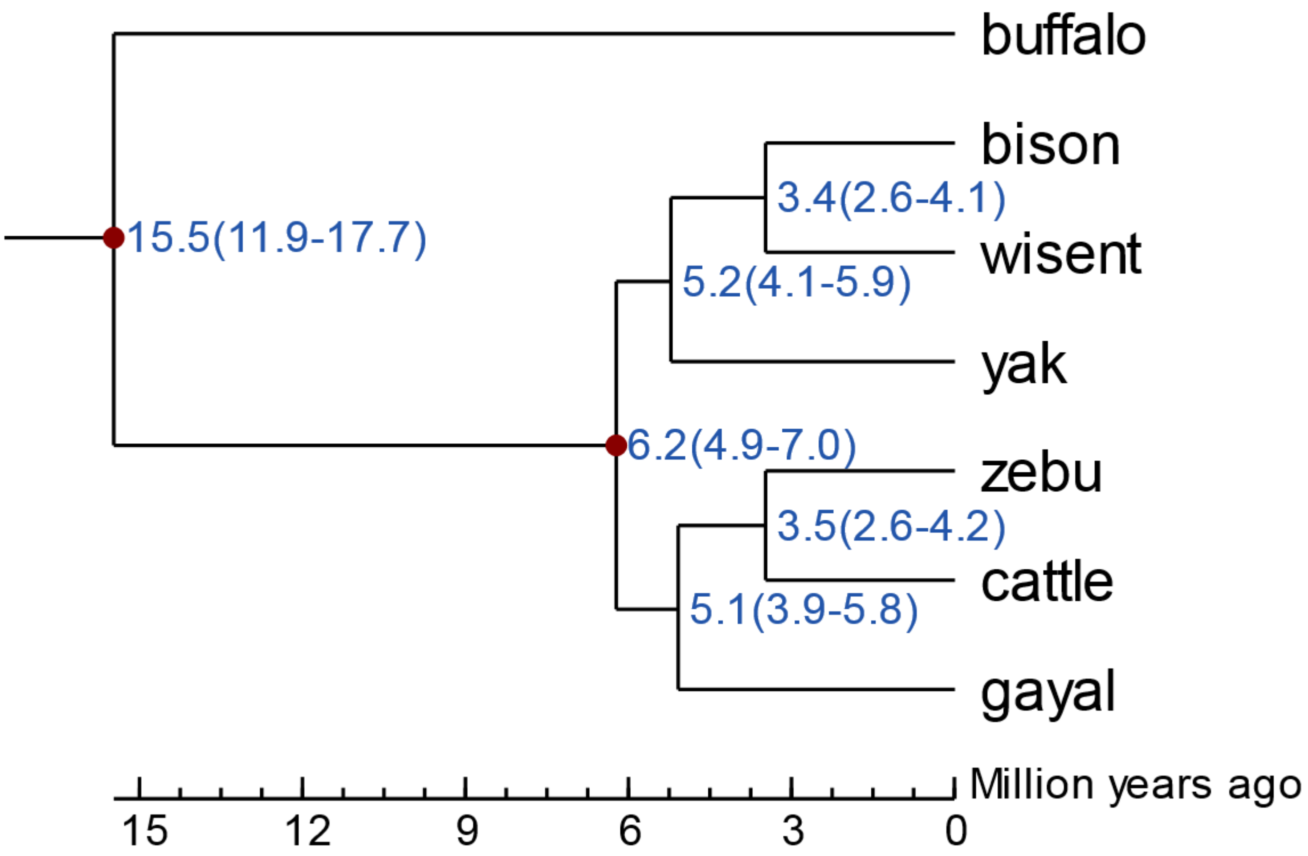

Click here to access/download
**Supplementary Material**
SupplementaryInfor.doc

23 August 2017

To
The Editorial Office,
GigaScience.

Dear Editor,

**RE: Draft genome of the gayal, *Bos frontalis.***

We are very grateful for the constructive review and the valuable suggestions received on our paper titled above (GIGA-D-17-00116). We have carefully considered all the comments and suggestions. It is with much pleasure that we re-submit the revised manuscript for your consideration for publication in your highly reputable journal.

We have carefully revised our citations and references, sample origin, and the novelty of our research. We have also re-estimated the genome size using K-mer ratio using only the reads that have passed quality filtering. For a detailed description of all the changes in the manuscript, please find a separate point-by-point response to each of the comments raised by the editor and the reviewers.

We believe the review has greatly improved our manuscript and that our revisions sufficiently address all the review comments. Thank you in advance for considering this manuscript for publication.

Yours sincerely,
Dong-Dong Wu and Co-authors
