# Supplementary material for: Draft genome of the gayal, Bos frontalis
Source: Gigascience. 2017 Oct 5;6(11):1–7. doi: 10.1093/gigascience/gix094 (PMC5710521; doi:10.1093/gigascience/gix094)

**Table S1 Statistics of raw data**

| **Insert Size (bp)** | **Library ID** | **Raw Reads(M)** | **Reads Length (bp)** | **Total Data(Gb)** |
| --- | --- | --- | --- | --- |
| 180 | D-2-180-3-350 | 137.67 | 100 | 27.53 |
| 180 | NiuD-2-180-1 | 229.19 | 100 | 45.83 |
| 250 | niu-D-250-370bp | 108.89 | 100 | 21.77 |
| 450 | D-1-450 | 215.18 | 100 | 44.32 |
| 450 | niu-450-570bp | 72.73 | 100 | 14.54 |
| 600 | D-1-600 | 211.06 | 100 | 43.47 |
| 600 | niu-600-720bp | 179.63 | 100 | 35.92 |
| 800 | SZAMPI029634-84 | 124.93 | 85 | 21.23 |
| 800 | SZAMPI029635-84 | 127.25 | 85 | 21.63 |
| 2000 | BOSdakDAMDWAAPEI-57 | 125.39 | 49 | 12.28 |
| 2000 | BOSdakDAMDWBAPEI-62 | 120.06 | 49 | 11.76 |
| 5000 | BOSdakDAPDLAAPEI-74 | 119.48 | 49 | 11.70 |
| 5000 | BOSdakDAPDLBAPEI-75 | 116.16 | 49 | 11.38 |
| 10000 | BOSdakDAZDTAAPEI-87 | 61.52 | 49 | 6.02 |
| 10000 | BOSdakDAZDTBAPEI-88 | 97.72 | 49 | 9.57 |
| 20000 | BOSdakDAZDUAAPEI-18 | 68.24 | 49 | 6.68 |
| 20000 | BOSdakDAZDUBAPEI-19 | 48.53 | 49 | 4.75 |
| Total | - | - | - | 350.38 |

**Table S2 Statistics of clean data after filtering**

| **Insert Size (bp)** | **Library ID** | **Clean Reads（M）** | **Reads Length (bp)** | **Total Data(Gb)** |
| --- | --- | --- | --- | --- |
| 180 | D-2-180-3-350 | 129.51 | 96 | 24.865 |
| 180 | NiuD-2-180-1 | 202.37 | 96 | 38.855 |
| 250 | niu-D-250-370bp | 98.62 | 96 | 18.936 |
| 450 | D-1-450 | 98.04 | 96 | 18.824 |
| 450 | niu-450-570bp | 64.14 | 96 | 12.316 |
| 600 | D-1-600 | 93.29 | 96 | 17.913 |
| 600 | niu-600-720bp | 146.62 | 96 | 28.152 |
| 800 | SZAMPI029634-84 | 124.93 | 85 | 21.23 |
| 800 | SZAMPI029635-84 | 127.25 | 85 | 21.63 |
| 2000 | BOSdakDAMDWAAPEI-57 | 125.39 | 49 | 12.28 |
| 2000 | BOSdakDAMDWBAPEI-62 | 120.06 | 49 | 11.76 |
| 5000 | BOSdakDAPDLAAPEI-74 | 119.48 | 49 | 11.70 |
| 5000 | BOSdakDAPDLBAPEI-75 | 116.16 | 49 | 11.38 |
| 10000 | BOSdakDAZDTAAPEI-87 | 61.52 | 49 | 6.02 |
| 10000 | BOSdakDAZDTBAPEI-88 | 97.72 | 49 | 9.57 |
| 20000 | BOSdakDAZDUAAPEI-18 | 68.24 | 49 | 6.68 |
| 20000 | BOSdakDAZDUBAPEI-19 | 48.53 | 49 | 4.75 |
| Total | - | - | - | 276.861 |

**Table S3 Statistics of 17-mer analysis**

| **Kmer** | **Kmer_num** | **Peak depth** | **genome_size** | **used_base** | **used_read** | **X** |
| --- | --- | --- | --- | --- | --- | --- |
| 17 | 79702571328 | 25.0399 | 3155773450 | 203,652,243,867 | 2,167,240,756 | 27.7199 |

All 17-mer sequences were extracted from paired-end clean reads that passed quality control (QC) from short insert size libraries (180bp and 450bp), and the frequency of each 17-mer was calculated and plotted. Using the formula the genome size G=K_num/Peak_depth , the genome size was estimated to be 3.15Gb (haploid).

|  | **Repbase TEs** | | **TE protiens** | | **De novo** | | **Combined TEs** | |
| --- | --- | --- | --- | --- | --- | --- | --- | --- |
| **Type** | Length (Bp) | % in genome | Length (Bp) | % in genome | Length (Bp) | % in genome | Length (Bp) | % in genome |
| **DNA** | 54,940,002 | 1.93 | 5,567,809 | 0.19 | 7,063,594 | 0.25 | 63,340,997 | 2.22 |
| **LINE** | 496,518,580 | 17.43 | 251,201,514 | 8.82 | 767,121,061 | 26.93 | 1,151,551,996 | 40.43 |
| **SINE** | 217,569,344 | 7.64 | - | - | 39,543,108 | 1.39 | 230,711,022 | 8.1 |
| **LTR** | 118,337,474 | 4.15 | 8,264,965 | 0.29 | 304,652,815 | 10.69 | 397,862,466 | 13.97 |
| **Other** | 824 | 0.000029 | - | - | - | - | 824 | 0.000029 |
| **Satellite** | - | - | - | - | 7,951,338 | 0.28 | 7,951,338 | 0.28 |
| **Simple_repeat** | - | - | - | - | 264,689 | 0.0093 | 264,689 | 0.0092 |
| **Low_complexity** | - | - | - | - | - | - | - | - |
| **Tandem_Repeat** | - | - | - | - | - | - | - | - |
| **Unknown** | - | - | - | - | 632,762 | 0.022 | 632,762 | 0.022 |
| **Total** | 868,885,926 | 30.5 | 265,003,148 | 9.3 | 917,371,710 | 32.2 | 1,363,583,087 | 47.87 |

**Table S4 TEs Content in the Assembled Bos frontalis Genome**

Note: Repbase TEs: the result of RepeatMasker based on Repbase; TE proteins: the result of RepeatProteinMask based on Repbase; RepeatMasker: de novo finding repeats (Reaptmodeler); Combined: combine the results of Repbase TEs and TE proteins.

**Table S5 TEs Content (classified) in the Assembled *Bos frontalis* Genome.**

| **#Type** | **All**  **(Number)** | **All**  **(Ratio)** | **De novo (Number)** | **De novo**  **(Ratio)** | **Known**  **(Number)** | **Known**  **(Ratio)** |
| --- | --- | --- | --- | --- | --- | --- |
| DNA_transposons | 128958 | 11.401 | 79461 | 10.329 | 94086 | 11.42 |
| DNA/CMC-EnSpm | 64285 | 5.683 | 53257 | 6.923 | 31662 | 3.843 |
| DNA/MULE-MuDR | 8869 | 0.784 | 5497 | 0.715 | 6631 | 0.805 |
| DNA/DNA | 4660 | 0.412 | 0 | 0 | 7178 | 0.871 |
| DNA/PIF-Harbinger | 4995 | 0.442 | 3743 | 0.487 | 1956 | 0.237 |
| DNA/hAT-Tip100 | 4146 | 0.367 | 1605 | 0.209 | 4166 | 0.506 |
| DNA/hAT-Tag1 | 4380 | 0.387 | 3819 | 0.496 | 1024 | 0.124 |
| DNA/P | 3402 | 0.301 | 208 | 0.027 | 5169 | 0.627 |
| DNA/hAT-Charlie | 3716 | 0.329 | 2411 | 0.313 | 2553 | 0.31 |
| DNA/others | 3710 | 0.328 | 3903 | 0.507 | 0 | 0 |
| DNA/TcMar-Fot1 | 2966 | 0.262 | 7 | 0.001 | 4624 | 0.561 |
| DNA/CMC-Chapaev | 2725 | 0.241 | 232 | 0.03 | 3944 | 0.479 |
| DNA/MuDR | 3030 | 0.268 | 2403 | 0.312 | 1237 | 0.15 |
| DNA/CMC-Transib | 2229 | 0.197 | 320 | 0.042 | 2855 | 0.347 |
| DNA/hAT-Ac | 2271 | 0.201 | 380 | 0.049 | 2603 | 0.316 |
| DNA/Ginger | 2028 | 0.179 | 0 | 0 | 3068 | 0.372 |
| DNA/Maverick | 2080 | 0.184 | 64 | 0.008 | 2673 | 0.324 |
| DNA/Sola | 1380 | 0.122 | 59 | 0.008 | 2361 | 0.287 |
| DNA/hAT-hATm | 1325 | 0.117 | 161 | 0.021 | 1817 | 0.221 |
| DNA/hAT | 1227 | 0.108 | 43 | 0.006 | 1979 | 0.24 |
| DNA/TcMar-Pogo | 1305 | 0.115 | 787 | 0.102 | 790 | 0.096 |
| DNA/TcMar | 501 | 0.044 | 42 | 0.005 | 685 | 0.083 |
| DNA/Kolobok-T2 | 492 | 0.043 | 116 | 0.015 | 608 | 0.074 |
| DNA/Novosib | 394 | 0.035 | 3 | 0 | 814 | 0.099 |
| DNA/TcMar-Stowaway | 415 | 0.037 | 0 | 0 | 652 | 0.079 |
| DNA/hAT-hATw | 380 | 0.034 | 156 | 0.02 | 316 | 0.038 |
| DNA/Academ | 306 | 0.027 | 0 | 0 | 486 | 0.059 |
| DNA/TcMar-ISRm11 | 294 | 0.026 | 0 | 0 | 498 | 0.06 |
| DNA/CMC-Chapaev-3 | 290 | 0.026 | 0 | 0 | 438 | 0.053 |
| DNA/PiggyBac | 196 | 0.017 | 0 | 0 | 299 | 0.036 |
| DNA/hAT-hAT5 | 145 | 0.013 | 18 | 0.002 | 197 | 0.024 |
| DNA/Kolobok-Hydra | 148 | 0.013 | 44 | 0.006 | 140 | 0.017 |
| DNA/TcMar-Tc1 | 112 | 0.01 | 39 | 0.005 | 99 | 0.012 |
| DNA/Harbinger | 77 | 0.007 | 0 | 0 | 151 | 0.018 |
| DNA/hAT-hobo | 94 | 0.008 | 93 | 0.012 | 12 | 0.001 |
| DNA/IS | 65 | 0.006 | 0 | 0 | 65 | 0.008 |
| DNA/MULE-NOF | 56 | 0.005 | 18 | 0.002 | 55 | 0.007 |
| DNA/TcMar-Tigge | 41 | 0.004 | 0 | 0 | 50 | 0.006 |
| DNA/hAT-Pegasus | 36 | 0.003 | 0 | 0 | 51 | 0.006 |
| DNA/hAT-Blackjack | 30 | 0.003 | 0 | 0 | 33 | 0.004 |
| DNA/TcMar-Tigger | 23 | 0.002 | 0 | 0 | 29 | 0.004 |
| DNA/En-Spm | 20 | 0.002 | 0 | 0 | 20 | 0.002 |
| DNA/IS4EU | 18 | 0.002 | 15 | 0.002 | 3 | 0 |
| DNA/Merlin | 16 | 0.001 | 0 | 0 | 18 | 0.002 |
| DNA/Kolobok | 16 | 0.001 | 15 | 0.002 | 2 | 0 |
| DNA/TcMar-IS630 | 16 | 0.001 | 0 | 0 | 16 | 0.002 |
| DNA/TcMar-Tc2 | 8 | 0.001 | 2 | 0 | 11 | 0.001 |
| DNA/PIF-ISL2EU | 7 | 0.001 | 0 | 0 | 9 | 0.001 |
| DNA/TcMar-ISRm1 | 8 | 0.001 | 0 | 0 | 8 | 0.001 |
| DNA/Zator | 7 | 0.001 | 1 | 0 | 6 | 0.001 |
| DNA/hAT-hATx | 4 | 0 | 0 | 0 | 4 | 0 |
| DNA/TcMar-Mariner | 3 | 0 | 0 | 0 | 3 | 0 |
| DNA/MULE-F | 2 | 0 | 0 | 0 | 3 | 0 |
| DNA/TcMar-Sagan | 2 | 0 | 0 | 0 | 3 | 0 |
| DNA/TcMar-Tc4 | 2 | 0 | 0 | 0 | 3 | 0 |
| DNA/TcMar-Ant1 | 1 | 0 | 0 | 0 | 3 | 0 |
| DNA/P-Fungi | 1 | 0 | 0 | 0 | 2 | 0 |
| DNA/CMC-Mirage | 1 | 0 | 0 | 0 | 1 | 0 |
| DNA/TcMar-m44 | 1 | 0 | 0 | 0 | 1 | 0 |
| DNA/hAT-Restless | 1 | 0 | 0 | 0 | 1 | 0 |
| DNA/hAT-Gulliver | 0 | 0 | 0 | 0 | 1 | 0 |
| Retrotransposons | 720926 | 63.735 | 672236 | 87.382 | 257452 | 31.249 |
| LINE/L2 | 24653 | 2.18 | 1098 | 0.143 | 47071 | 5.713 |
| LINE/RTE-BovB | 30131 | 2.664 | 22760 | 2.959 | 18748 | 2.276 |
| LINE/L1 | 25285 | 2.235 | 17352 | 2.256 | 16533 | 2.007 |
| LINE/R2 | 1510 | 0.133 | 54 | 0.007 | 1822 | 0.221 |
| LINE/Penelope | 1150 | 0.102 | 116 | 0.015 | 1704 | 0.207 |
| LINE/Jockey | 723 | 0.064 | 124 | 0.016 | 631 | 0.077 |
| LINE/R1 | 607 | 0.054 | 0 | 0 | 707 | 0.086 |
| LINE/CR1 | 216 | 0.019 | 64 | 0.008 | 227 | 0.028 |
| LINE/L1-Tx1 | 124 | 0.011 | 14 | 0.002 | 170 | 0.021 |
| LINE/I | 107 | 0.009 | 66 | 0.009 | 74 | 0.009 |
| LINE/DRE | 65 | 0.006 | 0 | 0 | 84 | 0.01 |
| LINE/Proto1 | 62 | 0.005 | 0 | 0 | 80 | 0.01 |
| LINE/Rex-Babar | 51 | 0.005 | 0 | 0 | 77 | 0.009 |
| LINE/Dong-R4 | 31 | 0.003 | 0 | 0 | 41 | 0.005 |
| LINE/RTE-X | 24 | 0.002 | 0 | 0 | 31 | 0.004 |
| LINE/CRE | 21 | 0.002 | 12 | 0.002 | 10 | 0.001 |
| LINE/Ambal | 10 | 0.001 | 0 | 0 | 18 | 0.002 |
| LINE/LOA | 9 | 0.001 | 0 | 0 | 14 | 0.002 |
| LINE/Tad1 | 9 | 0.001 | 0 | 0 | 10 | 0.001 |
| LINE/LINE | 7 | 0.001 | 0 | 0 | 9 | 0.001 |
| LINE/Zorro | 4 | 0 | 0 | 0 | 6 | 0.001 |
| LINE/CR1-Zenon | 4 | 0 | 0 | 0 | 4 | 0 |
| LINE/RTE-RTE | 3 | 0 | 0 | 0 | 5 | 0.001 |
| LINE/RTE | 2 | 0 | 0 | 0 | 3 | 0 |
| LINE/CR1-L2 | 2 | 0 | 0 | 0 | 2 | 0 |
| LINE/Proto2 | 1 | 0 | 0 | 0 | 2 | 0 |
| LINE/I-Nimb | 1 | 0 | 0 | 0 | 1 | 0 |
| LINE/Odin | 1 | 0 | 0 | 0 | 1 | 0 |
| LTR/Gypsy | 423133 | 37.408 | 432358 | 56.201 | 109190 | 13.253 |
| LTR/others | 110825 | 9.798 | 116694 | 15.169 | 0 | 0 |
| LTR/Copia | 91295 | 8.071 | 77703 | 10.1 | 40909 | 4.966 |
| LTR/LTR | 4070 | 0.36 | 0 | 0 | 14633 | 1.776 |
| LTR/ERV1 | 1304 | 0.115 | 80 | 0.01 | 2024 | 0.246 |
| LTR/Ngaro | 1517 | 0.134 | 1263 | 0.164 | 417 | 0.051 |
| LTR/Pao | 1078 | 0.095 | 747 | 0.097 | 448 | 0.054 |
| LTR/Caulimovirus | 1013 | 0.09 | 921 | 0.12 | 128 | 0.016 |
| LTR/ERVK | 476 | 0.042 | 156 | 0.02 | 484 | 0.059 |
| LTR/Caulimoviru | 180 | 0.016 | 0 | 0 | 362 | 0.044 |
| LTR/DIRS | 155 | 0.014 | 125 | 0.016 | 46 | 0.006 |
| LTR/ERVL | 69 | 0.006 | 0 | 0 | 114 | 0.014 |
| LTR/Gypsy-Cigr | 22 | 0.002 | 0 | 0 | 58 | 0.007 |
| LTR/Gypsy-Gmr1 | 33 | 0.003 | 29 | 0.004 | 11 | 0.001 |
| LTR/Foamy | 8 | 0.001 | 0 | 0 | 18 | 0.002 |
| LTR/Lenti | 6 | 0.001 | 0 | 0 | 7 | 0.001 |
| LTR/ERVL-MaLR | 4 | 0 | 0 | 0 | 5 | 0.001 |
| LTR/Viper | 3 | 0 | 0 | 0 | 5 | 0.001 |
| LTR/ERV-Foamy | 1 | 0 | 0 | 0 | 3 | 0 |
| LTR/Gypsy-Troyka | 1 | 0 | 0 | 0 | 3 | 0 |
| LTR/Gypsy-Troyk | 0 | 0 | 0 | 0 | 2 | 0 |
| LTR/Tate | 1 | 0 | 0 | 0 | 1 | 0 |
| LTR/Delta | 0 | 0 | 0 | 0 | 1 | 0 |
| SINE/ID | 655 | 0.058 | 500 | 0.065 | 190 | 0.023 |
| SINE/B4 | 120 | 0.011 | 0 | 0 | 131 | 0.016 |
| SINE/SINE | 17 | 0.002 | 0 | 0 | 40 | 0.005 |
| SINE/tRNA-Lys | 24 | 0.002 | 0 | 0 | 25 | 0.003 |
| SINE/tRNA-RTE | 20 | 0.002 | 0 | 0 | 20 | 0.002 |
| SINE/Salmon | 17 | 0.002 | 0 | 0 | 17 | 0.002 |
| SINE/MIR | 11 | 0.001 | 0 | 0 | 11 | 0.001 |
| SINE/C | 9 | 0.001 | 0 | 0 | 10 | 0.001 |
| SINE/tRNA-Glu | 9 | 0.001 | 0 | 0 | 9 | 0.001 |
| SINE/7SL | 7 | 0.001 | 0 | 0 | 8 | 0.001 |
| SINE/BovA | 7 | 0.001 | 0 | 0 | 7 | 0.001 |
| SINE/Deu | 6 | 0.001 | 0 | 0 | 7 | 0.001 |
| SINE/B2 | 3 | 0 | 0 | 0 | 6 | 0.001 |
| SINE/tRNA-L2 | 4 | 0 | 0 | 0 | 5 | 0.001 |
| SINE/tRNA-7SL | 3 | 0 | 0 | 0 | 3 | 0 |
| SINE/5S | 2 | 0 | 0 | 0 | 3 | 0 |
| SINE/tRNA-CR1 | 1 | 0 | 0 | 0 | 2 | 0 |
| SINE/Alu | 1 | 0 | 0 | 0 | 1 | 0 |
| SINE/Mermaid | 1 | 0 | 0 | 0 | 1 | 0 |
| SINE/RTE | 1 | 0 | 0 | 0 | 1 | 0 |
| SINE/V | 1 | 0 | 0 | 0 | 1 | 0 |
| Others | 281241 | 24.864 | 17611 | 2.289 | 472322 | 57.33 |
| Other/Composite | 2 | 0 | 0 | 0 | 3 | 0 |
| RC/Helitron | 2969 | 0.262 | 750 | 0.097 | 3824 | 0.464 |
| Satellite | 831 | 0.073 | 1033 | 0.134 | 0 | 0 |
| Satellite/telo | 12 | 0.001 | 15 | 0.002 | 0 | 0 |
| TandemRepeat | 261960 | 23.159 | 0 | 0 | 468494 | 56.866 |
| Unknown | 15467 | 1.367 | 15813 | 2.055 | 1 | 0 |

**Table S6. General statistics of** **for the genomes used by homolog-based method.**

| **Gene set** | | **Total** | **Exon number** | **cds length (bp)** | **mRNA length (bp)** | **Exons per gene** | **Exon length (bp)** | **Intron length (bp)** |
| --- | --- | --- | --- | --- | --- | --- | --- | --- |
| **Reference Species** | ***Bos taurus*** | 19,994 | 191,129 | 1,595 | 35,138 | 9.56 | 166 | 3,918 |
|  | ***Canis familiaris*** | 19,258 | 192,445 | 1,592 | 30,639 | 9.99 | 159 | 3,229 |
|  | ***Homo sapiens*** | 21,849 | 206,236 | 1,635 | 46,300 | 9.43 | 173 | 5,292 |
|  | ***Sus scrofa*** | 23,118 | 178,745 | 1,405 | 27,497 | 8.73 | 160 | 3,374 |
|  | ***Rattus norvegicus*** | 32,971 | 323,178 | 1,557 | 36,766 | 9.80 | 158 | 4,000 |
|  | ***Ovis aries*** | 23,220 | 19,7086 | 1,422 | 40,949 | 8.49 | 167 | 5,278 |

**Table S7 Number of all kinds of non-coding RNA**

| **Type** | | **Copy(w)** | **Average length(bp)** | **Total length(bp)** | **% of genome** |
| --- | --- | --- | --- | --- | --- |
| miRNA | | 16,305 | 99.32 | 1619488 | 0.057 |
| tRNA | | 29,821 | 73.73 | 2198765 | 0.077 |
| rRNA | rRNA | 2,357 | 87.14 | 205398 | 0.0072 |
|  | 18S | 636 | 95.01 | 60425 | 0.0021 |
|  | 28S | 1,137 | 92.22 | 104852 | 0.0037 |
|  | 5.8S | 56 | 71.39 | 3998 | 0.00014 |
|  | 5S | 528 | 68.41 | 36123 | 0.0013 |
| snRNA | snRNA | 1,380 | 113.49 | 156612 | 0.0055 |
|  | CD-box | 286 | 89.75 | 25669 | 0.00090 |
|  | HACA-boc | 258 | 134.85 | 34792 | 0.0012 |
|  | splicing | 801 | 114.89 | 92024 | 0.0032 |

**Table S8 Number of Genes with Homology or Functional Classification by each Method**

|  | **Number** | **Percent(%)** |
| --- | --- | --- |
| Total | 26,667 | - |
| Annotated | 25,916 | 97.18 |
| SwissProt | 22,685 | 85.07 |
| TrEMBL | 25,878 | 97.04 |
| InterPro | 21,798 | 81.74 |
| KEGG | 14,550 | 54.56 |
| GO | 17,704 | 66.39 |
| Unannotated | 751 | 2.82 |

**
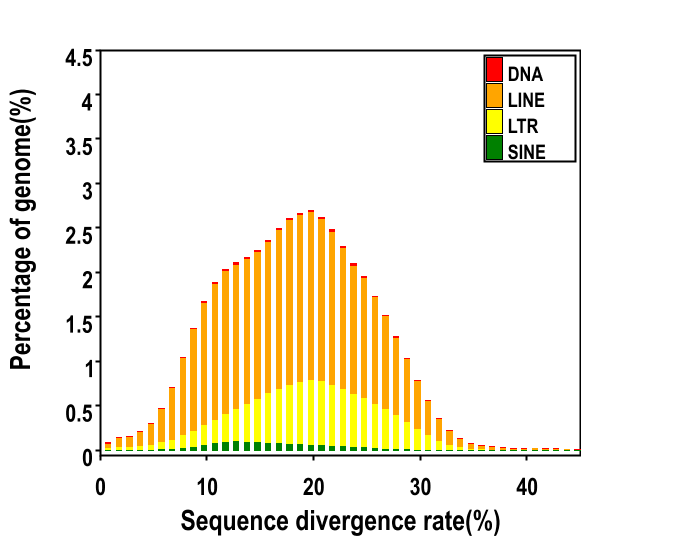
**

**Figure S1.** Distribution of Divergence Rate of each Type of *Bos frontalis*’s TE (*De novo*). The divergence rate was calculated between the identified TE elements in the genome by de novo method and the consensus sequence in the predicted TE library.

**
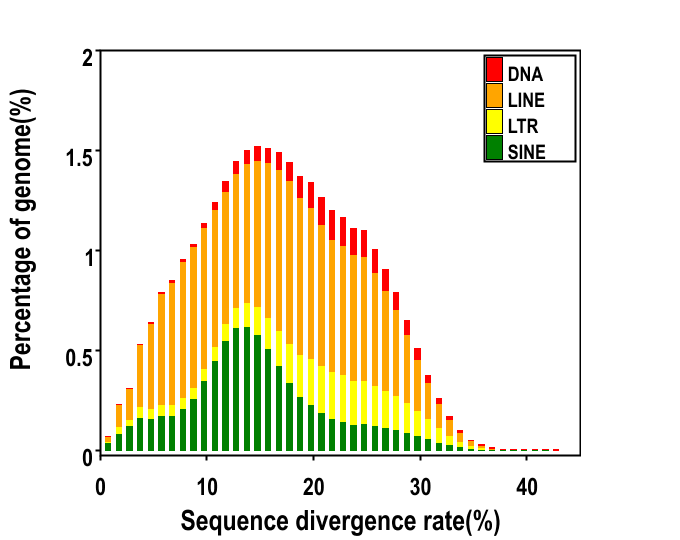
**

**Figure S2.** Distribution of Divergence Rate of each Type of *Bos frontalis*’s TE (Repeatmasker). The divergence rate was calculated between the identified TE elements in the genome by homology-based method and the consensus sequence in the Repbase.


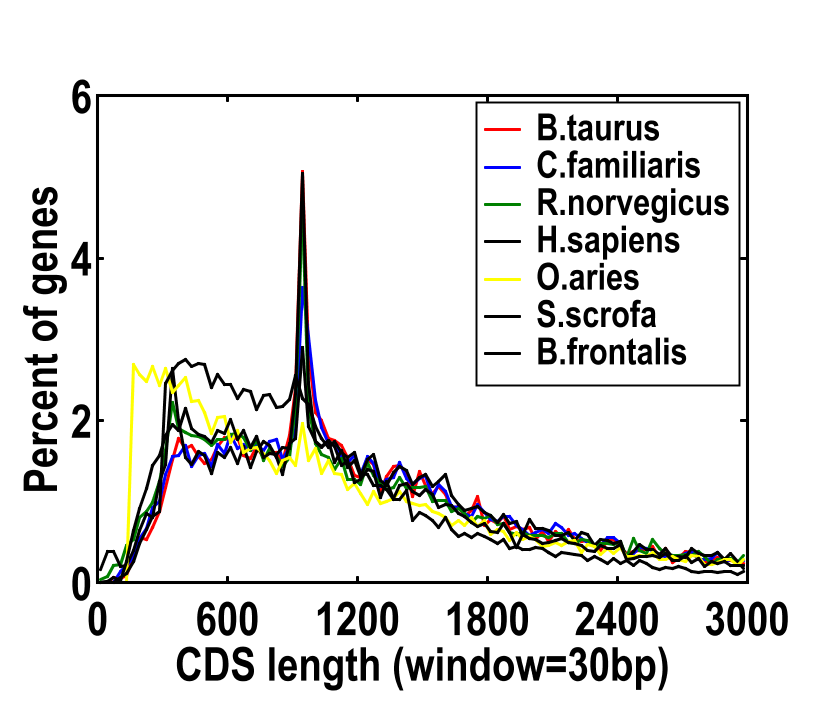

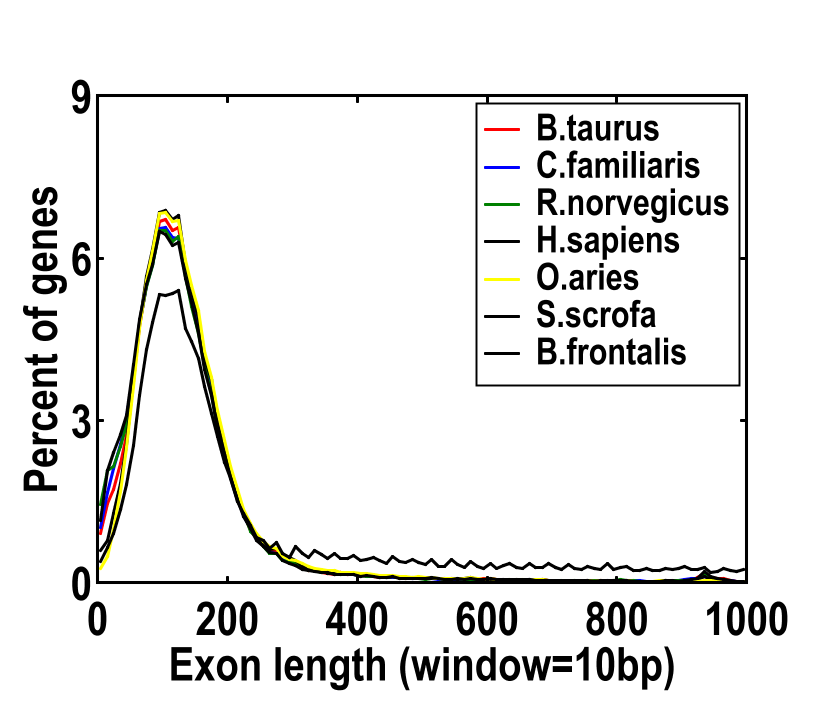


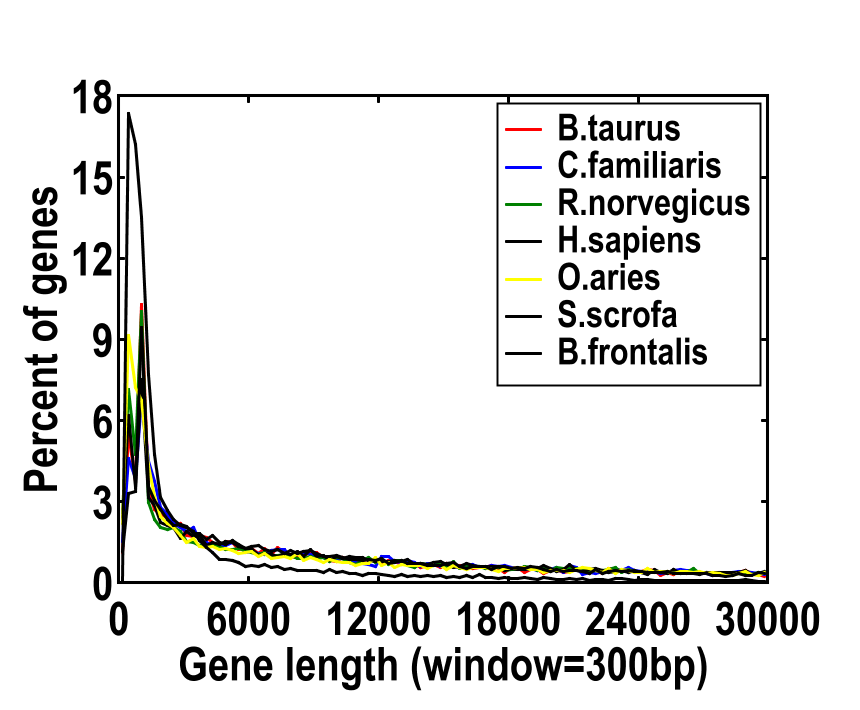

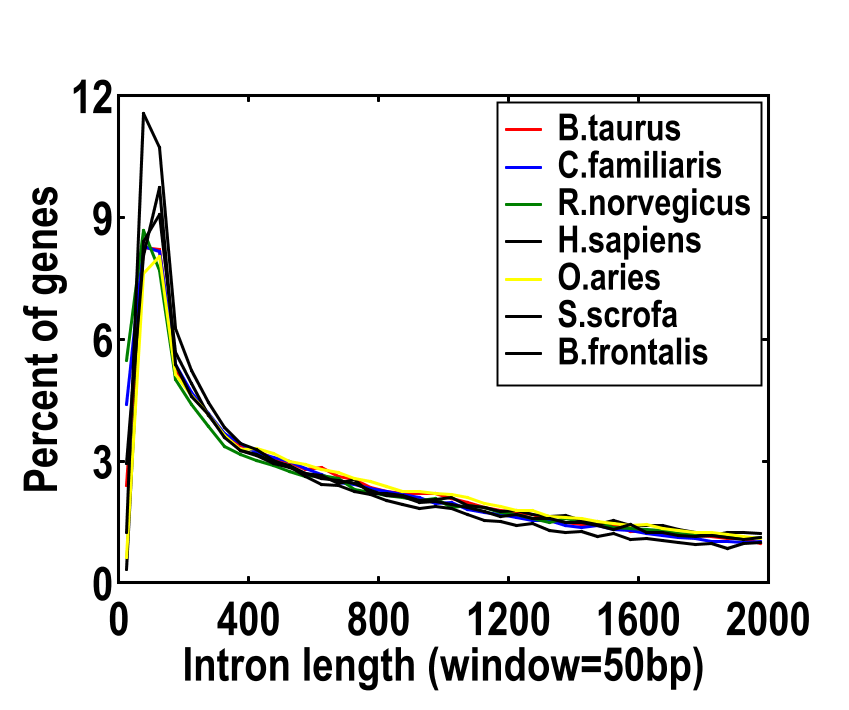


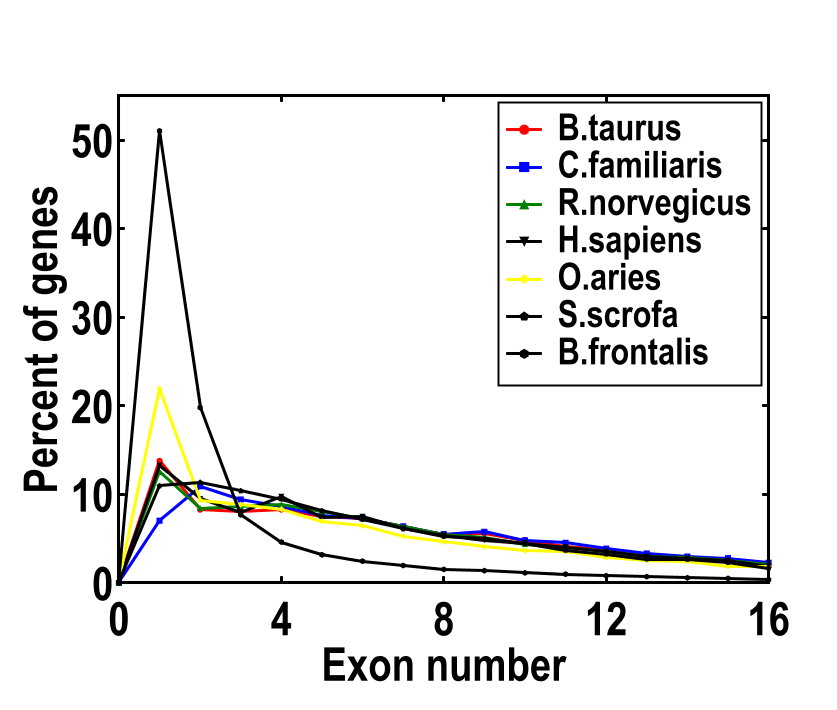


**Figure S3.** Comparisons of gene parameters among *Bos frontalis* (*B. frontalis*), *Bos grunniens* (*B. grunniens*), *Canis familiaris* (*C. familiaris*), *Homo sapiens* (*H. apiens*)，*Sus scrofa* (*S. scrofa*), *Rattus norvegicus* (*R. norvegicus*) and *Ovis aries* (*O. aries*) genomes.

**Figure S4.** Maximum likelihood tree of gayal and other bovine species constructed using cytb


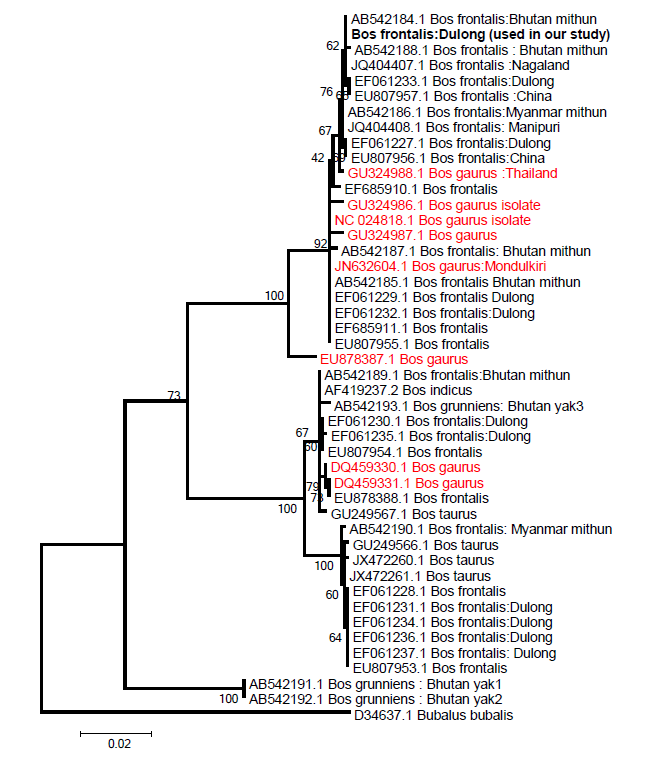

Supplement: Supplement Figures and Tables [file gix094_supp.doc]
