# Supplementary material for: Draft genome of the gayal, Bos frontalis
Source: Gigascience. 2017 Oct 5;6(11):1–7. doi: 10.1093/gigascience/gix094 (PMC5710521; doi:10.1093/gigascience/gix094)
Supplement: Response-to-Reviewer-Comments_Original-Submission.pdf [file gix094_response-to-reviewer-comments_original-submission.pdf]

Author's Response To Reviewer Comments

Response to editor:
Your manuscript "Draft genome of the gayal, Bos frontalis" (GIGA-D-17-00116) has been assessed by our reviewers. Although it is of interest, we are unable to consider it for publication in its current form. The reviewers have raised a number of points which we believe would improve the manuscript and may allow a revised version to be published in GigaScience. Their reports, together with any other comments, are below. In particular, the reviewers point out that the previous literature in this field must be referenced more completely and accurately. Given the previous, published work on genome sequencing of this species, you should also explain better what the novel contribution of your study is.
Reply:
Thanks the editor for handling our manuscript and comments. We found the comments and suggestions very helpful. We have revise the manuscripts carefully, improving literature review and referencing, as wells as clarifying sample origin and novelty of our research. We believe the revisions have greatly improved our manuscript for publication in your reputable journal.

Please pay particular attention to point 7) of reviewer 2 regarding the inferred genome size - this may need careful re-assessment.
Reply:
Thanks for the comment. In the previous estimation, we used raw sequencing reads (without filtration) to infer the K-mer frequency and genome size. We have corrected this mistake and re-assessed the genome size using only the clean reads that passed quality filtration in the genome assembly. The newly estimated genome size is 3.15Gb, still slightly larger than what we assembled (2.85Gb). We have also illustrated and discussed in our responses the discrepancies that commonly occur between K-mer estimated and assembled genome sizes. Please full details in the response to point 4) of reviewer 1 and point 7) of reviewer 2.

Please also provide more details regarding the origin of the sample, and address all other points of the reviewers.
Reply:
We have provided more details on sample origin. The gayal used in this study originated from Dulong, a city in Yunnan province, China. It is currently reared in Yunnan Academy of Grassland and Animal Science for breeding and research purposes. Karyotype examination showed it has 2n=58 chromosomes (see figure2). We have addressed all the points by the reviewers in the one by one response below.

Response to reviewers:
Reviewer #1:
1. Average exon in text is 3.27 where as in corresponding table it is 7.19
Reply:
We are very sorry for the mistake. We predicted genes using both homolog and de novo based methods. Both genes set were subsequently merged using glean to produce the final gene set, in which average exons per gene is 3.27. In the homolog method, using Bos taurus as closed species to search again gayal genome, we predicted 19,666 protein coding genes with average exons of 7.19 per gene. We have made appropriate revisions for consistency and clarity.

2. Reference for buffalo assembly is missing from references
Reply:
We are sorry for this oversight; we have added the reference accordingly.

3. References need to be rechecked as per text
Reply:
Thanks to the reviewer for the comment. We have carefully revised the references one by one.

4. There is a need to re-look into figure of 3.7gb as the genome size of Mithun
Reply:
Thanks for the comment. In the previous estimation, we used raw sequencing reads (without filtration) to infer the K-mer frequency and genome size. We have corrected this mistake and re-assessed the genome size using only the clean reads that passed quality filtration in the genome assembly. The newly estimated genome size is 3.15Gb, still slightly larger than what we assembled (2.85Gb). However, minimal discrepancies between K-mer estimated and assembled genome sizes is a common occurrence in NGS studies (Yim et al. Nat Genet. 2014;46(1):88-92; Wang et al. Gigascience. 2017;doi: 10.1093/gigascience/gix016; Fan et al. Nat Commun. 2013;4:1426; Gao et al. Gigascience. 2017; doi:10.1093/gigascience/gix041). We think low sequencing bases likely lead to over estimation of genome size. In addition, as demonstrated by the previous gayal sequencing (Mei et al.2016) and our current work, there is high heterozygosity in the gayal genome, which also likely influence its genome size estimation.

Reviewer #2:
This is a well-written account of a whole-genome sequence of the gayal, a most interesting bovine species. However, it should become clear what is the novelty of the results relative to an earlier report on a WGS of the same species. Furthermore, more details about the sample origin should be given, while referencing to the literature about the gayal is superficial and even incorrect. We recommend a major revision.
Reply:
We thank the reviewer very much for the constructive analysis and comments on our manuscript. We have followed the suggestions of the reviewer to revise our manuscript, particularly discussing the novelty of the results relative previous research on gayal and other bovine relatives, explaining sample origin, as well as revising the literature review and references. Please see below a detailed point by point response to the detailed comments.

Detailed comments
1. As cited, Mei et al. (2016) already published a gayal WGS, so a separate publication on another sequence should be justified, for instance because of a better coverage, contig and scaffold statistics and gene coverage.
Reply:
Thanks the reviewer for the comment. As stated by the reviewer, last year, Mei et al. published a study in which they re-sequenced gayal WGS. They generated 36.3Gb genome sequence data with an average sequencing depth of 13.06X after mapping the sequencing reads to cattle reference genome. Their analysis was therefore based on SNPs obtained by mapping gayal

genome to cow reference genome. They further constructed phylogenic trees using a subset of only 20 randomly selected single ortholog copy genes in Bos taurus, Bos mutus (wild yak) and Bubalus bubalis genomes, placing gayal off B. mutus and B. taurus. While we appreciate the importance of their work and other preceding partial genome research on gayal, we also take note that they used a resequencing approach for species that does not have a reference genome, forcing them to map the gayal sequencing reads to a cattle genome. Their study, as well as our own analysis, shows that gayal has a high heterozygosity and is far divergent from cattle. Hence, using cattle reference when mapping gayal sequencing reads is definitely likely to produce biases during alignment and SNP calling procedures. In addition, they did not determine/report the karyotype of the gayal they used. This is an important matter for ongoing research on gayal as gayal hybrids are common in China. In our study, we have tried to take care of these limitations. We used a female gayal with 58 chromosomes to perform high coverage whole genome sequencing (350.38Gb raw data) with libraries constructed based on different insert sizes, and then performed de novo assembly. Besides the detailed analysis and description of the genome properties, we also state the karyotype of the gayal used and its phylogenetic relationship with other bovines (validated by complete mtDNA gayal sequences generated by Sanger sequencing method). Overall, our study represents the pioneer de novo assembly of the gayal whole genome, and Sanger sequencing of its complete mtDNA. Our study therefore presents a suitable reference genome for future studies on gayal, plus other important resources and insights that will facilitate research on gayal and other bovine species.

We have concisely included these descriptions in the revised manuscript.

2. The geographic origin sample of the sample should be specified. Chinese gayals, or Dulong cattle, are known to harbor zebu or taurine mtDNA (Gou et al. 2010, J.Anim.Breeding Genet. 127, 154-160; Mei et al. 2016) and may very well differ from individuals with an Indian origin.
Reply:
Thanks the reviewer for the comment. We have provided more details on sample origin. The gayal used in this study originated from Dulong, a city in Yunnan province, China. It is currently reared in Yunnan Academy of Grassland and Animal Science for breeding and research purposes. As suggested, we have explained the sample origin more clearly and cited these references appropriately in our revised manuscript.

3. For this reason the mtDNA sequence should be retrieved and compared to the several available gayal mtDNA sequences published previously.
Reply:
As suggested, we searched NCBI-Nucleotide database for published mtDNA sequences gayal. Unfortunately, there is no complete mtDNA assembly available for gayal, except partial mtDNA sequences like D-loop, cytb, and 16s. Considering the lower ability of NGS to accurately recover duplicated sequences that characterize regions like the D-loop in mtDNA, we sequenced complete mtDNA from the gayal in our study using Sanger method. We then downloaded sequences of mtDNA for gayal and other Bovine species, and constructed phylogenic trees. Bellow are trees constructed using maximum likelihood method based on complete mtDNA (see figure 5) and cytb (see figure S4) sequences. We observed that the gayal in our study clustered with gaurs and gayal from Dulong, Myanmar, Bhutan, and Manipuri. We have submitted the new complete mtDNA sequence to the Genbank and added this analysis in our revised

manuscript.

4. Thai and Malaysian gaur have indeed a 2n=56 karyotype, but Indian gaur, which occurs in the geographic area overlapping with the range of the gayals, has 2n=58 (Winter et al., 1984, Res Vet Sci 36: 276-283; Gallagher et al., 1992, J Hered 83:287-298; Mastromonaco et al., 2004, Chromosome Res. 2:725-31).
Reply:
We thanks the reviewer for this comment. We agree with the reviewer that determining and reporting the karyotype of gayal is important due to these cryptic variations. Besides reporting the karyotype of the gayal in our study, we have revised our manuscript to reflect the insights offered by the reviewer plus the appropriate citations.

5. The cited references (5,14) do not show that gaur x gayal male offspring are sterile. Although I could not find literature about the outcome of this hybrid cross, it is generally assumed that gayal is the domestic form of the gayal, also because they have similar mtDNA and Y-chromosomal DNA sequences (Hassanin et al. 2012, C.R.Biologies 335:32-50; Nijman et al. 2008, Cladistics 24:723-726).
Reply:
We are sorry for the oversight. We have revised this description to maintain only the details that have a solid literature backing. Thanks for the comment.

6. The URL reference [19] of the academic thesis describing the American bison WGS is still inaccessible. I guess that this WGS has been downloaded from Genbank, which should be made clear.
Reply:
Thanks to the reviewer for the comment. We download the sequence from Genbank and have revised the citation appropriately.

7. The inferred genome size for the gayal of 3.7 Gbp, larger than the genome of any related mammalian species, is not believable and not consistent with the gene coverage.
Reply:
We thank the reviewer for this important observation. In the previous estimation, we used raw sequencing reads (without filtration) to infer the K-mer frequency and genome size. We have corrected this mistake and re-assessed the genome size using only the clean reads that passed quality filtration in the genome assembly. The newly estimated genome size is 3.15Gb, still slightly larger than what we assembled (2.85Gb). However, minimal discrepancies between K-mer estimated and assembled genome sizes is a common occurrence in NGS studies (Yim et al. Nat Genet. 2014;46(1):88-92; Wang et al. Gigascience. 2017;doi: 10.1093/gigascience/gix016; Fan et al. Nat Commun. 2013;4:1426; Gao et al. Gigascience. 2017; doi:10.1093/gigascience/gix041). We think that low quality sequencing bases likely lead to over estimation of genome size. In addition, as demonstrated by the previous gayal sequencing (Mei et al.2016) and our current work, there is high heterozygosity in the gayal genome, which also likely influence its genome size estimation.

8. It may be interesting to compare the recovered DNA repeats with those from the bovine WGS.
Reply:

Thanks to the reviewer for the comment. It is an interesting topic to compare the repeats in different bovine species. However, whole genome solely based on NGS has low efficiency to assemble repeat sequences (Wang et al.2016. Nature Genetics 48(9): 972-3). In addition, many of these bovine genomes are generated without uniform sequencing platform and assembly strategies. Further, the repeats predictions do not follow a harmonized pipeline, hence remain just draft genomes. It is difficult to distinguish the lose or increase of repeats in one species to be attributable to evolution or from technique/sequencing effects. The main reach of the current study is providing a comprehensive genetic resources and a draft reference genome for gayal to facilitate future research. We believe that in future, when high quality genomes for the bovine species become available, it will be fascinating to retrieve and compare DNA repeats evolution among the bovine species.

9. page 9 last line: vertebrata > vertebrate.
Reply:
Thanks, we have revised this accordingly.
