# Supplementary material for: Draft genome of the gayal, Bos frontalis
Source: Gigascience. 2017 Oct 5;6(11):1–7. doi: 10.1093/gigascience/gix094 (PMC5710521; doi:10.1093/gigascience/gix094)
Supplement: Reviewer-2-Report-(Original-Submission).pdf [file gix094_reviewer-2-report-(original-submission).pdf]

Reviewer Report

Title: Draft genome of the gayal, Bos frontalis

Version: Original Submission **Date:** 6/20/2017

Reviewer name: Johannes Lenstra

Reviewer Comments to Author:

This is a well-written account of a whole-genome sequence of the gayal, a most interesting bovine species. However, it should become clear what is the novelty of the results relative to an earlier report on a WGS of the same species. Furthermore, more details about the sample origin should be given, while referencing to the literature about the gayal is superficial and even incorrect. We recommend a major revision.Detailed comments1. As cited, Mei et al. (2016) already published a gayal WGS, so a separate publication on another sequence should be justified, for instance because of a better coverage, contig and scaffold statistics and gene coverage.2. The geographic origin sample of the sample should be specified. Chinese gayals, or Dulong cattle, are known to harbor zebu or taurine mtDNA (Gou et al. 2010, J.Anim.Breeding Genet. 127, 154-160; Mei et al. 2016) and may very well differ from individuals with an Indian origin.3. For this reason the mtDNA sequence should be retrieved and compared to the several available gayal mtDNA sequences published previously.4. Thai and Malaysian gaur have indeed a 2n=56 karyotype, but Indian gaur, which occurs in the geographic area overlapping with the range of the gayals, has 2n=58 (Winter et al., 1984, Res Vet Sci 36: 276-283; Gallagher et al., 1992, J Hered 83:287-298; Mastromonaco et al., 2004, Chromosome Res. 2:725-31).5. The cited references (5,14) do not show that gaur x gayal male offsrping are sterile. Although I could not find literature about the outcome of this hybrid cross, it is generally assumed that gayal is the domestic form of the gayal, also because they have similar mtDNA and Y-chromosomal DNA sequences (Hassanin et al. 2012, C.R.Biologies 335:32-50; Nijman et al. 2008, Cladistics 24:723-726).6. The URL reference [19] of the academic thesis describing the American bison WGS is still inaccessible. I guess that this WGS has been downloaded from Genbank, which should be made clear.7. The inferred genome size for the gayal of 3.7 Gbp, larger than the genome of any related mammalian species, is not believable and not consistent with the gene coverage.8. It may be interesting to compare the recovered DNA repeats with those from the bovine WGS.9. page 9 last line: vertebrata > vertebrate.

Level of Interest

Please indicate how interesting you found the manuscript: An article of importance in its field

Quality of Written English

Please indicate the quality of language in the manuscript: Acceptable

Declaration of Competing Interests

Please complete a declaration of competing interests, considering the following questions:

- Have you in the past five years received reimbursements, fees, funding, or salary from an organisation that may in any way gain or lose financially from the publication of this manuscript, either now or in the future?
- Do you hold any stocks or shares in an organisation that may in any way gain or lose financially from the publication of this manuscript, either now or in the future?
- Do you hold or are you currently applying for any patents relating to the content of the manuscript?
- Have you received reimbursements, fees, funding, or salary from an organization that holds or has applied for patents relating to the content of the manuscript?
- Do you have any other financial competing interests?
- Do you have any non-financial competing interests in relation to this paper?

If you can answer no to all of the above, write 'I declare that I have no competing interests' below. If your reply is yes to any, please give details below.

I am collaborating on a high-quality whole-genome sequence of the same species, but welcome this manuscript

I agree to the open peer review policy of the journal. I understand that my name will be included on my report to the authors and, if the manuscript is accepted for publication, my named report including any attachments I upload will be posted on the website along with the authors' responses. I agree for my report to be made available under an Open Access Creative Commons CC-BY license (http://creativecommons.org/licenses/by/4.0/). I understand that any comments which I do not wish to be included in my named report can be included as confidential comments to the editors, which will not be published.

I agree to the open peer review policy of the journal
